# Tryptophan metabolic gatekeeping in epithelial repair: GPR35-KLF5 circuitry decodes mucosal damage signals for repair programming

**DOI:** 10.1038/s41419-025-08237-0

**Published:** 2026-01-09

**Authors:** Biao Xie, Meimei Wang, Yaping Xiao, Xin Zhang, Meng Liu, Jie Miao, Yunfei Mo, Hongxin Liu, Jihui Wang, Fengguo Xu, Di Wang

**Affiliations:** 1 School of Life and Health Technology, Dongguan University of Technology, Dongguan, 523808 P.R. China; 2 Department of Gastroenterology, Guangzhou Eighth People’s Hospital, Guangzhou Medical University, Guangzhou, 510440 P.R. China; 3 Guangzhou Key Laboratory of Clinical Pathogen Research for Infectious Diseases, Guangzhou Eighth People’s Hospital, Guangzhou Medical University, Guangzhou, 510440 P.R. China; 4 Key Laboratory of Drug Quality Control and Pharmacovigilance (Ministry of Education), State Key Laboratory of Natural Medicine, China Pharmaceutical University, Nanjing, 210009 P.R. China

**Keywords:** Ulcerative colitis, Mechanisms of disease

## Abstract

The impaired repair of intestinal mucosal damage is an important pathological feature of ulcerative colitis (UC). The critical role of intestinal epithelial cells (IECs) proliferation and migration in the repair of damaged mucosal epithelium has been well established. However, the molecular circuitry that decodes IECs sense intestinal mucosal damage signals to initiate and drive repair program remains elusive. Here, we identify a tryptophan (Trp) metabolic gatekeeping mechanism wherein G protein-coupled receptor 35 (GPR35) senses intestinal mucosal damage through monitoring Trp-kynurenine (KYN)-kynurenic acid (KA) axis metabolism with a unique “sandwich” structural binding mode. We delineate a GPR35-Kruppel-like factor 5 (KLF5) regulatory circuit in which KLF5 serves as the central effector, translating GPR35-mediated KA sensing into repair programming through PI3K-AKT-mTOR signaling cascade. This circuitry precisely orchestrates IECs proliferation and migration by regulating KLF5-dependent gene expression networks that essential for restoring damaged mucosa. Once this metabolic gatekeeping system is disrupted, either through impaired GPR35-mediated KA sensing or defective signal transduction, compromises damage signal decoding, leading to inadequate repair responses. Such dysregulation results in delayed intestinal mucosal repair and exacerbation of tissue damage. Our findings highlight GPR35 as a surveillant of abnormal Trp-KYN-KA axis metabolism, enabling IECs to detect intestinal mucosal damage and orchestrate repair through KLF5 response. This provides important implications for UC prevention and treatment by targeting GPR35-KLF5 circuit.

## Introduction

Ulcerative colitis (UC) is a persistent and recurring chronic inflammatory disease of the intestinal tract, which is listed by the World Health Organization as one of the modern difficult to treat diseases [1]. The damage of intestinal mucosal barrier function is considered to be the initiating event of UC [2, 3]. Promoting the repair of intestinal mucosal damage and preserving the integrity of the intestinal barrier have emerged as primary objectives in current UC therapy.

The repair of intestinal mucosal injury is a crucial defensive and adaptive response to external stimuli, enabling the restoration of damaged tissues through cellular regeneration and tissue reconstruction [4]. At present, the repair process of intestinal mucosal injury is regarded as a process of proliferation, migration and differentiation of various cells involved in repair, especially IECs [5]. The IECs are not only the first line of defense against pathogen invasion, but also the hub of internal and external environmental signal transduction, and play a crucial role in regulating intestinal mucosal innate immune defense and damage repair. The proliferation and migration of normal IECs to the damaged site constitute the physiological foundation for the repair of intestinal mucosal damage [6, 7]. Indeed, the function and rationales of IECs proliferation and migration in the process of damaged intestinal mucosal repair have been well described [8]. However, it remains a mystery how the IECs sense intestinal mucosal damage signals, and through which mechanisms they initiate and drive cell proliferation and migration to repair of intestinal mucosal damage after sensing. These processes are the initial links for IECs to participate in the repair of intestinal mucosal damage, which determine the start of damaged-intestinal mucosal repair. Uncovering their mechanism is of great significance for understanding UC pathogenesis and therapeutic intervention.

Physiologically, cells respond precisely to molecular changes in the body microenvironment via biosensors to rapidly adapt to physiological and pathological alterations [9, 10]. Most destructions to intestinal homeostasis can be transmitted to the host via metabolites and their monitors. Although the underlying initiation and signal conversion mechanisms have not been fully defined, a feedback response would be driven definitively to resist the stimulus and redress the abnormities [11]. Among the various metabolic sensors, the G protein-coupled receptors (GPRs) have received considerable attention for their unique function in establishing communication between the surroundings and the host [12, 13]. Thereinto, GPR35 is highly expressed in gastrointestinal barrier systems with distinct tissue specificity [14], and its single-nucleotide polymorphisms has been established as a risk factor associated with the occurrence of inflammatory bowel disease, suggesting the critical role it plays in maintaining intestinal function and homeostasis [15]. Indeed, our previous studies indicated that UC occurrence, a significant upregulation of tryptophan (Trp)-kynurenine (KYN)-kynurenic acid (KA) axis metabolism was caused by inflammatory factors released by macrophages, leading to a significant accumulation of KA in damaged colonic tissue. We also identified that GPR35 is a key guardian of the KA level and a core component of the defense responses against intestinal damage [16]. However, regrettably, its role and molecular circuitry in damage decoding and repair programming remain enigmatic.

In the present study, we demonstrate a Trp metabolic gatekeeping mechanism orchestrated by GPR35, which decodes intestinal mucosal damage through surveilling Trp-KYN-KA axis metabolism perturbations with a unique “sandwich” structural interaction. The PI3K-AKT-mTOR axis integrates KA flux into KLF5-dependent repair program, triggering transcriptional reprogramming. KLF5 emerges as the transcriptional orchestrator of epithelial restitution, upregulating a repair-associated gene network that coordinates IECs proliferation and migration, thereby executing the repair program encoded by Trp metabolic inputs.

## Results

### KLF5 is a key effector of GPR35

**Fig. 1:**
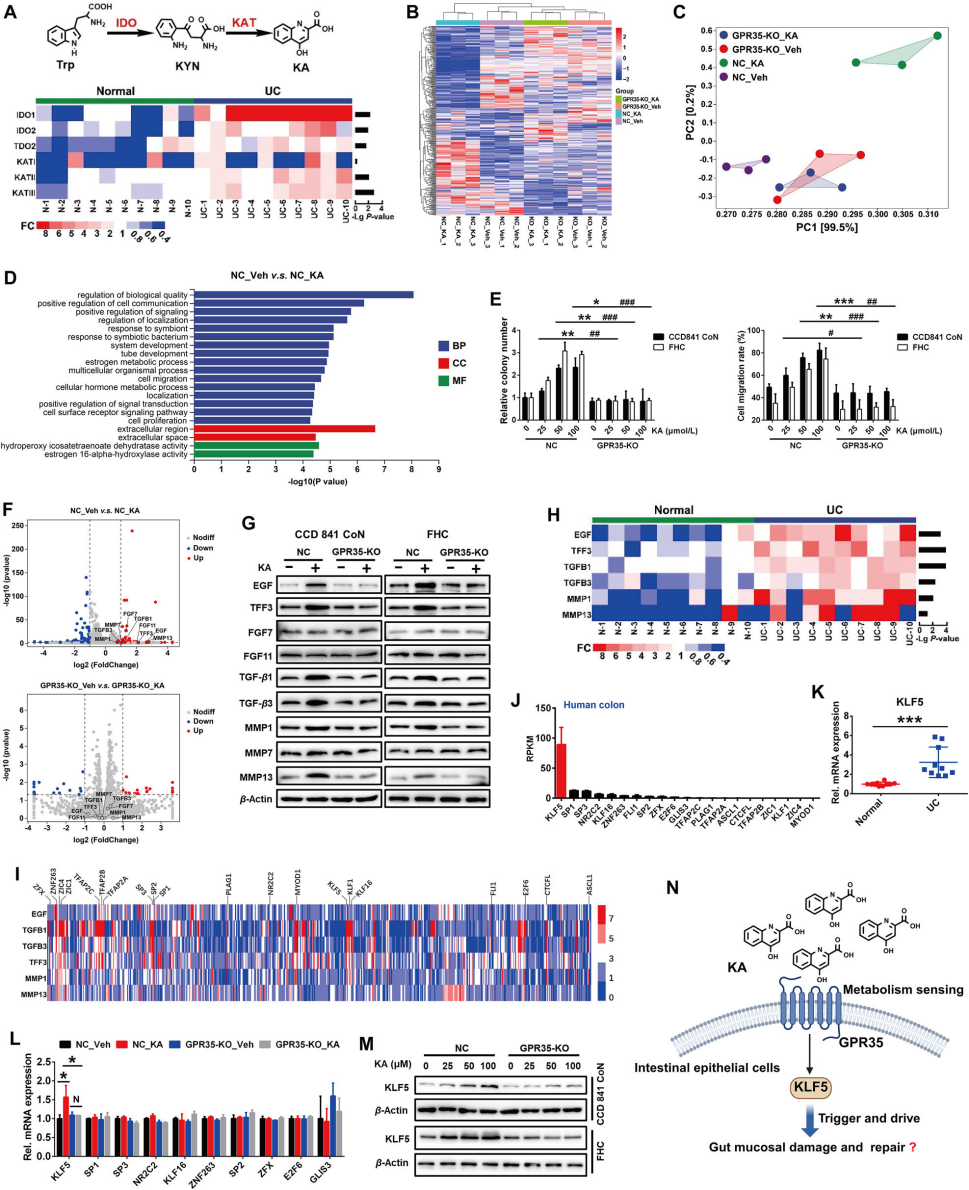
KLF5 mediates IECs proliferation and migration driven by GPR35. **A** RNA-Seq analysis of the expression changes of Trp-KYN-KA axis metabolism key enzyme gene in the colon of UC patients (*n* = 10). **B** Hierarchical cluster analysis of differential gene expression in GPR35 knockout CCD841 CoN cells that were treated with KA 100 μmol/L for 48 h (|Fold-change | > 2, *P* < 0.05, *n* = 3). **C** Principal components analysis (PCA) of the differential genes. **D** Significant enriched top 20 GO terms between negative control group and KA treatment group based on the functions of differential genes. BP, biological process; CC, cellular component; MF, molecular function. **E** Quantitative analysis of the effect of GPR35 knockout on KA induced monolayer IECs proliferation (left panel) and migration (right panel) (means ± SD, * and # are represented statistically significance of the data from CCD841 CoN and FHC cells respectively, *^/#^*P* < 0.05, **^/##^*P* < 0.01, ***^/###^*P* < 0.001, Student’s *t* test, *n* = 3). **F** Volcano map of differentially expressed genes. Red dots indicate significantly upregulated genes and blue dots indicate significantly downregulated genes. **G** Western blot analyzes the effect of KA on the expression of proliferation and migration related proteins in GPR35 normal and deficient IECs. **H** The mRNA expression changes of proliferation and migration related proteins in the colon of UC patients (*n* = 10). **I** Transcription factor binding sites analysis of the gene promoter region based on EPD database. **J** Gene expression levels of the top 20 transcription factors in the number of binding sites in human colon tissues were analyzed based on NCBI Gene database (*n* = 5). RPKM, reads per kilobase per million mapped reads. **K** The expression changes of KLF5 mRNA in the colon of UC patients (means ± SD, ***P < 0.001, Student’s *t* test, *n* = 10). **L** The effect of KA on the mRNA expression of the focused transcription factors in GPR35 normal and deficient IECs (means ± SD, *P < 0.05, Student’s *t* test, *n* = 3). **M** Western blot analysis of the effect of KA on the expression of KLF5 protein in GPR35 normal and deficient IECs. **N** The proposed schematic form of GPR35-mediated KA sensing trigger and drives damaged-intestinal mucosal repair through KLF5 (Created with BioRender.com, Agreement number: ZP28T5AKVM).

The damage of intestinal mucosal barrier function is considered to be the initiating event of UC [2, 17, 18]. The promotion of repair for damaged intestinal mucosa and the preservation of intestinal barrier integrity hold significant clinical importance in the prevention and treatment of UC, thus emerging as primary objectives in UC management. RNA-Seq of biopsy samples from 10 UC patients, including both pathological and normal colonic mucosal tissue, showed significant up-regulation of indoleamine 2,3-dioxygenase (IDO) and kynurenine aminotransferase (KYN) expression, key enzymes in Trp-KYN-KA axis metabolism, after UC occurrence (Fig. 1A), suggesting KYN metabolism pathway of tryptophan was increased, which was consistent with the results of our previous targeted metabolomics analysis [16]. Abnormal metabolism is an important signal of disease occurrence. The communication between metabolites and their sensors is a core component of the body to perceive disease occurrence, initiating feedback regulatory mechanisms and adaptive responses. Our previous study demonstrated that environmental receptor GPR35-mediated aberrant Trp metabolism sensing is an important element of defensive response against intestinal damage. However, the rationale of GPR35 initiates and drives the repair program of damaged intestinal mucosa after sensing the aberrance of Trp metabolism remains enigmatic.

To further elucidate the triggers and driving circuitry underlying damaged intestinal mucosal repair, we knocked out GPR35 gene in colon epithelial cells (Fig. S1). RNA-Seq analysis results showed that KA caused an obvious gene expression reprogramming in colon epithelial cell, and GPR35-KO resulted in significant reversal of the alterations (Fig. 1B, C). Gene Ontology (GO) analysis showed that the differentially expressed genes were significantly enriched in the processes related to cell proliferation and migration (Fig. 1D; Fig. S2). The proliferation and migration of normal IECs to the site of injury constitute crucial physiological mechanisms for repairing damage to the intestinal mucosa. Indeed, KA promoted IECs colony formation and wound healing in a dosage dependent manner at cellular level, while the effects were lost in GPR35 gene deletion IECs (Fig. 1E), indicating the crucial role of GPR35-mediated KA sensing in the repair of intestinal mucosal damage via regulating IECs proliferation and migration.

Subsequently, we analyzed the differential gene to further insights into the downstream key effector of GPR35 that regulate the repair of intestinal mucosal injury, and observed a significant increase in the expressions of EGF, FGF7, FGF11, TGFB1, TGFB3, TFF3, MMP1, MMP7 and MMP13 following KA treatment. GPR35 deletion, the induction of these genes by KA was significantly inhibited (Fig. 1F). In addition, western blot analysis revealed a significant upregulation of EGF, TFF3, TGF-β1/3, and MMP1/13 proteins in IECs following KA treatment, while the effect was blocked in GPR35-deleted IECs (Fig. 1G). Subsequently, we analyzed the clinical samples and observed a significant upregulation of the six protein genes in the pathological tissues of UC patients compared with the normal colonic mucosa (Fig. 1H). Analysis of the transcription factor binding sites in gene promoter region showed that CTCFL, KLFs, *etc*. exhibit multiple binding sites in the promoter regions of these six genes (Fig. 1I). Thereinto, the expression of KLF5 in colon tissue was significantly higher than other transcription factors among the top 20 (Fig. 1J). What’s more, the expression of KLF5 exhibited a significant increase following the occurrence of UC (Fig. 1K). This implies the reprogramming of IECs proliferation and migration related gene expression may be attributed to KLF5. Besides, RNA-Seq and Western blot data revealed a significant upregulation of KLF5 mRNA and protein expression in colon epithelial cells after KA treatment, whereas this effect was notably attenuated in GPR35-KO cells (Fig. 1L, M). These findings suggest that KLF5 possibly serve as a crucial effector in GPR35-mediated KA sensing drives IECs proliferation and migration to facilitate the repair of UC-induced intestinal mucosal damage (Fig. 1N).

### KLF5 executes repair program through orchestrating IECs proliferation and migration

**Fig. 2:**
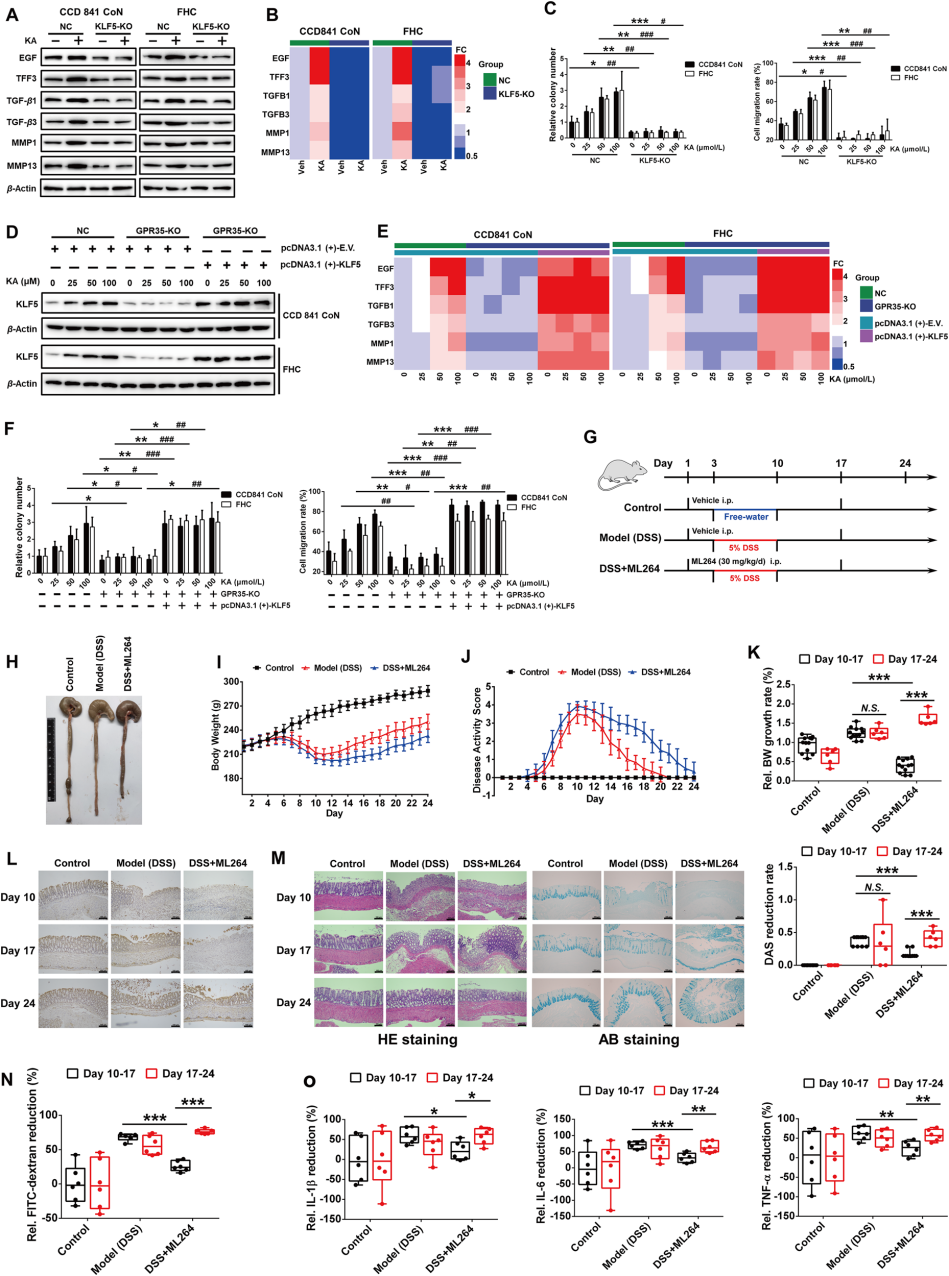
KLF5 plays an important role in maintaining gut homeostasis. **A**, **B** Western blot and RT-PCR analyze the effect of KLF5 knockout on the expression of proliferation and migration related proteins (**A**) and mRNA (**B**) induced by KA (*n* = 3). **C** Quantitative analysis of the effect of KLF5 knockout on KA induced monolayer IECs proliferation (left panel) and migration (right panel) (means ± SD, * and # are represented statistically significance of the data from CCD841 CoN and FHC cells respectively, *^/#^*P* < 0.05, **^/##^*P* < 0.01^,^***^/###^*P* < 0.001, Student’s *t* test, *n* = 3). **D**, **E** Western blot analysis of the effect of KA on KLF5 protein (**D**) and its mediated gene mRNA (**E**) expression in GPR35-KO IECs transfected with KLF5 overexpression plasmid (*n* = 3). **F** Quantitative analysis of the effect of KA on proliferation (left panel) and migration (right panel) of IECs with GPR35-KO and KLF5 overexpression (means ± SD, *^/#^*P* < 0.05, **^/##^*P* < 0.01, ***^/###^*P* < 0.001, Student’s *t* test, *n* = 3). **G** Schematic diagram of ML264, a KLF5 specific inhibitor, intervene DSS-induced rat colitis. **H** Representative images of rat colon tissues on day 10. **I**, **J** The changes of body weight (**I**) and DAS (**J**) of each group rats were monitored twice daily (means ± SD, *n* = 6–18). **K** The relative body weight growth rate (upper panel) and DAS reduction rate (under panel) during the period of day 10–17 and 17–24 were analyzed to characterize the change speed of rat body weight and DAS (means ± SD, ***P < 0.001, Student’s *t* test, *n* = 6–12). **L** Immunohistochemistry analysis of KLF5 protein expression in rat colon tissues, scale bars = 100 μm. **M** HE and AB staining analyses of rat colon tissues. **N**, **O** The relative variational amplitudes of FITC-dextran level in serum (**N**) and inflammatory factor level in colon (**O**) during the period of day 10–17 and 17–24 were analyzed to reflect the recovery speed of intestinal permeability and inflammation (means ± SD, *P < 0.05, **P < 0.01, ***P < 0.001, Student’s *t* test, *n* = 6).

Then, we deleted KLF5 gene to further investigate its role in IECs proliferation and migration (Fig. S3). The results demonstrated that knocking out KLF5 significantly intercepted the promoting effect of KA on cell proliferation and migration, as well as the expression of their related EGF, TFF3, TGF-β1/3, MMP1/13 proteins and mRNA (Fig. 2A–C). The expression of KLF5 protein and its mediated gene expression, as well as IECs proliferation and migration were dose-dependently induced by KA in GPR35 wild-type cells. Overexpression of KLF5 in GPR35-deficient IECs (Fig. 2D) significantly reversed the loss of the regulation of KA on IECs proliferation, migration and their related gene expression caused by GPR35 gene deletion. However, the dose-dependent effect of KA was still unrestored (Fig. 2E, F), suggesting that IECs proliferation and migration are directly associated with KLF5 levels and its mediated gene expression which is strictly regulated by GPR35-mediated KA sensing.

Subsequently, we established dextran sulfate sodium salt (DSS)-induced rat colitis model, as illustrated in Fig. 2G. Although both the model group and ML264, a KLF5 specific inhibitor, treatment group exhibited pathological changes, including progressive fatigue, lethargy, bradykinesia, emaciation, hair tarnish and disheveled, and significantly decreased food and water intake following administration of DSS. Notably, the severity of these symptoms was more pronounced in the ML264 treatment group compared to the model group. Meanwhile, both groups displayed evident pathological conditions in colon tissues including redness, swelling, bleeding, watery intestinal content, and shortened length. These changes were more severe in the ML264 treatment group (Fig. 2H). Through daily monitoring during DSS stimulation for 7 days, rats treated with KLF5 inhibitor showed significant weight reduction and increased disease activity score (DAS) compared to those in the model group (Fig. 2I, J; Fig. S4A). Although the rats in each group demonstrated varying degrees of improvement in body weight and DAS from the termination of DSS stimulation to the completion of ML264 intervention (Day 10–17), analysis of the change rates of body weight and DAS showed that the recovery speed of the both indices in ML264 intervention group were significantly lower than that in the model group (Fig. 2K). Furthermore, following the discontinuation of ML264 intervention (Day 17–24), the KLF5 inhibitor intervention group exhibited a significantly accelerated recovery rate in terms of body weight and DAS (Fig. 2K).

The expression of KLF5 in colon tissue was further analyzed, and the results showed that after DSS stimulation terminated (Day 10), the ML264 intervention group exhibited a significant decrease in KLF5 expression in colon tissues compared to the model group. After DSS intervention terminated 7 days (Day 17), while KLF5 expression in the colon tissue of the model group was restored, it remained unaltered in the ML264 intervention group. Notably, only after the end of ML264 intervention 7 days (Day 24) did the KLF5 level in the colon tissue of ML264 intervention group exhibit a significant restoration (Fig. 2L). The histopathological analysis revealed that model and ML264 intervention groups exhibited severe pathological damage in the colon tissue following DSS intervention (Day 10), including extensive erosion and necrosis of intestinal mucosa, intestinal crypts loss, edema and congestion in the lamina propria, disruption of intestinal barrier integrity, permeability increased, colonic mucus reduced and goblet cell decreased, etc. (Fig. 2M; Fig. S4B). Notably, rats treated with ML264 displayed more severe intestinal mucosa damage compared with the model group. After 7 days following the cessation of DSS stimulation, significant repair of colonic tissue injury was observed in the model group rats, whereas the colon of ML264 intervention group rats still exhibited serious damage. It was only after 7 days following the termination of ML264 intervention that intestinal mucosal injury in this group rats were significantly repaired, with a notably faster rate compared to before the end of ML264 intervention (Fig. 2M, N; Fig. S4B). Additionally, Elisa analysis showed that DSS stimulation resulted in significant elevations in the levels of inflammatory factors IL-1β, IL-6, and TNF-α in the colon of model and ML264 intervention groups rats on day 10 (Fig. S4C). Although the levels of inflammatory factors in the ML264 intervention group were higher than those in the model group, no statistically significant difference was observed, possibly attributed to the extensive tissue damage induced by DSS stimulation in both group rats (Fig. S4C). However, during the 7 days after DSS stimulation terminated (Day 10–17), with the recovery of intestinal mucosal damage, although the levels of inflammatory factors (IL-1β, IL-6, and TNF-α) in the colon tissue decreased to varying degrees in both the model and ML264 intervention groups (Fig. S4C), it was observed that the ML264 intervention group exhibited a significantly lower degree and rate of reduction than model group (Fig. 2O; Fig. S4C). Moreover, there was a significant difference in inflammatory factor levels between these two groups on day 17, with higher levels observed in the ML264 intervention group. Furthermore, the ML264 intervention group exhibited a further reduction of inflammatory factor levels and a more rapid decline in amplitude and rate after ML264 termination (Day17-24) compared to the before (Day 10–17) (Fig. 2O; Fig. S4C). Collectively, these results indicate that KLF5 plays a pivotal role in the repair of intestinal mucosal damage and the maintenance of intestinal homeostasis in colitis. The coordination of KLF5 on IECs proliferation and migration is essential for the execution of repair program encoded by Trp metabolic inputs.

### PI3K/AKT cascade decodes GPR35 signaling into KLF5-dependent repair commands

**Fig. 3:**
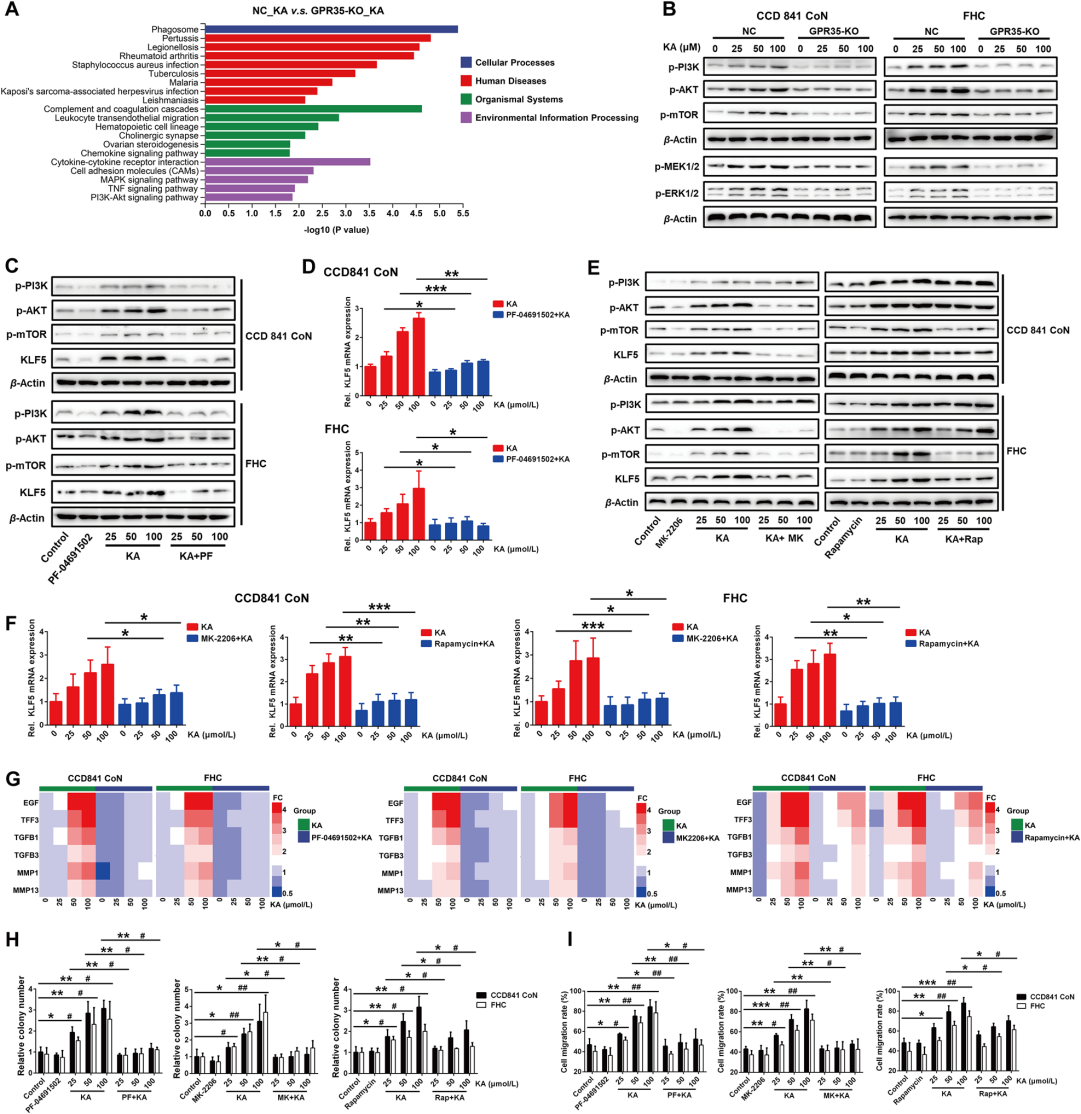
PI3K/AKT pathway mediates signal transduction between GPR35 and KLF5. **A** Pathway enrichment analysis of differentially expressed genes in KA treated GPR35 normal and deficient IECs based on KEGG database. **B** Western blot analysis of the activation of PI3K/AKT and MEK/ERK pathways in GPR35 normal and deficient IECs that were treated with KA for 48 h. **C, D** Effect of specific inhibition of PI3K phosphorylation using PF-04691502 (PF, 0.5 μmol/L) on KA-induced KLF5 protein (**C**) and mRNA (**D**) expression (*n* = 3). **E, F** Effect of specific inhibition of AKT and mTOR phosphorylation using MK-2206 (MK, 5 μmol/L) and Rapamycin (Rap, 0.5 μmol/L) respectively on KA-induced KLF5 protein (**E**) and mRNA (**F**) expression (*n* = 3). **G−I** Effect of specific inhibition of PI3K, AKT and mTOR phosphorylation respectively on KA-induced IECs proliferation (**H**), migration (**I**) and their related gene expression (**G**) (*n* = 3). Data are presented as means ± SD. Statistical analysis was performed using Student’s *t* test. *P < 0.05, **P < 0.01 and ***P < 0.001.

To further elucidate how GPR35 integrates intestinal mucosal damage-associated Trp metabolic signals into KLF5-driven repair programming, we interrogated the signaling nexus linking KA sensing to transcriptional reprogramming. KEGG pathway enrichment analysis based on RNA-Seq data indicated that the downstream signal regulation of GPR35-mediated KA sensing may be involved in PI3K/AKT and MAPK signaling pathways (Fig. 3A; Fig. S5A). Physiological studies indicate that GPRs perform a range of functions, including the activation of heterotrimeric G proteins, calcium mobilization, and β-arrestin-2 recruitment. They mediate signal transduction through second messengers such as cAMP, Ca²⁺, and DAG, and regulate cellular physiological functions via numerous signaling pathways including PI3K/AKT and MAPK/ERK. [19] Data from western blot analysis demonstrated significant activation of the PI3K/AKT and MEK/ERK signaling pathways by KA. Notably, the absence of GPR35 resulted in a complete abrogation of KA-induced activation of these two pathways (Fig. 3B). Subsequently, the specific inhibitor PF04691502 was employed to selectively inhibit PI3K activation, resulting in a reduction in AKT and mTOR protein phosphorylation in colon epithelial cells. This inhibition significantly hindered the promotional effect of KA on KLF5 protein and mRNA expression (Fig. 3C, D). However, the selective inhibition of the MEK/ERK signaling pathway did not significantly affect KLF5 protein expression induced by KA (Fig. S5B). This signaling selectivity establishes PI3K/AKT as the exclusive metabolic signal decoder in this GPR35-KLF5 circuitry. Subsequently, the phosphorylation of AKT and mTOR proteins downstream of this pathway was specifically inhibited. While MK-2206 and Rapamycin did not significantly impact the KA-induced upregulation of PI3K phosphorylation, they markedly suppressed the promotion of KLF5 protein and mRNA expression induced by KA (Fig. 3E, F). Meanwhile, the inhibition of PI3K, AKT, or mTOR protein phosphorylation significantly attenuated the promotional effect of KA on IECs proliferation and migration, as well as the expression of its associated genes, including EGF and TFF3 and so on (Fig. 3G–I).

**Fig. 4:**
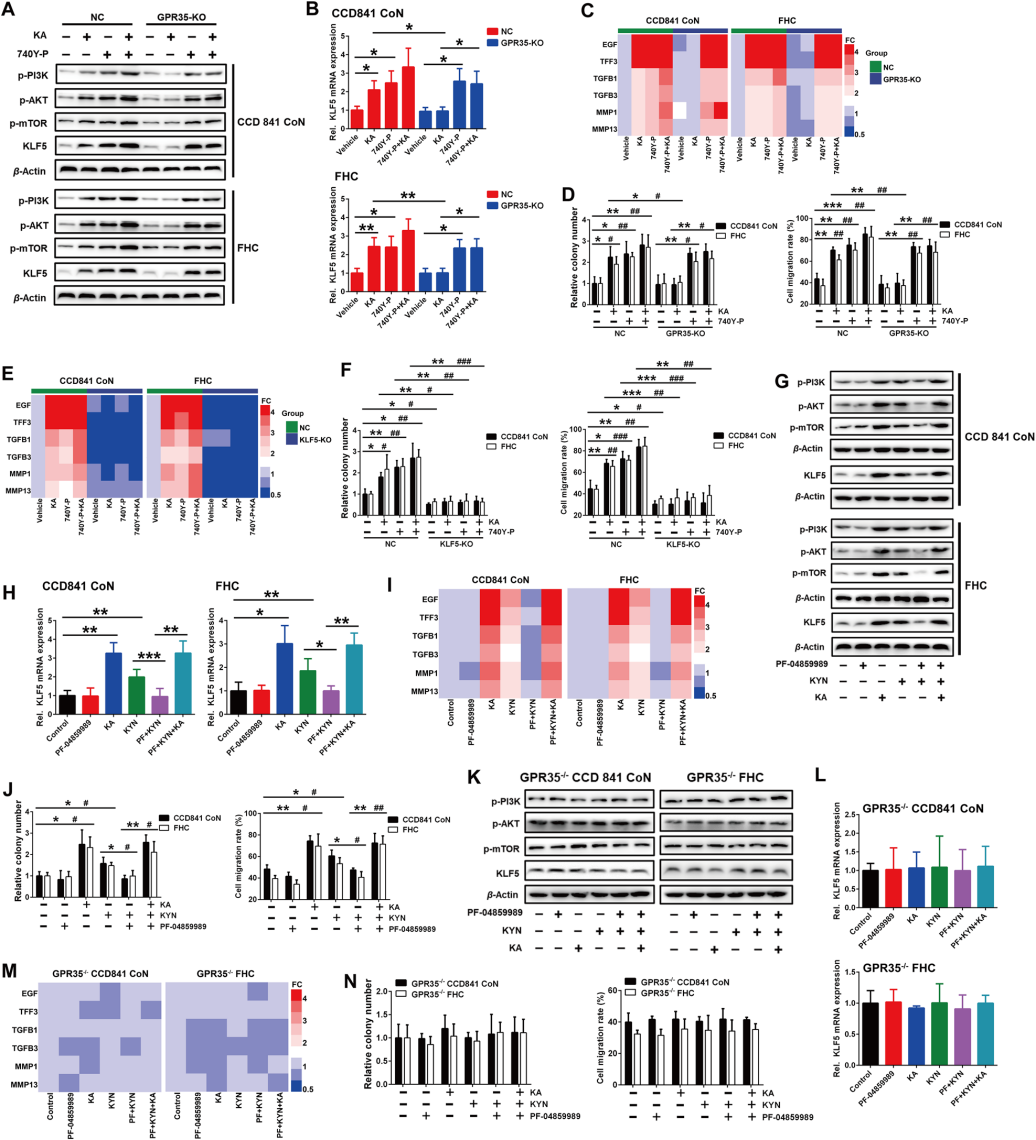
GPR35 regulates KLF5 by sensing abnormal Trp metabolism to drive IECs proliferation and migration. **A**, **B** Western blot and RT-PCR analyses of PI3K/AKT pathway activation, KLF5 protein (**A**) and mRNA (**B**) expression respectively in GPR35 normal and deficient IECs that were treated with KA or 740Y-P, a PI3K agonist, 10 μmol/L for 48 h (means ± SD, *P < 0.05, **P < 0.01, Student’s *t* test, *n* = 3). **C, D** RT-PCR, clone formation and wound healing analyses of the changes of GPR35 normal and deficient IECs proliferation, migration (**D**) and their related gene expression (**C**) respectively after treated with KA or 740Y-P for 48 h (means ± SD, * and # are represented statistically significance of the data from CCD841 CoN and FHC cells respectively, *^/#^*P* < 0.05, **^/##^*P* < 0.01, ***P < 0.001, Student’s *t* test, *n* = 3). **E**, **F** Changes of KLF5 normal and deficient IECs proliferation, migration (**F**) and their related gene expression (**E**) after treated with KA or 740Y-P for 48 h (means ± SD, *^/#^*P* < 0.05, **^/##^*P* < 0.01, ***^/###^*P* < 0.001, Student’s *t* test, *n* = 3). **G**, **H** and **K**, **L** Changes of PI3K/AKT pathway activation, KLF5 protein and mRNA expression in GPR35 normal (**G**, **H**) and deficient (**K**, **L**) IECs that were pretreated with (or without) PF-04859989 (PF, a KATⅡ inhibitor, 5 μmol/L) for 2 h prior to supplemented with KYN and KA treatment for 48 h (means ± SD, *P < 0.05, **P < 0.01, ***P < 0.001, Student’s *t* test, *n* = 3). **I, J** and **M**, **N** Changes of GPR35 normal (**I**, **J**) and deficient (**M**, **N**) IECs proliferation, migration and their related gene expression after pretreated with (or without) PF-04859989 for 2 h prior to supplemented with KYN and KA treatment for 48 h (means ± SD, *^/#^*P* < 0.05, **^/##^*P* < 0.01, ***^/###^*P* < 0.001, Student’s *t* test, *n* = 3).

Then, a specific agonist of the PI3K/AKT cascade was used to further investigate. The results showed that, akin to the function of KA, 740Y-P significantly upregulated the expression of KLF5 protein and mRNA (Fig. 4A, B). Moreover, it effectively promoted ECs proliferation and migration along with the upregulation of EGF, TFF3 and other related genes (Fig. 4C, D). Although deleting GPR35 gene could obstruct the effect of KA, it failed to block the promotion effect of 740Y-P on the expression of KLF5 and its mediated genes and the proliferation and migration of IECs. The knockout of KLF5 did not significantly influence the activation of PI3K/AKT cascade induced by KA and 740Y-P. However, it abrogated the stimulatory effects of them on IECs proliferation and migration, as well as the expression of six related genes including EGF, TFF3 and so on. (Fig. 4E, F; Fig. S6A). In addition, we overexpressed KLF5 in IECs that PI3K/AKT cascade inhibited, the low KLF5 expression caused by PI3K inhibition was restored, and the inhibition of PF-04691502 on IECs proliferation and migration regulated by GPR35-mediated KA sensing was reversed (Fig. S6B–D). These findings suggest that PI3K-AKT-mTOR cascade functions as a digital converter in the GPR35-KLF5 circuitry, selectively transducing KA flux into KLF5-dependent repair programming that driving IECs proliferation and migration through orchestrating transcriptional reprogramming.

### GPR35 drives IECs proliferation and migration by coupling Trp metabolic flux to KLF5 outputs

The sensing of signals indicative of intestinal mucosal damage by IECs and the subsequent driving of cell proliferation and migration constitute the initial phase of IEC involvement in the repair process of intestinal mucosal damage in UC, determining the onset of injury repair. Hence, we modulated Trp-KYN-KA axis metabolic flux to further investigate the triggering and driving effect of GPR35 on IECs proliferation and migration [16]. The results demonstrated that, in wild-type IECs, KYN significantly activated PI3K/AKT cascade and promoted KLF5 protein and mRNA expression. Conversely, inhibiting the metabolism of KYN to KA by using PF-04859989, a KATⅡ specific inhibitor, significantly blocked the activation of PI3K/AKT cascade and the promotion of KLF5 expression that are induced by KYN (Fig. 4G, H). Supplementing KA in the PF-04859989 + KYN group obviously reversed the suppression of PI3K/AKT cascade activation and KLF5 expression induced by PF-04859989. Consistently, the significant promotions of KYN on IECs proliferation and migration and their related genes, including EGF, TFF3, and so on, were also observed, while these effects were blocked upon inhibition of the metabolism from KYN to KA. It is worth noting that the addition of KA in the PF-04859989 + KYN group significantly reversed the inhibitory effects of PF-04859989 on IECs proliferation and migration, as well as the expression of the related genes, and restoring the levels to comparable with those observed in the KA treatment group (Fig. 4I, J). However, in GPR35 deleted IECs, there appears an obvious defect of IECs in Trp metabolism sensing. KA and KYN stimulations, as well as the intervention of Trp-KYN-KA axis metabolism, all did not elicit significant changes in PI3K/AKT cascade activation, KLF5 expression, and IECs proliferation, migration and their related gene expression (Fig. 4K–N). Additionally, KLF5 gene deficiency does not affect the sensing of abnormal Trp metabolism and the following signal transduction (Fig. S6A). Whereas, the downstream signal response, that is, the triggering and driving of IECs proliferation and migration and its related gene expression, has been seriously hindered (Fig. S6E, F). These results indicate that the triggering and driving of IECs proliferation and migration rely on coupling the information of Trp metabolic flux to KLF5-dependent outputs via GPR35.

### The GPR35-KA “Sandwich” interface ensures specific metabolic surveillance in damage decoding

**Fig. 5:**
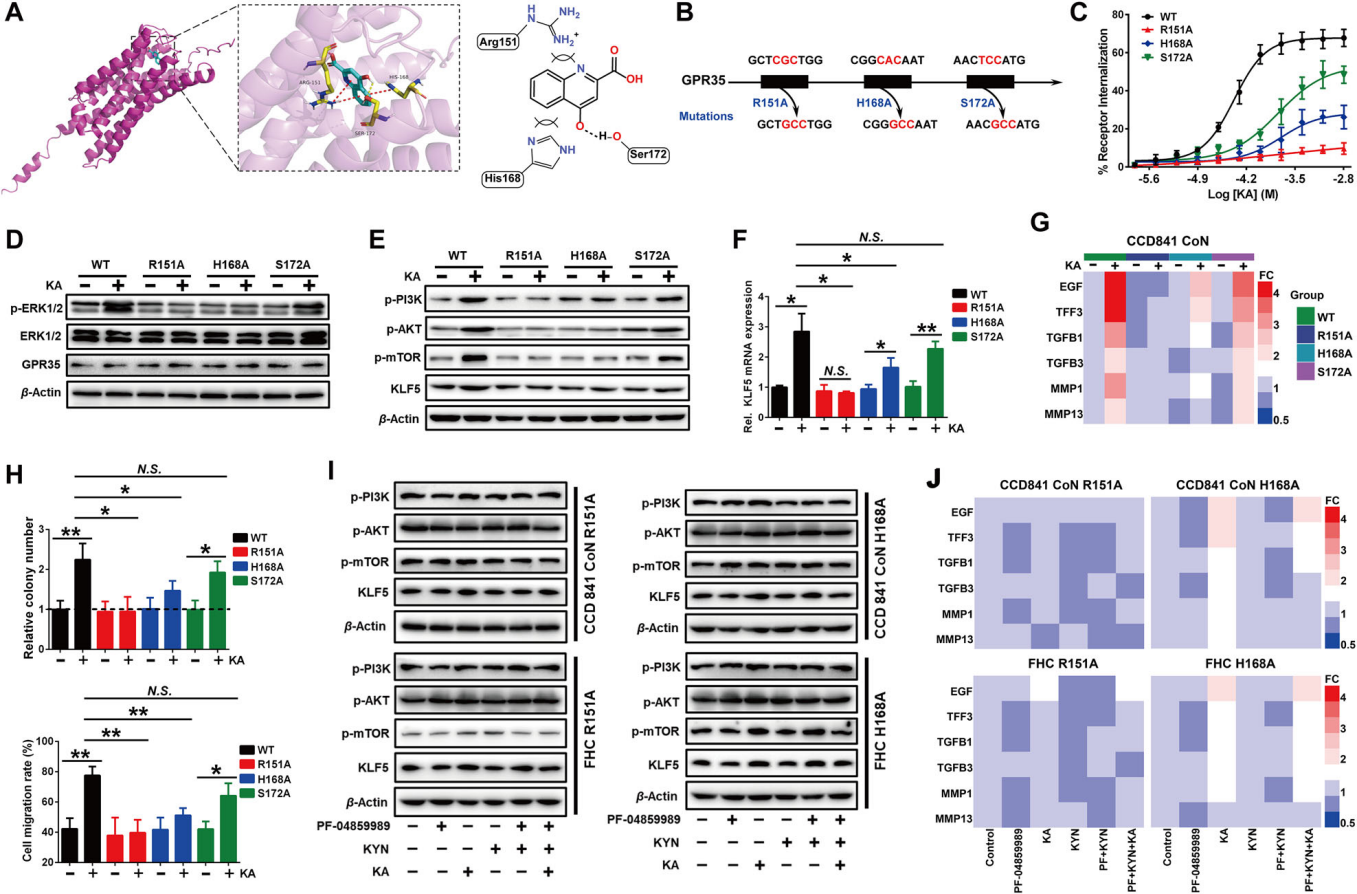
The key interaction mode of GPR35 monitoring Trp metabolism. **A** The binding modes of GPR35 and KA. **B** GPR35 mutation manner. **C** Flow cytometry analysis of mutated-GPR35 internalization after KA treatment for 24 h (means ± SD, *n* = 3). **D**−**F** Western blot and RT-PCR analyses of the effects of KA on ERK1/2 phosphorylation (**D**), PI3K/AKT pathway activation, KLF5 protein (**E**) and mRNA (**F**) expression in GPR35 mutated IECs (means ± SD, *P < 0.05, Student’s *t* test, *n* = 3). **G**, **H** RT-PCR, clone formation and wound healing analyses of the effects of KA on GPR35 mutated IECs proliferation, migration (**H**) and their related gene expression (**G**), respectively (means ± SD, *P < 0.05, **P < 0.01, Student’s *t* test, *n* = 3). **I**, **J** Changes of PI3K/AKT pathway activation, KLF5 protein (**I**) and its mediated proliferation and migration related gene (**J**) expression in GPR35 mutated IECs that were pretreated with (or without) PF-04859989 for 2 h prior to supplemented with KYN and KA treatment for 48 h (*n* = 3).

There are evidences suggesting specific residues within GPR35 transmembrane domains III and IV are associated with ligand binding, particularly arginine residues located in R151 [20]. To further elucidate the stereospecific ligand recognition hub of GPR35 coordinating Trp metabolic abnormalities sensing and signal decoding, we constructed the potential active domain around Arg (R)151 in transmembrane domains III, IV and its surrounding domains V and VI based on the GPR35 protein structure predicted by AlphaFold. Molecular docking data displayed that the optimal bonding conformation of KA and GPR35 involves three key interactions: the nitrogen atom of guanidyl in R151 residue side chain forms a cation-π bond with the quinoline ring of KA, the imidazole ring of His (H)168 residue side chain forms a π-π stacking effect with KA’s benzene ring, and the hydroxyl oxygen atom of Ser (S)172 residue forms a hydrogen bond with KA’s phenol hydroxyl hydrogen (Fig. 5A). This conformation, R151-KA-H168 constitutes a metabolic gatekeeping “sandwich” interface with the lowest binding free energy.

Then, we created GPR35 mutants to destroy this unique “sandwich” structural interaction, in which R151, H168 and H172 residues were converted to alanine, respectively (Fig. 5B). Receptor internalization is an important pathway of activated GPRs to transfer signals, through which the downstream effector molecule ERK1/2 is phosphorylated [21]. This is frequently employed to characterize the activation and response of GPR35 [22]. The results of the GPR35 receptor internalization analysis demonstrated that the activation level of GPR35 was varying affected after the three sites are mutated, respectively. Among them, the R151A mutation resulted in the abrogation of sensitivity of GPR35 to KA perception, and the H168A mutation also significantly affected the sensing sensitivity of GPR35 to KA, while the S172A mutation exhibited a comparatively minor impact on the sensing of GPR35 to KA (Fig. 5C). Moreover, R151A and H168A mutations markedly attenuated the promotional effect of KA on ERK1/2 phosphorylation, while the S172A mutation exhibited a comparatively minor impact on KA-induced ERK1/2 phosphorylation (Fig. 5D; Fig. S7A). These suggest that the positively charged alkaline amino acid residues R151 and H168 are the key sites for GPR35 sense abnormal Trp metabolism and signal transduction. The “sandwich” structural interaction between R151, H168 residues and KA through cation-π bond and π-π stacking is the key mode of action by which GPR35 monitors Trp metabolic abnormalities, while the S172 site is not critical in this process. This architecture achieves selective KA recognition over upstream Trp metabolites through charge complementarity and spatial constraints.

Indeed, GPR35 R151A and H168A mutations significantly weakened the activation of PI3K/AKT signaling cascade and the promotion of KLF5 expression induced by KA. (Fig. 5E, F; Fig. S7B, C). Correspondingly, the abilities of KA to promote the proliferation and migration of IECs as well as the related gene expression were also significantly reduced following R151A and H168A mutations, while S172A mutation had little impact on it (Fig. 5G, H; Fig. S7D, E). What’s more, the GPR35 R151A and H168A mutations produced biological effects similar to those observed in GPR35-KO IECs. Inhibition of the IECs Trp-KYN-KA axis metabolism or supplementation of KYN and KA in the growth environment did not induce significant changes in downstream effects, including PI3K/AKT cascade activation and the expression of KLF5 and its mediated genes involved in cell proliferation and migration (Fig. 5I, J; Fig. S7F). IECs with GPR35 R151A or H168A mutations lost the ability to monitor Trp metabolism abnormalities and orchestrate downstream signal transduction, highlighting the irreplaceability of R151 and H168 sites in GPR35-dependent damage signals discrimination and decoding.

### GPR35-mediated abnormal Trp metabolism sensing drives intestinal mucosal repair through KLF5

**Fig. 6:**
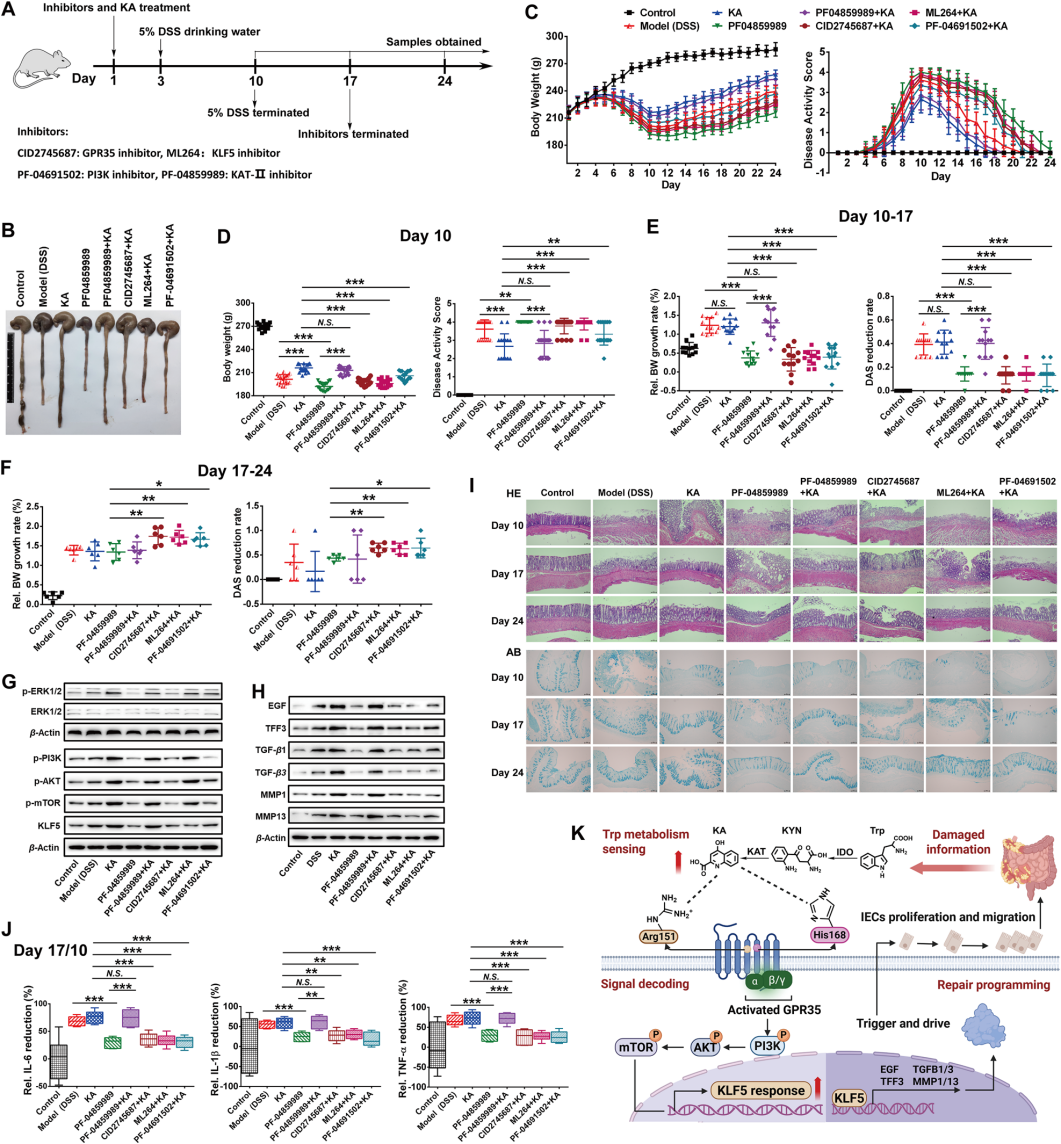
GPR35-mediated abnormal Trp metabolism sensing drives the repair of damaged-gut mucosa through KLF5. **A** Schematic diagram of animal disposition process. **B** Representative images of rat colon tissues on day 10. **C** The changes of body weight (left panel) and DAS (right panel) of each group rats were monitored twice daily (*n* = 6–18). **D** Rat body weight and DAS of each group on day 10 (*n* = 18). **E**, **F** The relative body weight growth rate and DAS reduction rate during the period of day 10–17 (**E**) and 17–24 (**F**) were analyzed to characterize the change speed of rat body weight and DAS (*n* = 6–12). **G**, **H** Western blot analysis of ERK1/2 phosphorylation, PI3K/AKT pathway activation, KLF5 (**G**) and its mediated proliferation and migration related proteins (**H**) expression in the colon after rat administration on day 10. **I** HE and AB staining analyses of rat colon tissues, scale bars = 100 μm. **J** The relative variational amplitudes of inflammatory factor level in colon during the period of day 10–17 were analyzed to reflect the recovery speed of inflammation (*n* = 6). **K** The schematic form of proposed mechanism that GPR35 initiate and regulates damaged-gut mucosal repair through monitoring Trp metabolism (Created with BioRender.com, Agreement number: JO28T59X5V). Data are presented as means ± SD. Statistical analysis was performed using Student’s *t* test. *P < 0.05, **P < 0.01 and ***P < 0.001.

To explore the dynamic decoding process of the GPR35-KLF5 circuitry in intestinal mucosal repair programming, we implemented phased interventions in DSS-induced colitis models (Fig. 6A), in which the selected inhibitor intervention dose did not affect intestinal homeostasis of rats (Fig. S8). During the DSS stimulation period (Day 3-10), it was observed that each group rats, except for control group, displayed varying degrees of mental fatigue, lethargy, bradykinesia, hair tarnish and disheveled, and accompanied with significantly decreased food and water intake. These conditions progressively deteriorated with the experiment progressed. Obviously, compared to the model group and other inhibitor intervention groups (PF-04859989, CID2745687 + KA, ML264 + KA and PF-04691502 + KA), both KA and PF-04859989 + KA treatment groups exhibited significant improvements in disease states. Upon cessation of DSS stimulation (Day 10–17), the disease states of model, KA and PF-0-489599 + KA group rats demonstrated rapid amelioration. Although the disease status of rats in KAT-Ⅱ, GPR35, KLF5, and PI3K inhibition groups shown varying degrees of improvement during the period, they still exhibited more severe pathosis. Until the inhibitor interventions were terminated (Day 17–24), the disease state of these group rats appeared rapid improvement. Besides, at the end of DSS stimulation (Day 10), the colonic tissue of model and inhibitor intervention groups (PF-04859989, CID2745687 + KA, ML264 + KA and PF-04691502 + KA) displayed severe pathological conditions, including pronounced swelling, bleeding, watery intestinal content and shorten in length. However, the colonic conditions of KA and PF-04859989 + KA intervention group rats were significantly superior to those observed in the aforementioned groups (Fig. 6B).

Through daily monitoring, it was observed that at the end of DSS treatment (Day 10), KA treatment significantly slowed down rat weight loss and DAS increase compared to the model group. Inhibition of the Trp-KYN-KA axis metabolism (PF-04859989 group) markedly exacerbated weight loss and DAS elevation in rats, while supplementation with KA (PF-04859989 + KA group) dramatically mitigated the rats’ weight loss and DAS increase compared with PF-04859989 group (Fig. 6C, D). Simultaneously, the administrations of GPR35 inhibitor (CID2745687), KLF5 inhibitor (ML264) and PI3K inhibitor (PF-04691502) significantly blocked the inhibitory effects of KA on the rat weight loss and DAS increase (Fig. 6C, D). Despite observing varying degrees of improvement in body weight and DAS among the different group rats during the 7 days following DSS stimulation (Day 10–17) (Fig. 6C; Fig. S9A), the rate of change analysis revealed that inhibition of Trp-KYN-KA axis metabolism significantly delayed the recovery of the rat body weight and DAS compared to model group (Fig. 6E). Meanwhile, persistent inhibition of GPR35, KLF5 and PI3K also markedly hindered the facilitative effects of KA on body weight and DAS recovery in rats (Fig. 6E). During the 7 days following inhibitor intervention terminated (Day 17–24), the body weight and DAS recovery rates in CID2745687 + KA, ML264 + KA and PF-04691502 + KA groups showed significantly faster than those in PF-04859989 group (Fig. 6F). This may be attributed to the sustained KA intervention in these three groups. In addition, after the GPR35, KLF5, PI3K, and Trp-KYN-KA axis metabolic inhibitor interventions terminated (Day 17–24), the recovery rates of body weight and DAS in rats were significantly accelerated compared to the before (Day 10–17) (Fig. S9B).

To further elucidate the impact of intrinsic regulation driven by the sensing of abnormal Trp metabolism on the development and pathological phenotype of colitis, the protein expression and histopathological alterations in colonic tissues were analyzed. Following the occurrence of colitis (Day 10), there was an observed increase in the phosphorylation of ERK1/2 within colon tissue, indicating activation of GPR35 (Fig. 6G), which may be attributed to the increase of Trp-KYN-KA axis metabolism following the intestinal damage [16]. This also partially explained the reason of the activation of PI3K/AKT signaling cascade, as well as the up-regulated expression of KLF5 and its mediated cell proliferation and migration associated proteins in the model group (Fig. 6G, H). Indeed, when a KAT-Ⅱ specific inhibitor was applied to suppress Trp-KYN-KA axis metabolism (PF-04859989 group), ERK1/2 phosphorylation, PI3K/AKT cascade activation, and KLF5 and its mediated proliferation and migration related protein expression in the colon were significantly decreased compared with the model group. Whereas, supplemented with KA treatment in the KAT-Ⅱ inhibited group (PF-04859989 + KA group), their levels significantly increased to match those of the KA group (Fig. 6G, H). These results further corroborate our hypothesis. Although GPR35 can drive the repair of intestinal mucosal damage through sensing KA, the persistent stimulation of DSS results in the damage progress of the colon to exceed the repair process, thereby leading to a progressive exacerbation of colon damage. Nevertheless, administration of exogenous KA (KA group) inevitably further elevates KA flux in the colon, resulting in a significant increase in ERK1/2 phosphorylation, PI3K/AKT cascade activation, and upregulation of KLF5 and its related proteins involved in proliferation and migration compared to the model group (Fig. 6G, H). This alleviates intestinal mucosal damage and inflammatory reaction caused by DSS stimulation to a certain extent, including the mitigation of extensive intestinal mucosal erosion, epithelial necrosis, crypts disappearance, goblet cells decrease, mucus barrier disruption, intestinal permeability increase, and inflammatory factors elevation (Fig. 6I; Fig. S9C, D). Furthermore, the constant stream of KA is continuously sensed by GPR35, which is characterized by high expression of ERK1/2, leading to PI3K/AKT signaling cascade activation and KLF5 along with its mediated proteins expression involved in proliferation and migration maintaining a higher level, consistently. Once the stimulus DSS is eliminated (Day10–17), rapid repair of the intestinal mucosal damage is achieved (Fig. 6J; Fig. S9E). Conversely, inhibiting Trp-KYN-KA axis metabolism results in a reduction in GPR35 sensing levels and damage signal intensity, causing the body make a wrong judgment on the severity of intestinal mucosal damage. This ultimately led to a low level of the body’s drive effect on the damage repair, the intestinal mucosal damage was further aggravated. Obviously, the serious pathological injury and inflammatory reaction were still exhibited in Trp-KYN-KA axis metabolism inhibited group after DSS stimulation was terminated 7 days (Day 17) (Fig. 6I; Fig. S9C, D).

Simultaneously, during the inhibitor intervention (Day 1–17), CID2745687 inhibited the sensing of KA by GPR35, causing the signaling of KA level abnormality transduced by the PI3K/AKT cascade and the following KLF5 response was significantly attenuated. The intervention of PF-04691502 effectively blocked the regulation of KLF5 by GPR35-mediated KA sensing via PI3K/AKT cascade in the colon. ML264 had no significant effect on GPR35-mediated KA sensing and activation of PI3K/AKT signaling cascade, but significantly inhibited the promotional effect of KA on KLF5 protein expression (Fig. 6G; Fig. S9F). The inhibition of KLF5 expression, GPR35 and PI3K/AKT signaling cascade activations respectively, all resulted in the abnormities of the response of colonic mucosa to damage signals (i.e., abnormal Trp metabolism) and the initiation of repair after damage. These reflected as the significant reduction in the expression of genes related to proliferation and migration induced by KA (Fig. 6H; Fig. S9G), and further led to significant suppression of KA’s ability in alleviating colitis-induced intestinal mucosal damage (Day 10) (Fig. 6I). At the same time, compared with the rapid repair of the damaged intestinal mucosa in KA treatment group after the elimination of DSS stimulation (Day 17), the repair speed in GPR35, PI3K/AKT cascade, and KLF5 protein inhibition groups were significantly attenuated, and still exhibited evident histopathological alterations and inflammatory reaction (Fig. 6I; Fig. S9C, D). Additionally, during the inhibitor terminated for 7 days (Day 24), the GPR35-mediated KA sensing and signal regulation in the colon of the CID2745687 + KA, ML264 + KA and PF-04691502 + KA group rats were restored due to the continuous administration of KA. The expression of KLF5 and its mediated proteins associated with cell proliferation and migration were restored to a comparable level with that observed in the KA intervention group (Fig. S9F, G), and the rapid repair of the damaged intestinal mucosa were achieved in the three groups (Fig. 6I). Together, these results establish that the GPR35-KLF5 circuitry functions as a metabolic gatekeeping that surveille Trp catabolite flux, decodes intestinal mucosal damage intensity through PI3K/AKT cascade, and programs epithelial repair via KLF5-mediated transcriptional rewiring (Fig. 6K). The abnormality of any node in this intricate network will significantly impact the repair process of damaged intestinal mucosa and cause more serious tissue damage.

## Discussion

Cellular and tissue homeostasis is a key physiological variable and is maintained by the interaction of metabolites and their monitor [10, 13]. Understanding these systems is imperative to minimize health problems and has important implications for decoding and intervening the occurrence and development of UC, which remains an unexplained and refractory disease worldwide [1]. Although the current intervention of UC is still mainly focused on symptom relief, promoting the repair of intestinal mucosal damage and maintaining the integrity of intestinal barrier have always been the ideal goals of clinical pursuit of UC therapy [23, 24]. Physiological studies have indicated that IECs is a core component involved in the repair of intestinal mucosal damage, and it proliferate and migrate to the damaged lesions is an important physiological basis for the repair process [6, 7]. However, how IECs sense intestinal mucosal damage and how to initiate and drive repair program after sensing, which is involved in the initial stage of IECs participating in damaged intestinal mucosa repair, remains pendent.

In this work, we propose that abnormality in the Trp-KYN-KA axis metabolism serves as a key messenger in the occurrence of intestinal mucosal damage of UC. Importantly, this messenger is captured by GPR35 through a unique “sandwich” structural interaction of R151-KA-H168, which drives IECs proliferation and migration, initiating the repair program of intestinal mucosal damage. Actually, Different ligands exhibit a distinct preference for modulating the downstream signal and functions of their monitors, which is closely related to the structure of ligands and the amino acid residues that recognize it [25]. In the perceptual pattern proposed in this study, both R151 and H168 are positively charged alkaline amino acid residues that form cation-π bond and π-π stacking interactions with the quinoline ring and benzene ring of KA, respectively. This is the key mode for GPR35 to sense abnormal Trp metabolism and carry out signal decoding. This interaction allows KA and GPR35 to maintain a relatively fixed angle and stable distance. Disrupting this “sandwich” binding mode through mutations result in the defect of GPR35’s ability to sense abnormal Trp metabolism and drive IECs proliferation and migration. Our findings provide a structural basis for GPR35 to sense various organic acid with quinoline parent nuclei. Interestingly, mutual supporting evidence with us has been proposed that 5-HIAA derived from platelets and mast cells, which shares a similar molecular structure with KA and is also identified as a ligand for GPR35, induces neutrophil transepithelial migration to inflammatory lesions through GPR35, mediating rapid clearance of invading bacteria [26]. Although our study does not exclude the possibility that other sources of GPR35^+^ cells may migrate to colonic tissue and affect damaged intestinal mucosa repair, our findings provide strong evidence for the first time that IECs sense intestinal mucosal damage and encode this message into the repair program through GPR35-mediated abnormal Trp metabolism sensing.

More importantly, we identified KLF5 as a key effector of GPR35 in this study. Their mutual crosstalk establishes a Trp metabolic gatekeeping system in the intestinal mucosal damage and repair. Thereinto, GPR35 drives IECs proliferation and migration by coupling Trp metabolic flux to KLF5 outputs. KLF5 was originally known as colon Kruppel-like factor or intestinal enriched Kruppel-like factor. Current researches showed that KLF5 plays an extremely important role in various physiological and pathological processes, including tissue remodeling, angiogenesis, intestinal tissue homeostasis, intestinal villi formation, the maturation and maintenance of intestinal crypts, and the proliferation and survival of intestinal epithelial stem cells [27–29]. Defects in the KLF5 gene can lead to disordered structures in the mouse intestinal epithelial tissue, intestinal inflammation, and increased gut permeability. Conversely, overexpression of KLF5 in intestinal tissue can significantly reduce tissue damage caused by various stimuli [30]. Additionally, KLF5 can mediate external stress responses following tissue damage. Ex vivo vascular experiments showed that KLF5 expression was induced in response to myocardial tissue and vascular damages, thereby contributing to the activation and proliferation of smooth muscle cells and fibroblasts [31]. Significant increases in KLF5 responses also have been observed following intestinal damage under exposure to LPS and bacteria, radiation-induced renal and rectal vascular damages in mice, and so on [32]. These studies collectively imply that KLF5 may play a crucial role in the cellular responses to intestinal mucosal damage [30]. However, regrettably, its underlying regulatory mechanism has not been elucidated. In this study, we found that KLF5 expression is significantly upregulated in the colonic tissue of UC patients, and inhibition of its levels lead to further exacerbation of intestinal mucosal damage and a significant decrease in the rate of repair. Furthermore, KLF5 can respond to GPR35’s sensing of Trp metabolic abnormalities through the PI3K-AKT-mTOR mediated signaling cascade, upregulate the expression of genes related to IECs proliferation and migration, including EGF, TFF3, TGFB1/3, MMP1/13, and drive the repair of damaged intestinal mucosa. Our findings unveil part of the mysteries surrounding the sensing and response to intestinal mucosal damage signals, as well as the triggering and driving of the repair process.

In fact, EGF, TFF3, TGF-β1/3, and MMP1/13 are critical factors in tissue repair, playing essential roles in epithelial cell proliferation and migration, tissue homeostasis maintenance and organoid formation, as has been widely described [33–36]. Notably, their expression is significantly upregulated following tissue damage, which is consistent with our observations in clinical samples from patients with UC. However, the regulatory relationship and mechanisms linking their expression changes to the occurrence and development of UC remain enigmatic. Our research reveals that during the repair of intestinal mucosal damage, the expression of cell proliferation and migration-related genes such as EGF, TFF3, etc. is induced by KLF5 and tightly regulated by GPR35-mediated KA sensing. In the GPR35-KLF5 circuitry, GPR35 functions like a “Gatekeeper”, performing the specific tryptophan perturbations input and controlling the amount and direction of signal flow, while KLF5 responds to these signals and determines the initiation and driving of IECs proliferation and migration by orchestrating transcriptional reprogramming of relevant genes. In this process, PI3K-AKT-mTOR phosphorylation cascade functions as a signal converter and plays the role of selectively decoding KA flux into KLF5-dependent repair commands. This may be attributed to the concerted action of stereospecific KA recognition by GPR35 R151/H168 residues and mTOR-mediated modulation of KLF5 histone acetylation [37]. This tight and finely tuned signaling cascade circuitry enables the body to initiate a repair defense response in facing the challenge of various factors causing intestinal mucosal damage, thereby quickly counteracting damage progression. Any abnormality at any of these nodes will significantly impact the repair process, leading to more severe intestinal mucosal damage. Indeed, inhibiting the Trp-KYN-KA axis metabolism reduces the level and intensity of GPR35’s perception of injury signals, and causes a wrong judgment made by IECs on the severity of intestinal mucosal damage. Inhibition of GPR35 sensing, PI3K/AKT pathway signal transduction, or KLF5 expression all result in IECs being indifferent to the damage challenge and losing the action capability of damage repair. These evidences emphasize the importance of GPR35-mediated abnormal Trp metabolism sensing regulation of KLF5 in initiating and driving damaged gut mucosal repair.

In addition, these results also provide some important implications. Firstly, multi-center, large-cohort clinical studies are needed to further validate the correlation between Trp-KYN-KA axis perturbations, the GPR35-KLF5 circuitry trigger, and UC disease activity. Such studies could lay the groundwork for developing non-invasive diagnostic biomarkers and prognostic tools to predict repair capacity in UC patients. Secondly, lineage tracing targeting GPR35 or KLF5 in specific cell types (e.g., IECs, neutrophils, or intestinal stem cells) and mapping GPR35-KLF5 circuitry in the context of the intact intestinal microenvironment during different stages of UC progression (active vs. remission) and repair (early vs. late phase) would further clarify the cell-specific roles and multicellular crosstalk orchestrated by this circuitry during mucosal epithelial repair. Finally, therapeutic targeting of the GPR35-KLF5 circuitry holds promise for accelerating mucosal healing in patients refractory to conventional anti-inflammatory therapies, by enhancing IECs proliferation and migration. Concomitantly, designing GPR35 agonists with high specificity for the KA-binding “sandwich” pocket (R151/H168 residues) via structural biology and medicinal chemistry could help mitigate off-target effects and poor efficacy of GPR35 ligands.

In summary, our study reveals a metabolic gatekeeping mechanism in epithelial repair that highlights both the initiative of IECs in repairing behavior in response to the challenge of intestinal mucosal damage, and the importance of the cross-talk between metabolites and their monitors in maintaining tissue homeostasis. We demonstrate that GPR35 monitors the occurrence of intestinal mucosal damage by sensing perturbations in Trp metabolism, and decodes the damage signals into KLF5-dependent repair program via the PI3K-AKT-mTOR cascade. This circuitry licenses KLF5 to orchestrate transcriptional reprogramming to trigger and drive IECs proliferation and migration. Our findings not only provide new insights into the sensing of intestinal mucosal damage and the triggering and driving of repair, but also offer important implications for the prevention and treatment of UC by targeting mucosal repair.

## Materials and methods

### Collection of clinical samples

Ten pairs of human UC lesions and adjacent normal colonic tissue were obtained from patients diagnosed with UC and received colonoscopy at the People’s Hospital of Longhua, Shenzhen. No patient had received conventional therapy. The procedure of this study was approved by the Ethics Committee of the People’s Hospital of Longhua, Shenzhen (approval number: Ethical review of the People’s Hospital of Longhua (Institute) [2024] No. 029), and the utilization of clinical samples followed the guidelines of the Ethics Committee of the hospital. Informed consent was obtained from all patients for the use of all human tissue and cells used in this study.

### Cell culture

Human normal colonic epithelial CCD 841 CoN cell line (RRID: CVCL_2871) and FHC cell line (RRID: CVCL_3688) were obtained from American Type Culture Collection (ATCC, Manassas, VA, USA), and identified at Guangzhou jenniobio Biotechnology Co., Ltd (Guangzhou, China) and Genetic Testing Biotechnology Co., Ltd (Suzhou, China), respectively, using Short Tandem Repeat (STR) analysis. CCD 841 CoN cell line was cultured in Eagle’s Minimum Essential Medium (Gibco, Grand Island, NY, USA) supplemented with 10% fetal bovine serum (FBS) (Gibco), 1% penicillin/streptomycin (Gibco) and 1 × Non-essential amino acids (NEAA) (Gibco). FHC cell line was cultured in DMEM/F12 medium (Gibco) supplemented with 10% FBS, 10 mM HEPES (Gibco), 10 ng/mL cholera toxin (MedChemExpress, Shanghai, China), 1 × Insulin-Transferrin-Selenium Solution (Gibco), 100 ng/mL hydrocortisone (Sigma, St. Louis, MO, USA) and 1% penicillin/streptomycin. All cells were maintained at 37 °C in a humidified atmosphere containing 5% CO_2_.

### Construction of stable cell-lines with gene defects

GPR35 and KLF5 gene defected IECs were constructed using the CRISPR/Cas9 method. CCD 841 CoN and FHC cells were seeded into 6-well plates, and transfected with LV-EGFP-GPR35-gRNA or LV-EGFP-KLF5-gRNA lentivirus (Shanghai GenePharma Co., Ltd, Shanghai, China) and LV-Puro-Cas9 lentivirus (Shanghai GenePharma Co., Ltd) at a ratio of 1:1 after cells were achieved 30–50% confluent, according to the manufacturer’s instructions. The gRNA oligonucleotide sequences are listed in Table S1. 48 h after infection, the media were replaced with fresh culture media containing 2 µg/mL puromycin (Sigma, St. Louis, MO, USA), and continued culture for 7 days. The cells were digested and inoculated on 96-well plates (Corning Inc., Corning, NY, USA) after extreme dilution. Cells with monoclonal growth holes were selected and transferred to 24-well plates (Corning Inc.) for expanded culture. The knockout extent of the target gene was analyzed by western blot. The cells with the most thorough gene knockout were selected for follow-up experiments.

### Transcription factor binding sites analysis

The transcription factor binding sites of the gene promoter region were analyzed based on Eukaryotic Promoter Database (EPD) (https://epd.expasy.org/epd/). In the site of Search Motif Tool, the organism was selected as Homo sapiens, Library was selected as Transcription Factor Motifs (JASPAR CORE 2018 vertebrates), gene promoter region was set to from −1000 to 100 bp relative to TSS and a cut-off (P-value) was set as 0.001. The number of transcription factor binding sites was recorded and Heatmap Illustrator software was used for visualization.

### Gene overexpression and point mutation

The human KLF5 gene overexpression plasmid pcDNA3.1( + )-GFP-KLF5, which contained the full-length KLF5 gene (GenBank Accession number 688, NCBI), as well as GPR35 wild-type and point mutation plasmids (WT, R151A, H168A, S172A) were constructed by Shanghai GenePharma Co., Ltd. (Shanghai, China). The oligonucleotide sequences of the wild-type and mutated GPR35 gene were embedded in pcDNA3.1( + )-GFP vector plasmid. The KLF5 overexpression plasmid, GPR35 wild-type and mutated plasmids were transfected in GPR35 normal expressed or gene deleted CCD841 CoN and FHC cells, respectively, using Lipofectamine 2000 reagent (Invitrogen, Carlsbad, CA, USA) according to the manufacturer’s instructions.

### Clonogenic assays

Wild type or gene deletion type colonic epithelial CCD841 CoN and FHC cells were digested by trypsin and dispersed into single cells. 1000 cells, respectively, per well were evenly seeded in a 6-well plate (Corning Inc.). When the cells start to divide and proliferation, replace the previous culture medium with drug or plasmid-containing medium. Continue to culture cells for approximately 2 weeks. When visible clones appear in the wells, terminate the culture. Cells were washed twice with PBS carefully, fixed with 4.0% paraformaldehyde for 30 min, and stained with 0.5% crystal violet at room temperature for 30 min, successively. After cleaning and drying, the clonal cluster was photographed, and the number of colonies was quantified using ImageJ software (NIH, Bethesda, MD, USA). The ratio of the number of colonies in the treatment group to that in the control group is denoted as the relative number of colonies.

### Intestinal epithelial cell migration assays

Colonic epithelial cells (3 × 10^5^ cells/well) were seeded in a 24-well plate, and cultured until the cells reached 90−100% confluent. A straight scarification across the cell’s monolayer was scratched using a sterile 20 μL pipette tip, and then PBS washed to remove the damaged cells. The images of migrating epithelial monolayers at the same spot were captured using an inverted microscope (Leica, Shanghai, China) after drug treatment or plasmid transfection for 0 h and 48 h. The cell migration rate was calculated according to the following equation: Cell migration rate = (Wound area _0 h_ – Wound area _48 h_)/Wound Area _0 h_ × 100%.

### Receptor internalization analysis

The quantification of receptor internalization was performed by measuring specific antibody-tagged receptor on cells surface using flow cytometry. CCD841 CoN and FHC cells were seeded into 6-well plates (5 × 10^5^ cells/well) and treated by the concentration gradient of KA and vehicle for 24 h. Cells were collected and washed with ice-cold PBS, and then incubated with CoraLite® Plus 647-conjugated GPR35 Polyclonal antibody (1:500) (Proteintech, Chicago, IL, USA) at 4 °C for 1 h. The negative control was incubated with 3% BSA (Bovine serum albumin)/PBS buffer. After washed with ice-cold PBS, cells were resuspended with 4% paraformaldehyde and incubated at dark room temperature for 20 min. Cells were resuspended with ice-cold PBS after centrifuge, and the fluorescence intensity of 2 × 10^4^ cells was analyzed using the FACSCalibur instrument (BD Biosciences, Franklin Lakes, NJ, USA). The percentage of the internalized receptor was calculated from surface receptor fluorescence values (F) as follows: Receptor internalization rate = (F _Control group_ – F _Drug group_)/(F _Control group_ – F _Negative group_) × 100%.

### Molecular docking

Molecular preparation and docking were performed in Molecular Operating Environment (MOE, Chemical Computing Group Inc., Montreal, Canada) v2018.0101. Crystal structure of human GPR35 protein was predicted based on the AlphaFold Protein Structure Database (https://alphafold.ebi.ac.uk/entry/Q9HC97). Raman diagram was used to evaluate the rationality of protein structures. The conformational rationality ratio of amino acid residues of the predicted protein was 99.2%, indicating that the protein structure was reasonable Fig. S10. The protonation state of the GPR35 protein and the orientation of the hydrogens were optimized by LigX module at the PH of 7 and temperature of 300 K. The AMBER10: EHT force field was used to ensure the protein energy minimization. Using KA as the ligand and the optimized GPR35 predicted protein as the receptor for molecular docking around R151 site under the DOCK module in MOE [20]. The docking workflow followed the “induced fit” protocol, in which the side chains of the receptor pocket were allowed to move according to ligand conformations, with a constraint on their positions. The molecular binding conformations were ranked by London dG scoring, and then a force field refinement was carried out on the top 10 poses followed by a rescoring of GBVI/WSA dG. PyMol software was used for visualization.

### Animal experiments and sample collection

All animal studies and procedures were conducted in accordance with the US National Institutes of Health Guide for the Care and Use of Laboratory animals and approved by the Animal Ethics Committee of Guangdong Medical University (Approval No.: GDY2304013). Male Sprague-Dawley (SD) rats at the age of 6–8 weeks were purchased from Guangdong Province medicine experimental animal center, Permission No.: SCXK (Yue) 2018–0002). All rats were housed in a temperature-controlled environment (room temperature of 23 ± 2 °C; relative humidity of 50 ± 5%) for a week acclimation with a standard rodent diet under 12/12 h dark/light cycle before the experiments started.

The animal experiment procedures are carried out in accordance with Fig. S11. All rats were randomly assigned to each group (*n* = 18). Drug treatment groups were pretreated with inhibitors and KA for 2 days, and then rats were administered with 5% (w/v) dextran sulfate sodium (DSS, MW = 36-50 kDa) (APExBIO, Houston, TX, USA) drinking water for 7 days. Control group rats were received normal drinking water and administered with saline or vehicle. On day 10, drug treatment group and model group rats were given normal drinking water, and the inhibitor administrations were terminated on day 17 and the experiment ended after a week. KA intervention was continued throughout the experiment in KA treatment groups. Body weight (BW) and DAS were recorded daily. The DAS was determined by combining the scores for body weight loss, stool consistency and gross bleeding according to the previous reports [38]. On day 10, 17 and 24 six rats that were randomly selected in each group were sacrificed and colon tissues were collected. A portion of colon tissue was fixed in 10% formalin for histological examination and the rest was stored at −80 °C for subsequent experiment.

The relative (Rel.) BW growth rate and DAS reduction rate were calculated as follows: Rel. BW growth rate = (*W*_End time_ – *W*_Start time_)/(D × *W*_Start time_) × 100%. *W*_Start time_ and *W*_End time_, represent the rat weight at the start and end of each administrative period; D, represents the number of corresponding administrative days. DAS reduction rate = (*S*_Start time_ – *S*_End time_)/D × 100%. *S*_Start time_ and *S*_End time_, represent the DAS at the start and end of each administrative period, the data are counted before the DAS is restored to 0.

### Enzyme-linked immunosorbent assay

About 20 mg of each colon tissue sample was homogenized with precooled saline in a ratio of 1:10 (w/v, mg/μL) using a high-speed tissue homogenizer (Biopre-24 Homogenizer, Shanghai, China). The homogenization conditions were 7 stainless steel beads, vibration speed of 7.0, and 4 cycles. After centrifuge twice at 4 °C, 14,000 rpm, the supernatant was collected for cytokine detection. Rat IL-6, IL-1β, TNF-α levels in colon tissue were measured by enzyme-linked immunosorbent assay (ELISA) (Invitrogen, Carlsbad, CA, USA) according to the manufacturer’s instructions. The relative inflammatory factor reduction was calculated as follows: Rel. inflammatory factor reduction = (*L*_Start time_ – *L*_End time_)/*L*_Start time_ × 100%. *L*_Start time_ and *L*_End time_, represent the relative level of inflammatory factor in the colon at the start and end of each administrative period.

### Intestinal permeability analysis

After treatment, rats were jejunitas for 6 h. Rats were administered by FITC-dextran (MW: 4000, Sigma-Aldrich, St. Louis, MO, USA) with a concentration of 100 mg/mL in water at a dosage of 750 mg/kg through oral gavage. Four hours later, rat blood samples were collected from vena ophthalmica. Quiescence, centrifugation, and the serum were obtained. The fluorescence intensity of serum was detected using a multimode reader (Thermo Fisher Scientific, Waltham, MA, USA) at an excitation of 485 nm and emission wavelength of 535 nm. The concentration of FITC-dextran in the serum was calculated according to a standard curve. The relative FITC-dextran reduction was calculated as follows: Rel. FITC-dextran reduction = (*C*_Start time_ – *C*_End time_)/*C*_Start time_ × 100%. *C*_Start time_ and *C*_End time_, represent the concentration of FITC-dextran in rat serum at the start and end of each administrative period.

### Quantitative real-time polymerase chain reaction (qRT-PCR) analysis

Total RNA in cell samples was extracted by RNAiso Plus (TaKaRa Biotechnology, Dalian, China) according to the manufacturer’s instructions, and their concentration were quantified utilizing NanoDrop 2000 (Thermo Fisher Scientific, Waltham, MA, USA). Total RNA, 1 μg, was reverse transcribed to complementary DNA (cDNA) through PrimeScript^TM^ RT reagent Kit (TaKaRa Biotechnology) following the manufacturer’s instructions. Quantitative real-time PCR was performed using SYBR Green Ι Master (Roche Diagnostics, Basel, Switzerland) on a LightCycler 480 instrument (Roche Diagnostics) according to the manufacturer’s instructions. The gene relative expression was calculated by the 2^–ΔΔCT^ method, and normalized by *β*-actin in each sample. The primers used in qRT-PCR are listed in Table S2.

### Western blotting

The cells and colon tissues were lysed by radioimmunoprecipitation buffer (Sigma-Aldrich) containing 1 mmol/L phenylmethylsulfonyl fluoride (Sigma-Aldrich) on ice and homogenized by a tissue homogenizer (Bioprep-24 homogenizer), respectively. The protein concentrations of lysates were quantified using a BCA protein assay kit (Thermo Fisher Scientific). After denaturalized at 95 °C for 10 min with sample loading buffer (Beyotime Biotechnology, Shanghai, China), the proteins (30−50 mg) were separated using SDS-polyacrylamide gel electrophoresis (Bio-Rad, Hercules, CA, USA) and transferred to polyvinylidene difluoride membranes (Millipore, Bedford, MA, USA). The membranes were blocked with 5% milk for 2 h at room temperature and incubated with primary antibodies (Listed in Table S3) at 4 °C overnight, followed by subjected to horseradish peroxidase (HRP)-conjugated secondary antibody (Proteintech, Shanghai, China) for 2 h at room temperature. The protein bands were visualized utilizing an enhanced chemiluminescence (ECL) system (Millipore) on a chemiluminescent imaging apparatus (Amersham ImageQuant 800, MA, USA). The relative protein expression was analyzed by Image J software (NIH, Bethesda, MD, USA).

### Immunohistochemistry, hematoxylin and eosin (H & E) and Alcian Blue (AB) staining

After fixed in 10% buffered formalin for 3 days, colon tissues were performed immunohistochemistry and H&E staining according to conventional methods [39]. Briefly, colon tissues were embedded in paraffin and then sectioned at a thickness of 4 μm. For H&E staining, the sections were prepared orderly by dewaxing with graded xylene and ethanol, staining with H&E solution, and subsequently dehydrating and mounting with permount. For immunohistochemistry, the sections were subjected to deparaffinization, antigen retrieval and blocking with goat serum, followed by incubating with anti-KLF5 rabbit polyclonal antibodies (Abcam, Cambridge, MA, USA) and HRP-conjugated goat anti-rabbit IgG secondary antibody (Proteintech), successively. After counterstaining with hematoxylin, the sections were mounted with permount. The tissue morphology and protein expression of sections were observed and captured using an inverted microscope (Leica, Shanghai, China).

Colon tissues for AB staining were fixed in Carnoy’s fixative (Servicebio, Wuhan, China) for 3 days, and then manufactured into sections. According to traditional methods [40], the sections were subjected to dewaxing with graded xylene and ethanol, and stained with Alcian blue dye solution A and solution B (Sigma, St. Louis, USA), orderly. Subsequently, the sections were dehydrated and mounted, and their microstructures were captured by an inverted microscope (Leica).

### Transcriptome sequencing (RNA-seq)

Total RNA in cell and tissue samples were extracted by RNAiso Plus (TaKaRa Biotechnology, Dalian, China) according to the manufacturer’s instructions, and the RNA quality was assessed using the Agilent 2100 bioanalyzer (Agilent Technologies Inc., Santa Clara, CA, USA), and the RNA concentration was analyzed using the NanoDrop 2000 (Thermo Scientific, Waltham, MA, USA). Transcriptome library was prepared using the NEBNext Ultra II RNA Library Prep Kit for Illumina (New England Biolabs Inc, Ipswich, MA, USA), and assessed using the Bioanalyzer 2100 and the DNA high-sensitivity chip (Agilent Technologies). RNA-seq and data analysis were performed at Shanghai Personal Biotechnology Co., Ltd (Shanghai, China). Paired-end RNA sequencing libraries were sequenced on an Illumina NovaSeq 6000 platform (Illumina Inc.) with 2 × 150 bp read length following standard protocols provided by the company.

Sequencing data were filtered using Fastp (0.22.0) software, and then mapped to the reference genome (http://asia.ensembl.org/Homo_sapiens/Info/Index) using HISAT2 (v2.1.0). We used HTSeq (v0.9.1) statistics to compare the Read Count values on each gene as the original expression of the gene, and then used FPKM (Fragments Per Kilo bases per Million fragments) to standardize the expression. Difference expression of genes was analyzed by DESeq (v1.38.3) with screened conditions as follows: expression difference multiple |log2FoldChange | > 1, *P* < 0.05. R language Pheatmap (v1.0.12) software package was used to perform bidirectional clustering analysis and principal components analysis (PCA) of all differential genes. Differential genes were mapped to Gene Ontology database and Kyoto Encyclopedia of Genes and Genomes (KEGG) database for annotation and visualization of function and pathway enrichment analysis using Gene Set Enrichment Analysis (GSEA) (v4.1.0) tool, the standard of significant enrichment is *P* < 0.05.

The key materials and resources used in all experiments are listed in Table S3.

### Statistical analysis

GraphPad Prism 6.0 Software (GraphPad Software Inc.) was used for all statistical analyses. All experiments were duplicated at least three times, and the results were presented as mean ± standard deviation (S.D.), unless otherwise specified. Independent unpaired two-tailed Student’s *t* test was performed to evaluate the differences between two groups. Multiple group comparisons were analyzed using one-way analysis of variance (ANOVA) with Bonferroni correction. *P* < 0.05 was considered statistically significant.

## Supplementary information

Supplementary Information

Supplementary appendix 1

Supplementary appendix 2

Supplementary appendix 3

## Data Availability

This study does not involve datasets requiring public deposition. All experimental procedures and results are fully described in the manuscript. The other data that support the findings of this study are available from the corresponding authors on reasonable request.
