# Supplementary material for: Tryptophan metabolic gatekeeping in epithelial repair: GPR35-KLF5 circuitry decodes mucosal damage signals for repair programming
Source: Cell Death Dis. 2026 Jan 9;17(1):25. doi: 10.1038/s41419-025-08237-0 (PMC12789062; doi:10.1038/s41419-025-08237-0)

Supplementary Information

Tryptophan metabolic gatekeeping in epithelial repair: GPR35-KLF5 circuitry decodes mucosal damage signals for repair programming

Biao Xie[3,4#], Meimei Wang[1#], Yaping Xiao[1], Xin Zhang[1], Meng Liu[1], Jie Miao[1], Yunfei Mo[1], Hongxin Liu[1], Jihui Wang[1], Fengguo Xu[2]*, Di Wang[1]*

[1] School of Life and Health Technology, Dongguan University of Technology, Dongguan 523808, P.R. China

[2] Key Laboratory of Drug Quality Control and Pharmacovigilance (Ministry of Education), State Key Laboratory of Natural Medicine, China Pharmaceutical University, Nanjing 210009, P. R. China

[3] Department of Gastroenterology, Guangzhou Eighth People's Hospital, Guangzhou Medical University, Guangzhou 510440, P.R. China

[4] Guangzhou Key Laboratory of Clinical Pathogen Research for Infectious Diseases, Guangzhou Eighth People's Hospital, Guangzhou Medical University, Guangzhou 510440, P.R. China

* To whom correspondence should be addressed at School of Life and Health Technology, Dongguan University of Technology, Dongguan 523808, P.R. China (D. Wang). Key Laboratory of Drug Quality Control and Pharmacovigilance, China Pharmaceutical University, Nanjing 210009, P. R. China (F.G. Xu).

E-mail: diwang_cpu@163.com (D. Wang), fengguoxu@cpu.edu.cn (F.G. Xu).

[#] These authors contributed equally to this work

Supplementary Figures

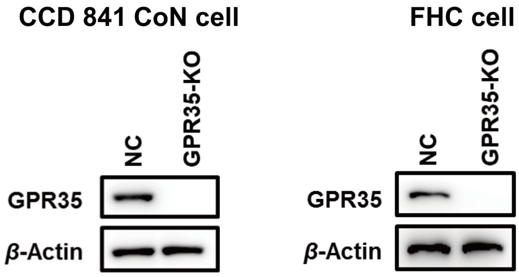

Figure S1. Western blot analysis of the knockout level of GPR35 gene in IECs

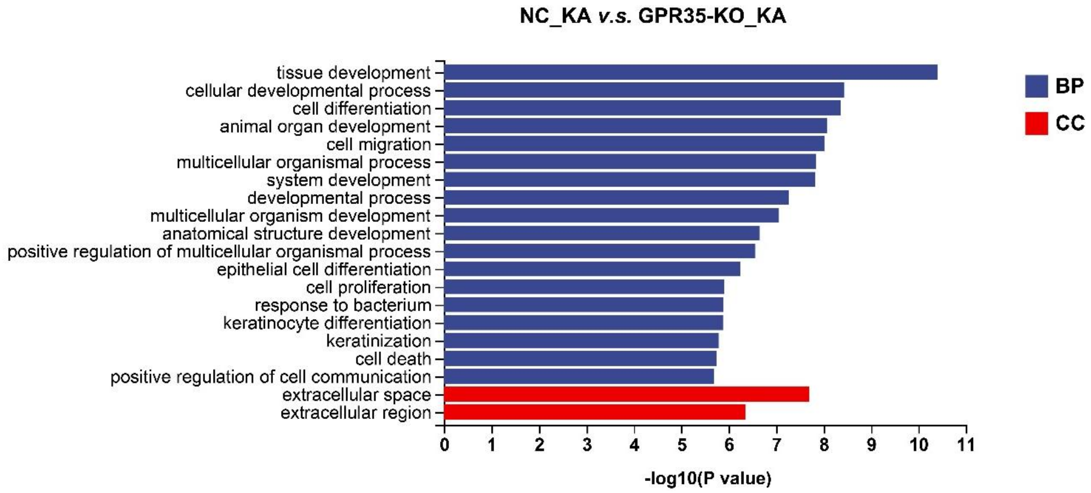

Figure S2. Significant enriched top 20 Gene Ontology (GO) terms based on the differentially expressed genes in normal and GPR35 knockout IECs that were treated with KA. BP, biological process; CC, cellular component.

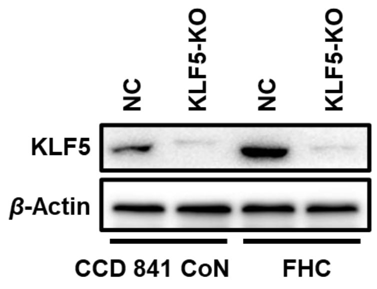

Figure S3. Western blot analysis of the knockout level of KLF5 gene in IECs.

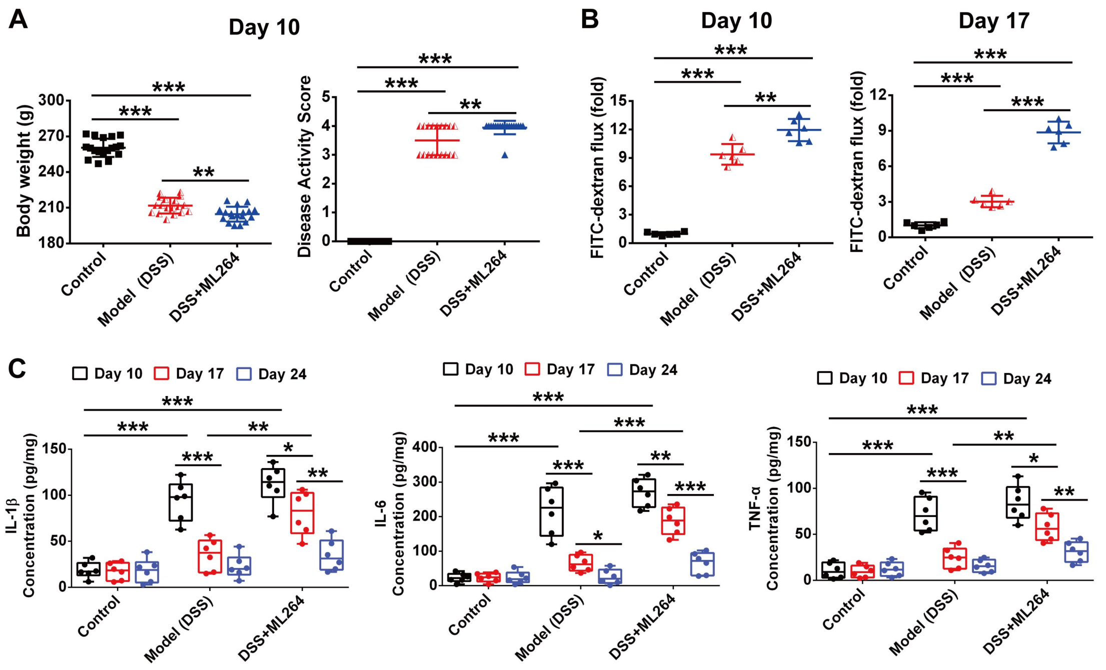

Figure S4. The role of KLF5 in intestinal mucosal damage and repair. (A) Rat body weight and DAS of each group on day 10 (n = 18). (B) Intestinal permeability analysis of each group rat on day 10 and 17 using FITC-dextran method (n = 6). (C) Elisa analysis of the level of inflammatory factor in rat colon (n = 6). Data are presented as means ± SD. Statistical analysis was performed using Student's *t* test. *P < 0.05, **P < 0.01 and ***P < 0.001.

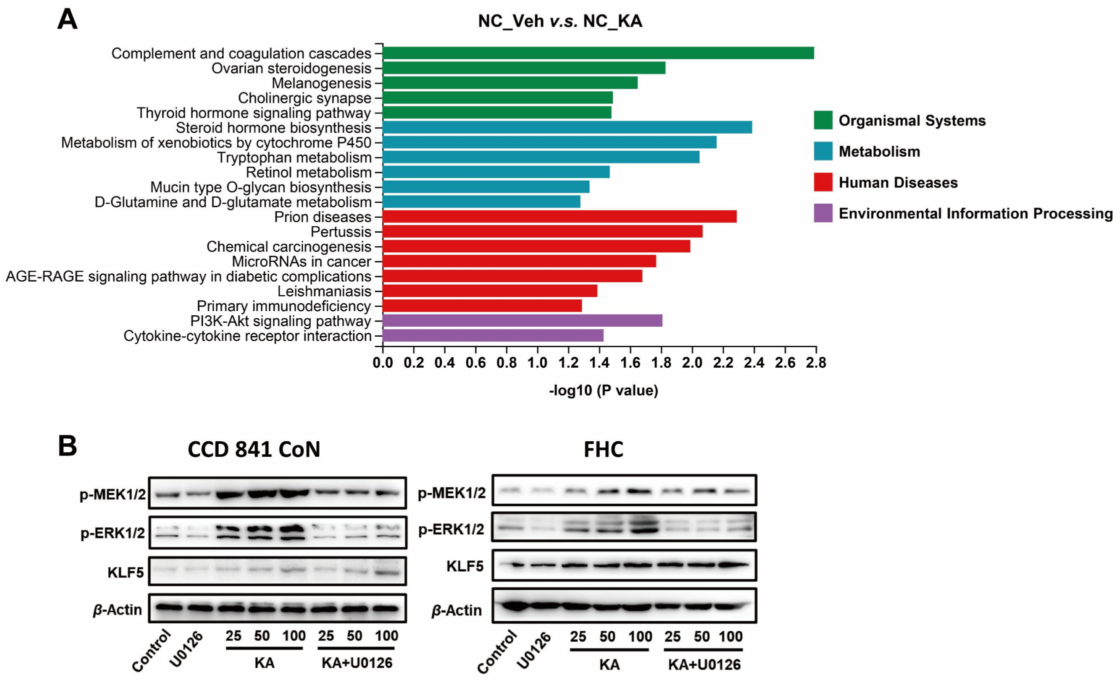

Figure S5. Enrichment and function analysis of pathways. (A) Pathway enrichment analysis of differentially expressed genes between negative control group and KA treatment group based on KEGG database. (B) Effect of specific inhibition of ERK1/2 phosphorylation using U0126 (10 μmol/L) on IECs KLF5 protein expression induced by KA.

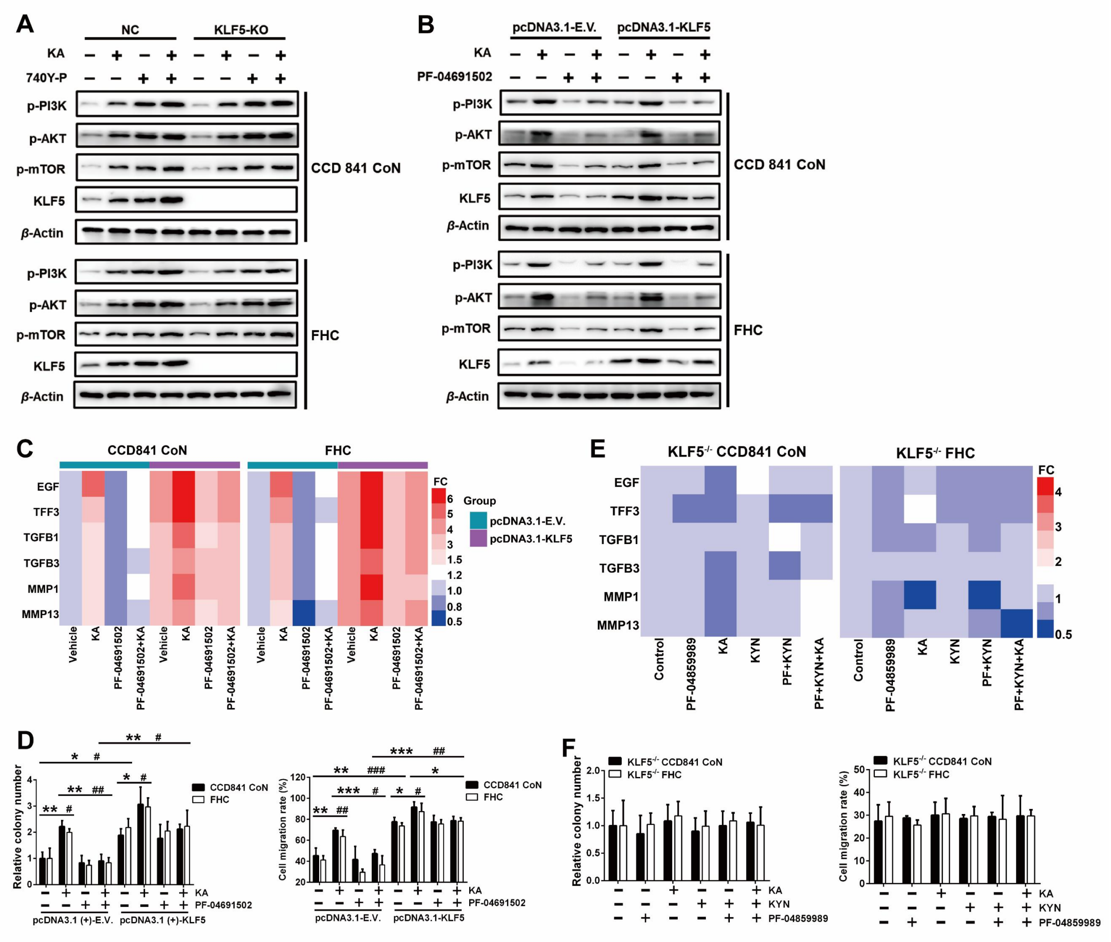

Figure S6. GPR35-mediated KA sensing drives IECs proliferation and migration through KLF5. (A) Western blot analysis of PI3K/AKT pathway activation and KLF5 protein expression in KLF5 normal and deficient IECs that were treated with KA or 740Y-P for 48 h. (B) Western blot analysis of PI3K/AKT pathway activation and KLF5 protein expression in IECs that were transfected with KLF5 overexpression plasmid and following treated with PF-04691502 for 48 h. (C, D) RT-PCR, clone-formation and wound healing analyses of the changes of KLF5 normal and overexpressed IECs proliferation, migration (D) and their related gene expression (C)

respectively after treated with KA or PF-04691502 for 48 h (means ± SD, * and # are represented statistically significance of the data from CCD841 CoN and FHC cells respectively, */#P < 0.05, **/##P < 0.01, ***P < 0.001, Student's t test, n = 3). (E, F) Changes of KLF5 deficient IECs proliferation, migration (F) and their related gene expression (E) after pretreated with (or without) PF-04859989 for 2 h prior to supplemented with KYN and KA treatment for 48 h (means ± SD, Student's t test, n = 3).

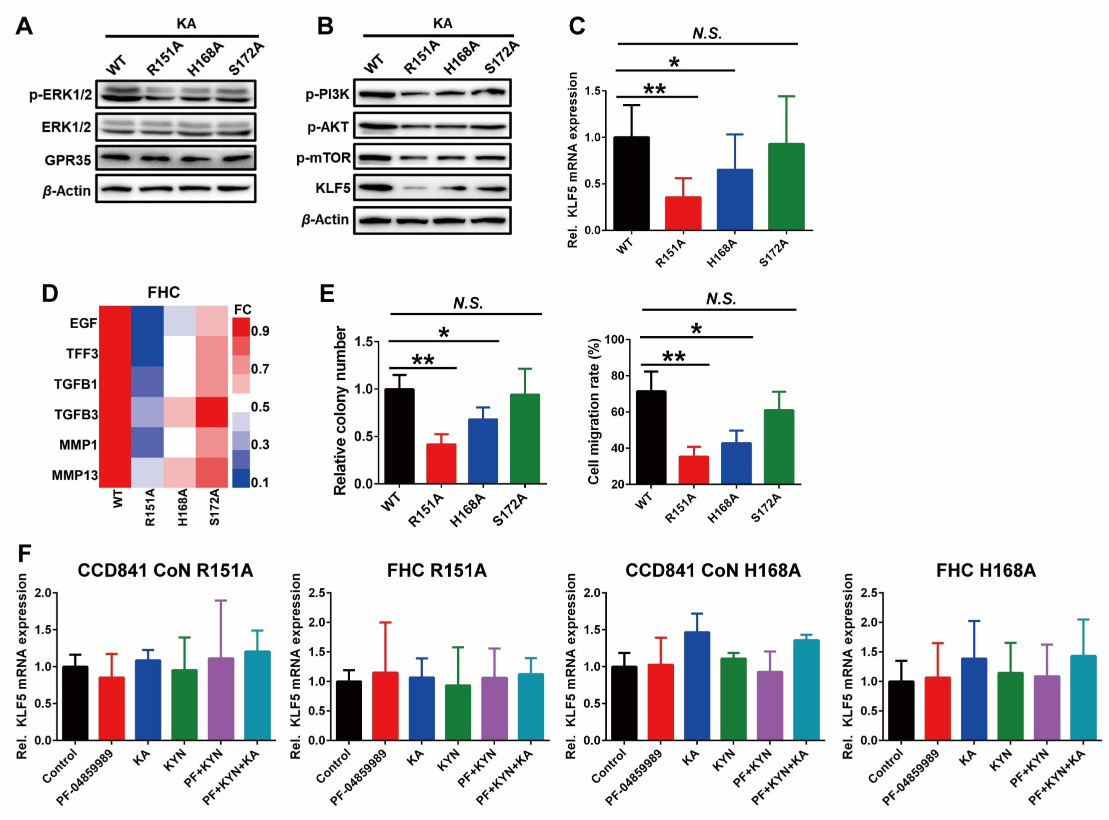

Figure S7. GPR35 mutations influence the signal transduction of Trp metabolic abnormality. (A-C) Western blot and RT-PCR analyses of the effects of KA on ERK1/2 phosphorylation (A), PI3K/AKT pathway activation, KLF5 protein (B) and mRNA (C) expression in GPR35 mutated FHC cells (n = 3-4). (D, E) RT-PCR, clone formation and wound healing analyses of the effects of KA on GPR35 mutated FHC cell proliferation, migration (E) and their related gene expression (D), respectively (n = 3). (F) Change of KLF5 mRNA expression in GPR35 mutated IECs that were pretreated with (or without) PF-04859989 for 2 h prior to supplemented with KYN

and KA treatment for 48 h (n = 3). Data are presented as means ± SD. Statistical analysis was performed using Student's *t* test. *$P < 0.05$, **$P < 0.01$.

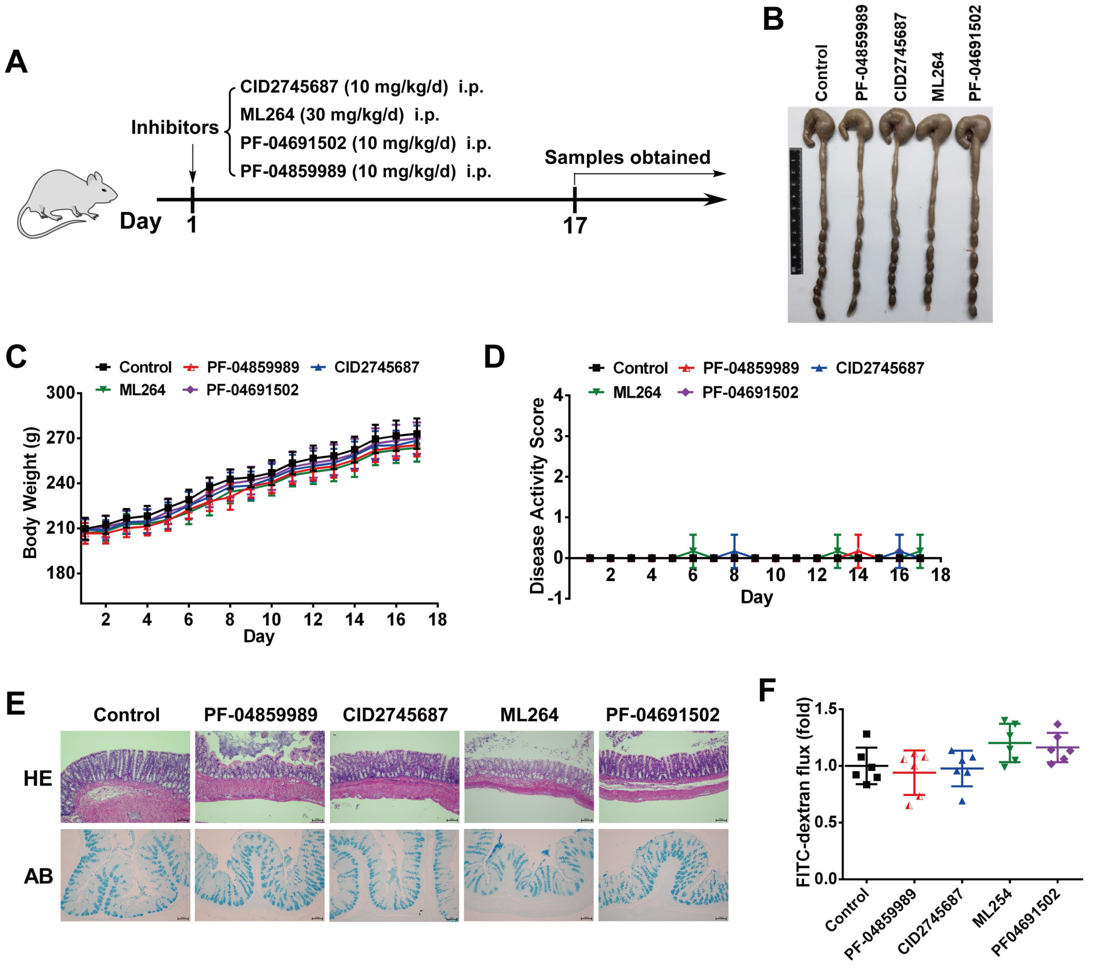

Figure S8. Effects of inhibitors on intestinal homeostasis. (A) Schematic diagram of animal disposition process. (B) Representative images of colon tissues after rat administration. (C, D) The changes of body weight (C) and DAS (D) of each group rats were monitored twice daily. (E) HE and AB staining analyses of rat colon tissues, scale bars = 100 μ m. (F) Intestinal permeability analysis of each group rat after administration using FITC-dextran method. Data are presented as means ± SD. Statistical analysis was performed using Student's t test, n = 6.

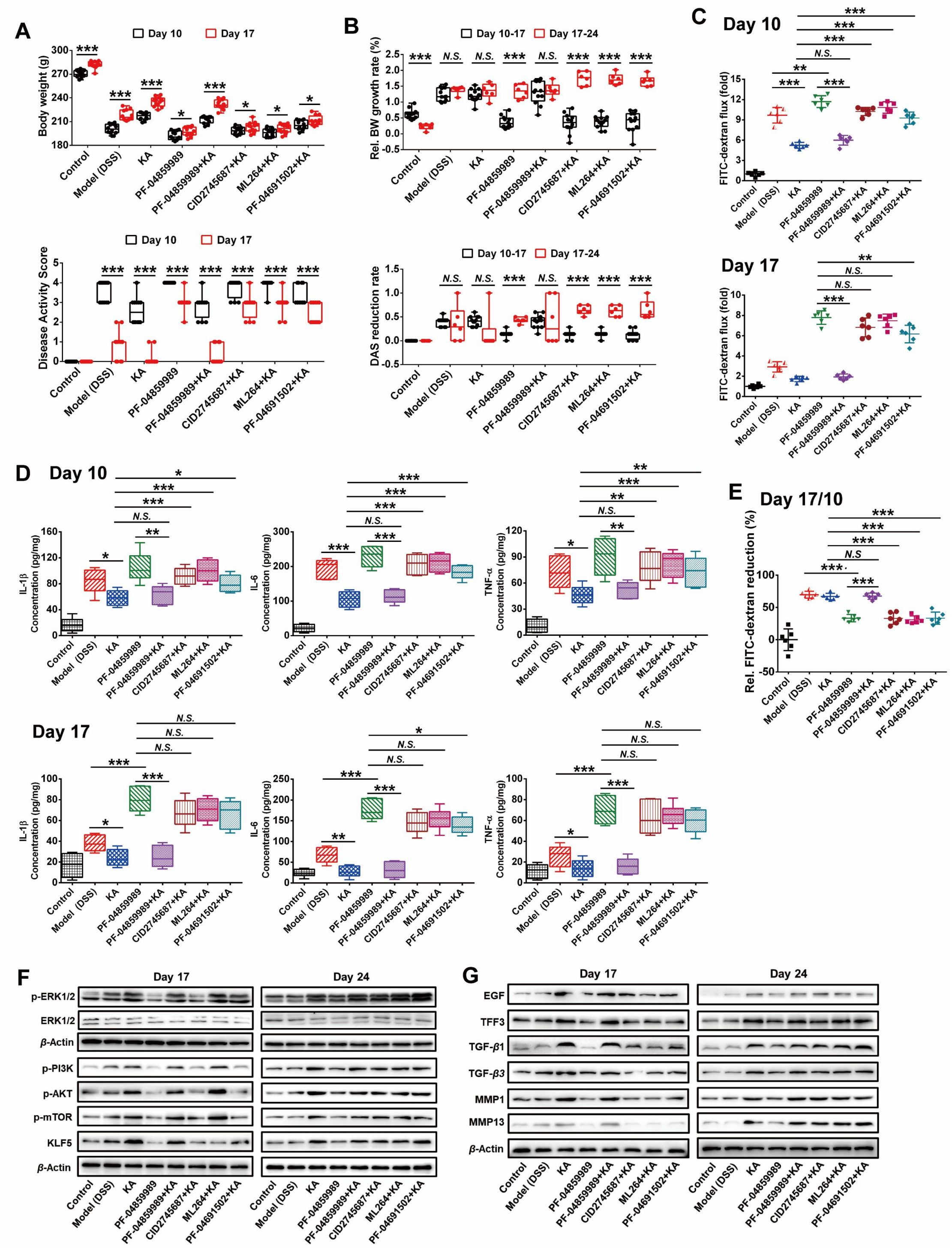

Figure S9. Physiological changes of rats after administration. (A) Rat body weight and DAS of each group on day 10 and 17 (n = 12-18). (B) The relative body weight growth rate and DAS reduction rate during the period of day 10-17 and 17-24 (n = 6-12). (C) Intestinal permeability analysis of each group rat on day 10 and 17 using FITC-dextran method (n = 6). (D) Elisa analysis of the level of inflammatory factor in rat colon (n = 6). (E) The relative variational amplitudes of FITC-dextran level in

serum during the period of day 10-17 was analyzed to reflect the recovery speed of intestinal permeability (n = 6). (F, G) Western blot analysis of ERK1/2 phosphorylation, PI3K/AKT pathway activation, KLF5 and its mediated proliferation and migration related proteins expression in the colon after rat administration on day 17 and 24. Data are presented as means ± SD. Statistical analysis was performed using Student's t test. *$P < 0.05$, **$P < 0.01$ and ***$P < 0.001$.

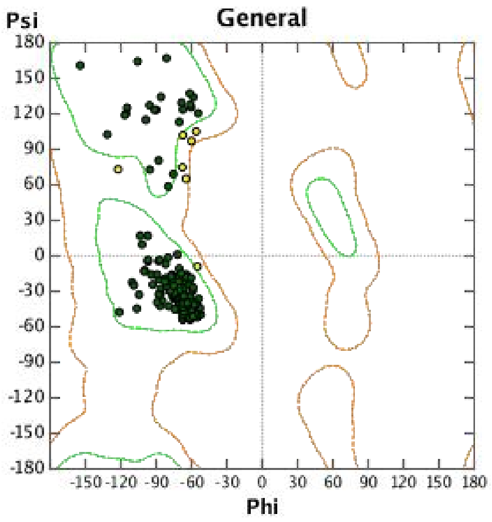

Figure S10. Raman diagram analysis of the rationality of GPR35 protein structure using MOE software.

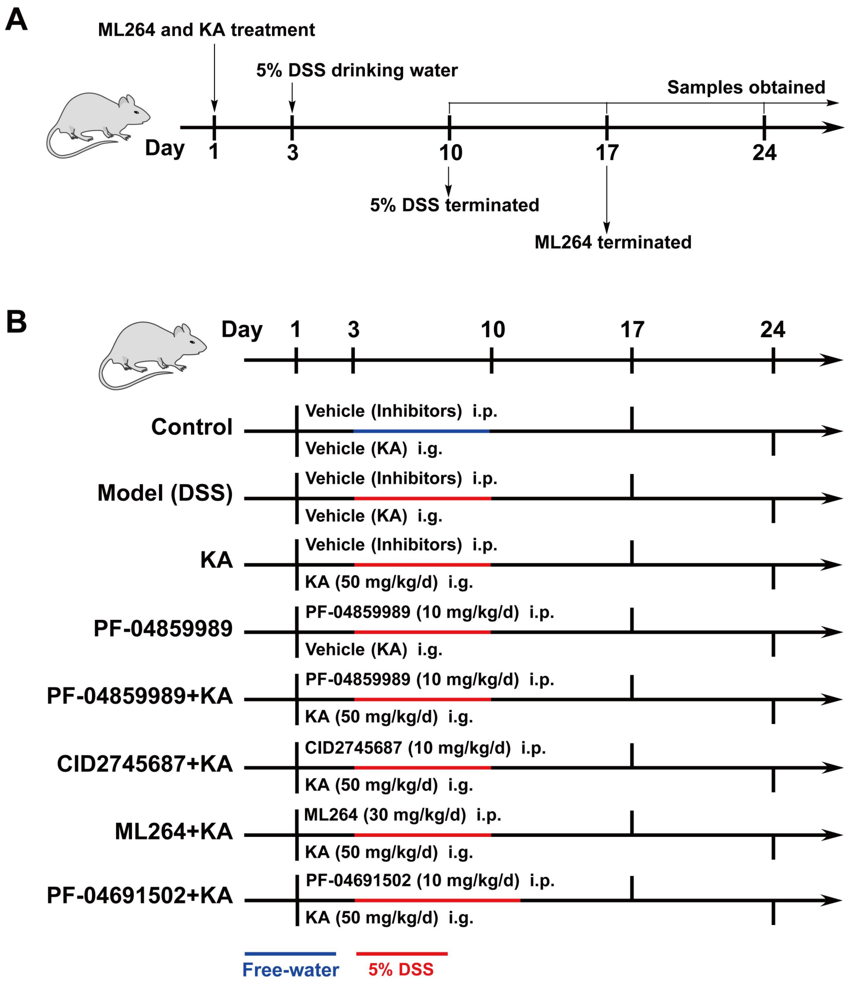

Figure S11. Schematic diagram of animal disposition process. (A) Schematic diagram of administration procedure that research of KLF5 on intestinal mucosal injury and repair *in vivo*. (B) Schematic diagram of KA and inhibitors intervene DSS-induced rat colitis.

Supplementary Tables

Table S1 List of oligonucleotides sequences for lentiviral-mediated knockout.

Oligo	Sequence
LV-EGFP-GPR35-gRNA	5'-gTGCTGCCCCAGGACCATGAAgttttagagctaGAAAtagcaagttaaaataaggc tagtccgttatcaacttgaaaaagtggcaccgagtcggtgcTTTT-3'
LV-EGFP-KLF5-gRNA	5'-gCTCAGCACCCTTGTAGCCATgttttagagctaGAAAtagcaagttaaaataaggct agtccgttatcaacttgaaaaagtggcaccgagtcggtgcTTTT-3'

Table S2 The primer sequences used in qRT-PCR.

Oligonucleotides		Primer sequences	Source
Human KLF5	Forward Primer	5'-TTTGGAGAAACGACGCATCC-3'	Takara
	Reverse Primer	5'-GTGAGTCCTCAGGTGAGCTT-3'	Takara
Human EGF	Forward Primer	5'-CCCTAAGTCGAGACCGGAAGT-3'	Takara
	Reverse Primer	5'-CCATTATCGGGTGAGGAACAA-3'	Takara
Human TFF3	Forward Primer	5'-CAGCTCTGCTGAGGAGTACG-3'	Takara
	Reverse Primer	5'-AGGGATCCTGGAGTCAAAGC-3'	Takara
Human TGFB1	Forward Primer	5'-GGAGAAGAACTGCTGCGTGC-3'	Takara
	Reverse Primer	5'-TCCAGGCTCCAAATGTAGGG-3'	Takara
Human TGFB3	Forward Primer	5'-GAAGAGGGTGGAAGCCATTAGG-3'	Takara
	Reverse Primer	5'-TAGGGCAGACAGCCAGTTCGT-3'	Takara
Human MMP1	Forward Primer	5'-GGACCATGCCATTGAGAAAGC-3'	Takara
	Reverse Primer	5'-TTGTCCCGATGATCTCCCCT-3'	Takara
Human MMP13	Forward Primer	5'-CATCCTCAGCAGGTTGATGC-3'	Takara
	Reverse Primer	5'-GAGGTCATGAGAAGGGTGCT-3'	Takara
Human β-Actin	Forward Primer	5'-CACCCAGCACAATGAAGATCAAGAT-3'	Takara
	Reverse Primer	5'-CCAGTTTTTAAATCCTGAGTCAAGC-3'	Takara

Table S3 The key material and resources used in all experiment.

REAGENT or RESOURCE	SOURCE	IDENTIFIER
Antibodies		
Anti-p-PI3K	Affinity	Cat # AF3242
Anti-ERK1/2	Cell Signaling Technology	Cat # 9102
Anti-p-ERK1/2 (Thr 202/Tyr 204)	Cell Signaling Technology	Cat # 9101
Anti-p-AKT	Cell Signaling Technology	Cat # 9271
Anti-p-mTOR	Cell Signaling Technology	Cat # 2971
Anti-GPR35	Abcam	Cat # ab76217
Anti-KLF5	Abcam	Cat # ab137676
Anti-EGF	Abcam	Cat # ab184265, ab206423

Anti-TFF3	Abcam	Cat # ab108599, ab300427
Anti-FGF7	Abcam	Cat # ab131162
Anti-FGF11	Abcam	Cat # ab89713
Anti-TGF-β1	Abcam	Cat # ab215715
Anti-TGF-β3	Abcam	Cat # ab15537
Anti-MMP1	Proteintech	Cat #10371-2-AP
Anti-MMP7	Proteintech	Cat # 10374-2-AP
Anti-MMP13	Proteintech	Cat # 18165-1-AP
Anti-β-Actin	Proteintech	Cat # 66009-1-lg
CoraLite® Plus 647-conjugated GPR35 Polyclonal antibody	Proteintech	Cat # CL647-55248
HRP-conjugated Affinipure Goat Anti-Mouse IgG(H+L)	Proteintech	Cat # SA00001-1
HRP-conjugated Affinipure Goat Anti-Rabbit IgG(H+L)	Proteintech	Cat # SA00001-2
Virus Strain		
LV-EGFP-GPR35-gRNA lentivirus	Shanghai GenePharma	Report NO.: 21-3742Z, Batch No.: 210331AZ
LV-EGFP-KLF5-gRNA lentivirus	Shanghai GenePharma	Report NO.: 21-3741Z, Batch No.: 210322DZ
LV-EGFP-NC-gRNA lentivirus	Shanghai GenePharma	Report NO.: 21-3744Z, Batch No.: H23YZ
LV-Puro-Cas9	Shanghai GenePharma	Report NO.: 21-3743Z, Batch No.: 210331BZ
LV-Puro-Cas9-NC	Shanghai GenePharma	Report NO.: 21-3819Z, Batch No.: B09EZ
Plasmids		
pcDNA3.1 (+)-GFP-EV	Shanghai GenePharma	OrderID: Y9436 (Attached)
pcDNA3.1 (+)-GFP-KLF5	Shanghai GenePharma	OrderID: Y9436
pcDNA3.1 (+)-GPR35-R151A	Shanghai GenePharma	OrderID: Y11453

pcDNA3.1 (+)-GPR35-H168A	Shanghai GenePharma	OrderID: Y11454
pcDNA3.1 (+)-GPR35-S172A	Shanghai GenePharma	OrderID: Y11455
pcDNA3.1 (+)-GPR35-WT	Shanghai GenePharma	OrderID: Y11456
Chemicals and commercial kits		
L-Kynurenine	Sigma	Cat # K8625
Kynurenic acid	Sigma	Cat # K3375
Puromycin	Sigma	Cat # 540411
Polybrene	Sigma	Cat # H9268
Hydrocortisone	Sigma	Cat #3867
Bovine serum albumin	Sigma	Cat # A1933
FITC-dextran	Sigma	Cat # 46944
PF-04859989	Sigma	Cat # PZ0250
CID2745687	MedChemExpress	Cat # HY-107537
ML264	MedChemExpress	Cat # HY-19994
PF-04691502	MedChemExpress	Cat # HY-15177
Rapamycin	MedChemExpress	Cat # HY-10219
740Y-P	MedChemExpress	Cat # HY-P0175
Cholera toxin	MedChemExpress	Cat # HY-P1446
Crystal violet	MedChemExpress	Cat # HY-B0324A
MK-2206	Selleck	Cat # S1078
MEM medium	GIBCO	Cat # 11095080
DMEM/F12 medium	GIBCO	Cat # 11320033
Fetal bovine serum	GIBCO	Cat # 10100147C
Penicillin-Streptomycin	GIBCO	Cat # 15140-122
Trypsin	GIBCO	Cat # 15050065
NEAA	GIBCO	Cat # 11140050
HEPES	GIBCO	Cat # 15630130
Insulin-Transferrin-Selenium Solution	GIBCO	Cat # 41400045
Lipofectamine 2000	Invitrogen	Cat # 11668019
Dextran sulfate sodium	APExBIO	Cat # B8205

RNAiso Plus	TaKaRa	Cat # 9108
PrimeScript™ RT reagent Kit with gDNA Eraser	TaKaRa	Cat # RR047A
LightCycler® 480 SYBR Green I Master	Roche	Cat # 04 887 352 001
Radioimmunoprecipitation Buffer	Sigma	Cat # R0278
Phenylmethylsulfonyl fluoride	Sigma	Cat # 78830
Hematoxylin	Sigma	Cat # 1.05174
Eosin	Sigma	Cat # 1.17081
Alcian blue staining solution	Sigma	Cat # TMS-010
Loading buffer	Beyotime Biotechnology	Cat # P0015
SDS-PAGE gel preparation kit	Beyotime Biotechnology	Cat # P0012A
ECL Ultra Western HRP Substrate	Millipore	Cat # WBULS0100
PageRuler prestained protein ladder	Thermo Fisher Scientific	Cat # 26617
BCA protein assay kit	Thermo Fisher Scientific	Cat # 23235
Rat IL-6 ELISA Kit	Invitrogen	Cat # BMS625
Rat TNF-α ELISA Kit	Invitrogen	Cat # KRC3011
Rat IL-1β ELISA Kit	Invitrogen	Cat # BMS630
Cell Lines		
CCD841 CoN cell line	ATCC	Cat # CRL-1790
FHC cell line	ATCC	Cat # CRL-1831
Animal		
Sprague-Dawley male rat	Guangdong Province medicine experimental animal center	Cat # 101

Oligonucleotides		
Primers for RT-qPCR: see Tables S2	Takara	N/A
Software and Algorithms		
GraphPad Prism 6.0	Graphpad	https://www.graphpad.com/scientificsoftware/prism/
ImageJ Software	Open source	https://imagej.net/Welcome
HemI. Heatmap Illustrator	Open source	http://hemi.biocuckoo.org/down.php
NCBI Gene Database	Open source	https://www.ncbi.nlm.nih.gov/gene/
Eukaryotic Promoter Database	Open source	https://epd.expasy.org/epd
AlphaFold Protein Structure Database	Open source	https://alphafold.ebi.ac.uk/
Kyoto Encyclopedia of Genes and Genomes	Open source	https://www.kegg.jp/

Other supporting materials for this manuscript include the following:

Data S1. The data of transcription factor binding sites analysis

See Supplementary appendix 1 for details.

Data S2. Clinical data of the ten patient donors

See Supplementary appendix 2 for details.

Data S3. Original data files_original western blots

See Supplementary appendix 3 for details.

Data S4. Representative images of IECs proliferation and migration

The effect of GPR35-mediated KA sensing on IECs proliferation and migration

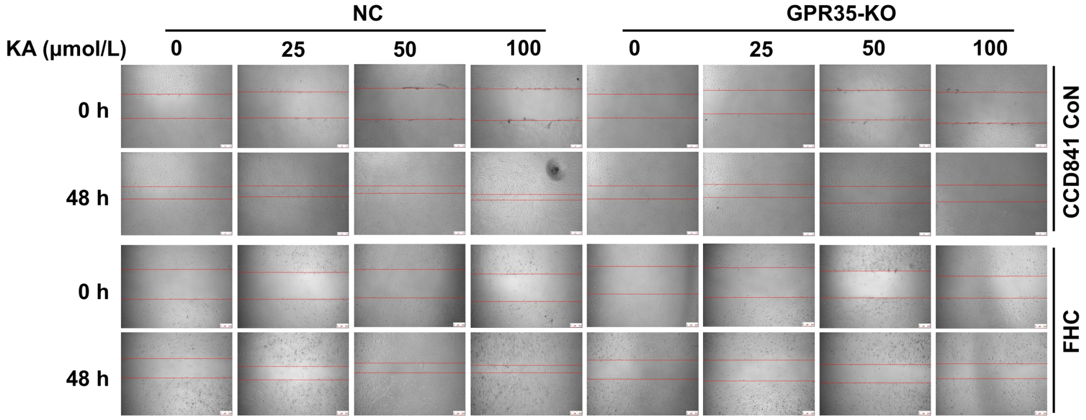

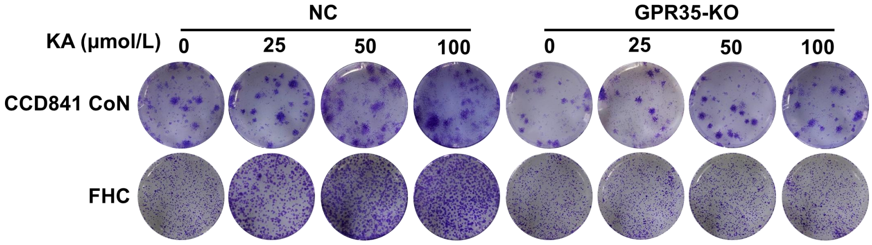

The role of KLF5 played in IECs proliferation and migration driven by KA.

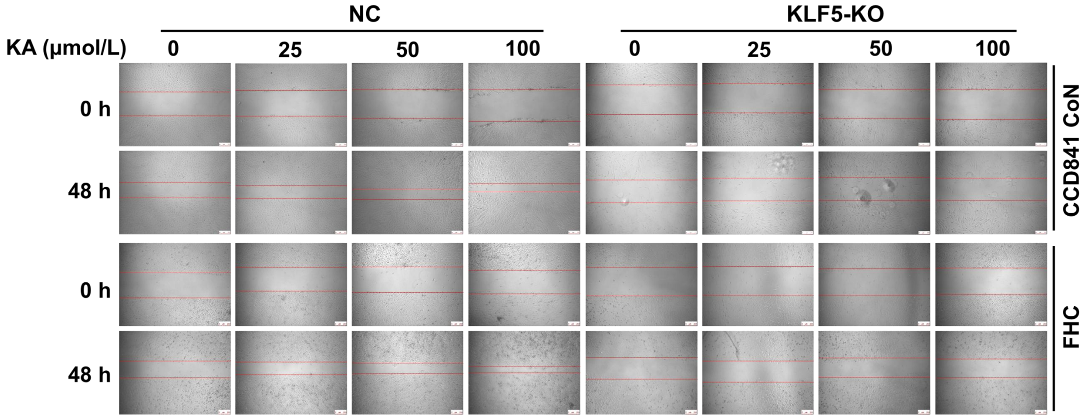

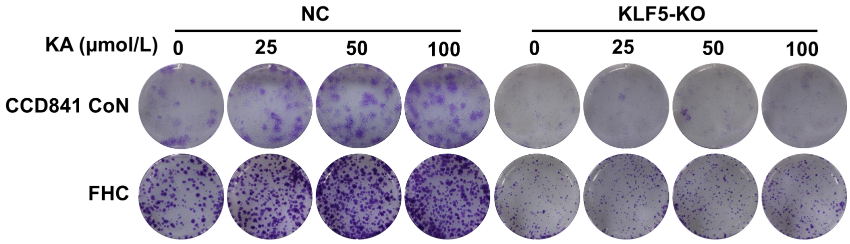

The effect of overexpressing KLF5 in GPR35 deficient IECs on KA-induced proliferation and migration.

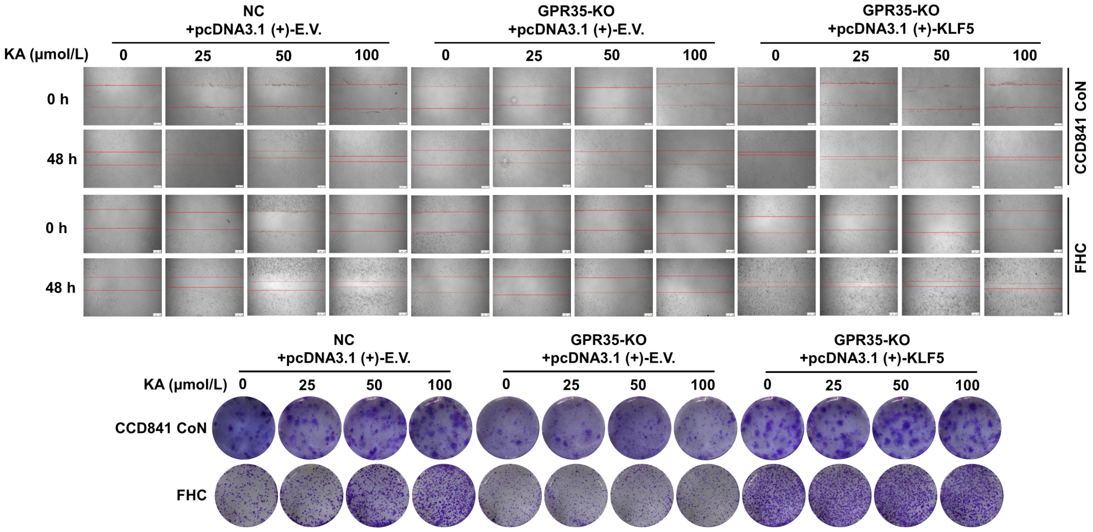

The role of PI3K/AKT pathway played in IECs proliferation and migration driven by KA.

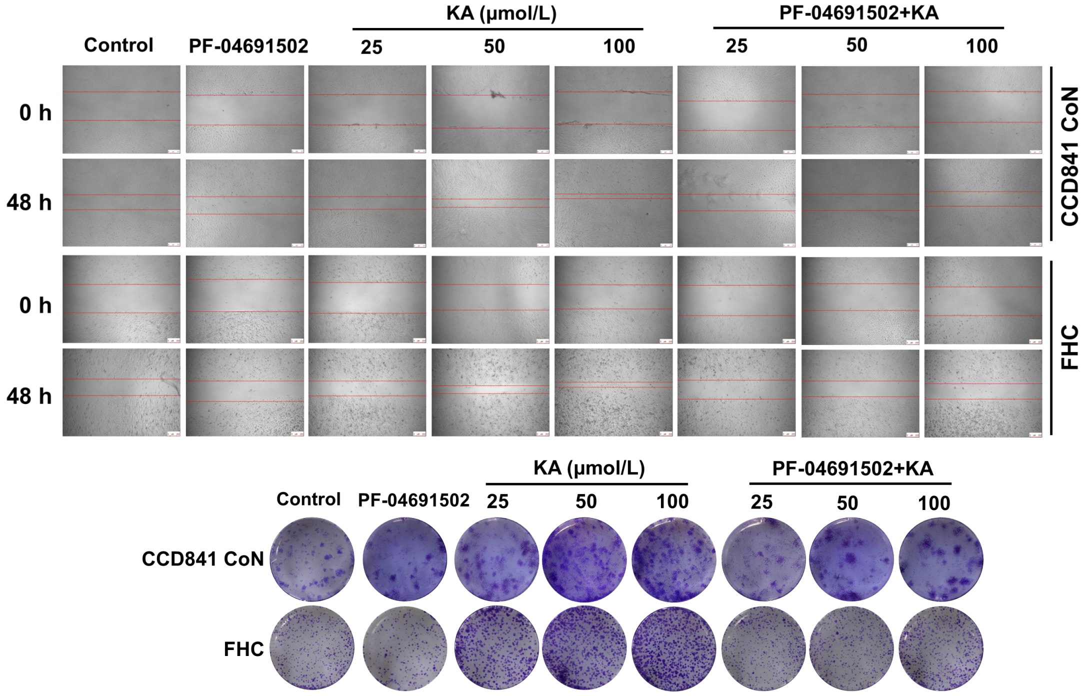

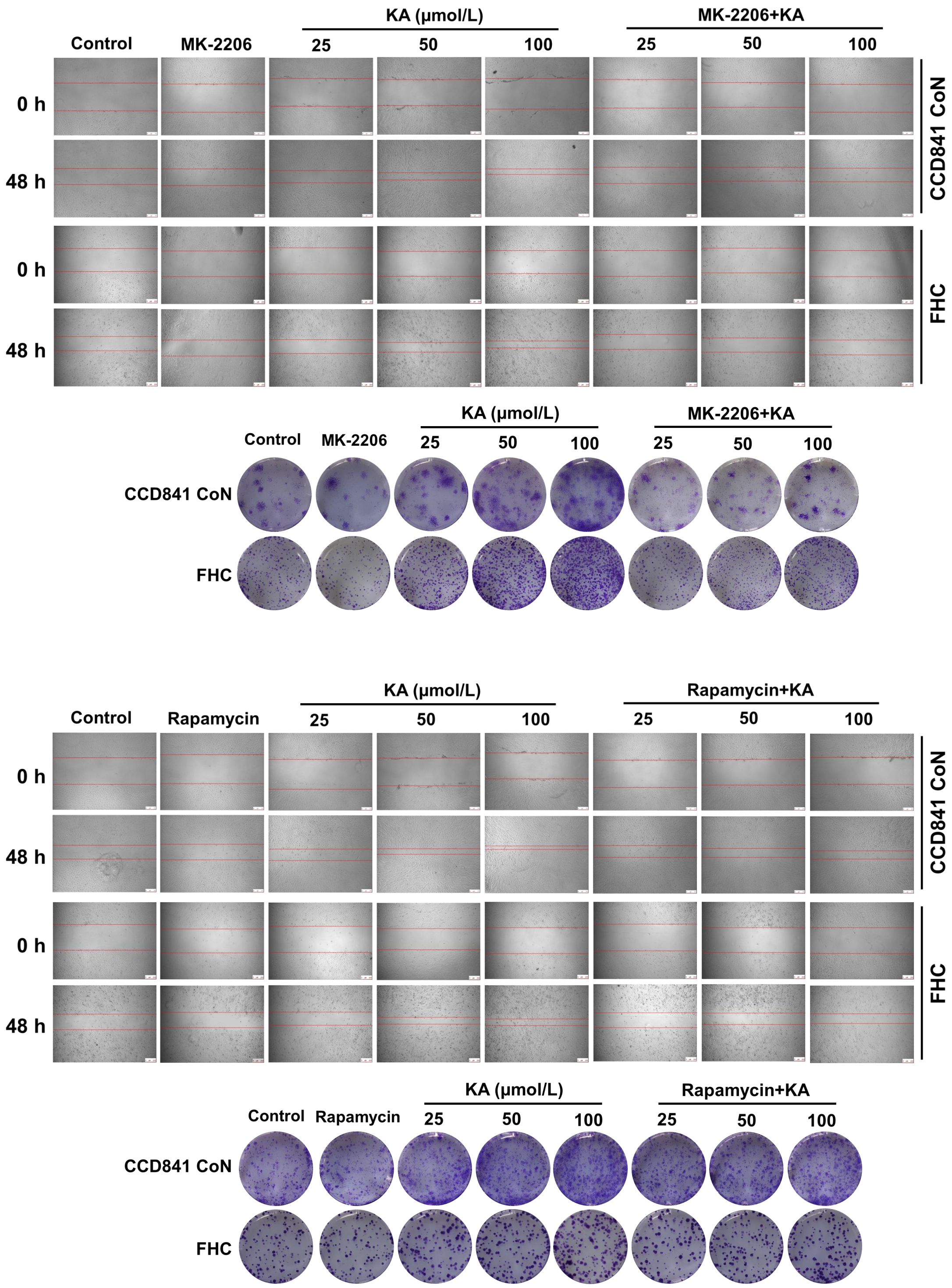
KA (μmol/L)
MK-2206+KA
Control
MK-2206
25
50
100
0 h
48 h
CCD841 CoN
FHC
Rapamycin
Rapamycin+KA

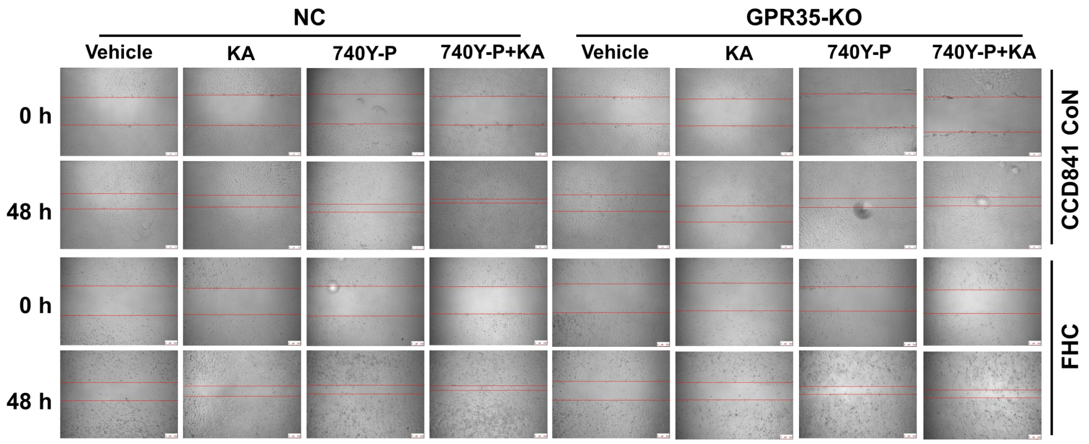
NC
GPR35-KO
Vehicle
KA
740Y-P
740Y-P+KA
Vehicle
KA
740Y-P
740Y-P+KA
0 h
48 h
0 h
48 h
CCD841 CoN
FHC

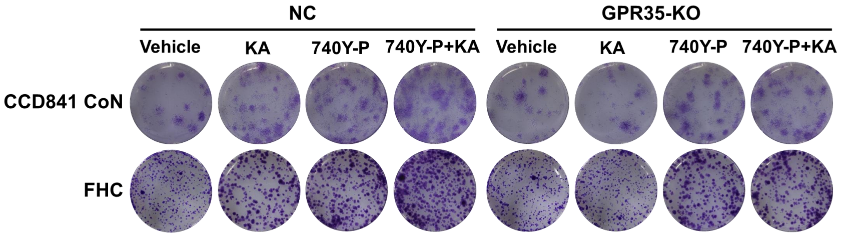
NC
GPR35-KO
Vehicle
KA
740Y-P
740Y-P+KA
Vehicle
KA
740Y-P
740Y-P+KA
CCD841 CoN
FHC

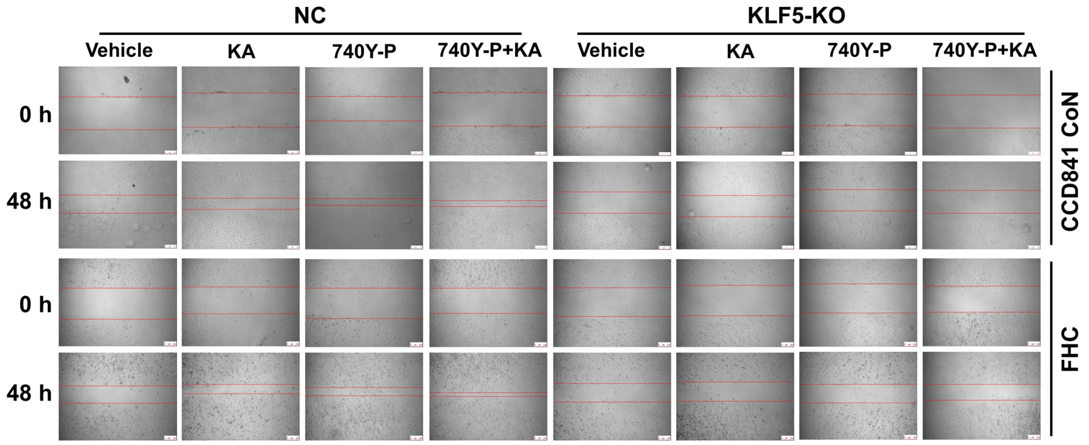
NC
KLF5-KO
Vehicle
KA
740Y-P
740Y-P+KA
Vehicle
KA
740Y-P
740Y-P+KA
0 h
48 h
0 h
48 h
CCD841 CoN
FHC

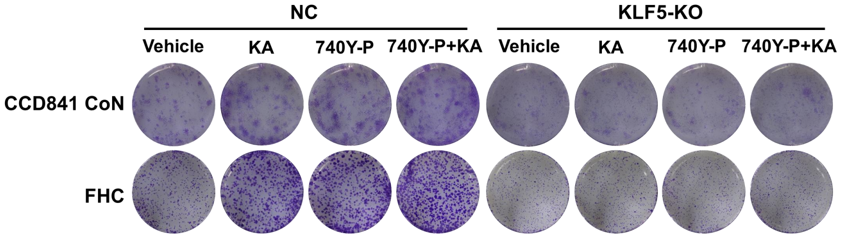
NC
KLF5-KO
Vehicle
KA
740Y-P
740Y-P+KA
Vehicle
KA
740Y-P
740Y-P+KA
CCD841 CoN
FHC

The effect of abnormal Trp-KYN-KA axis metabolism on GPR35 wild type IECs proliferation and migration.

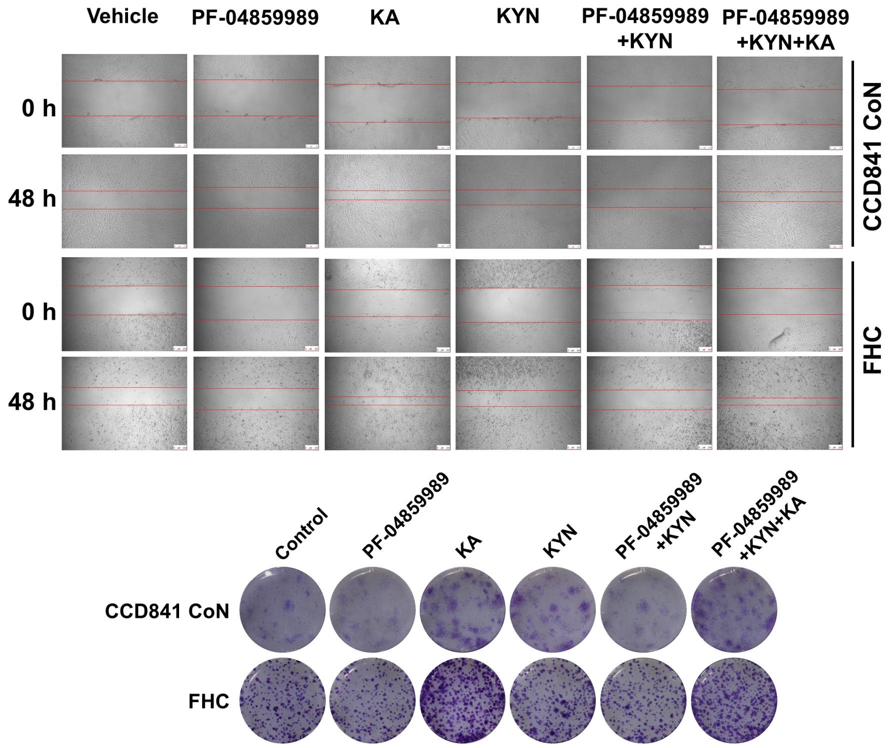

The effect of abnormal Trp-KYN-KA axis metabolism on GPR35 defective IECs proliferation and migration.

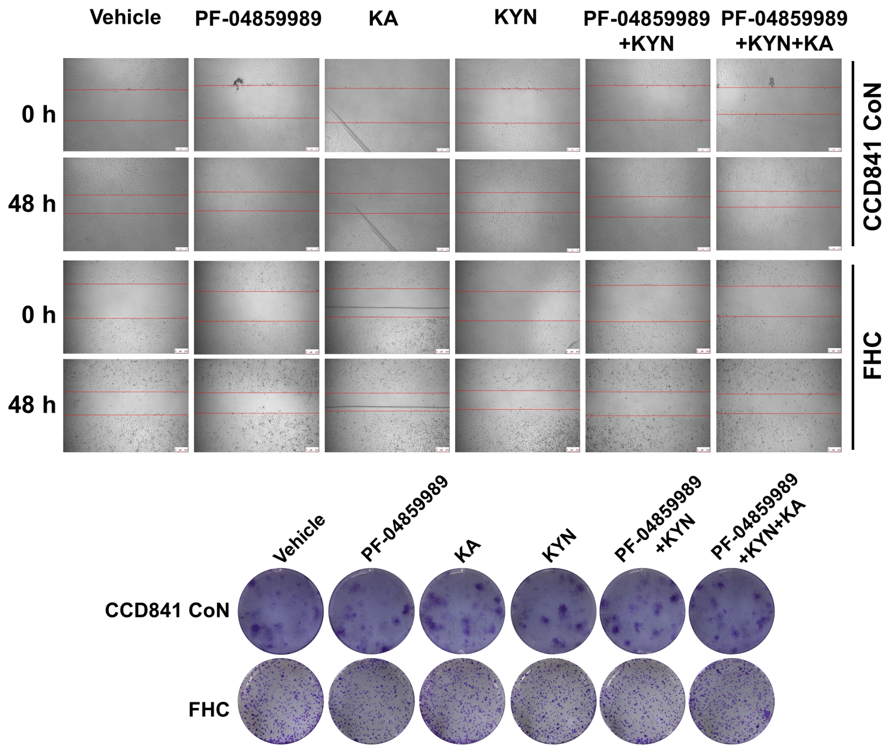

The effect of abnormal Trp-KYN-KA axis metabolism on KLF5 defective IECs proliferation and migration.

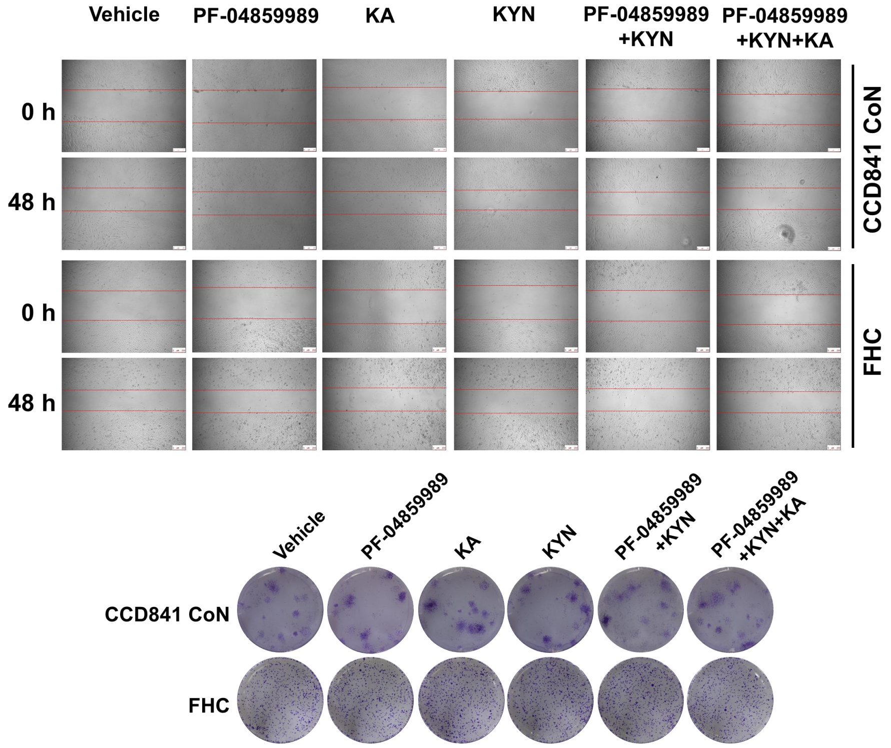

The effect of abnormal Trp-KYN-KA axis metabolism on KLF5 overexpressed IECs proliferation and migration.

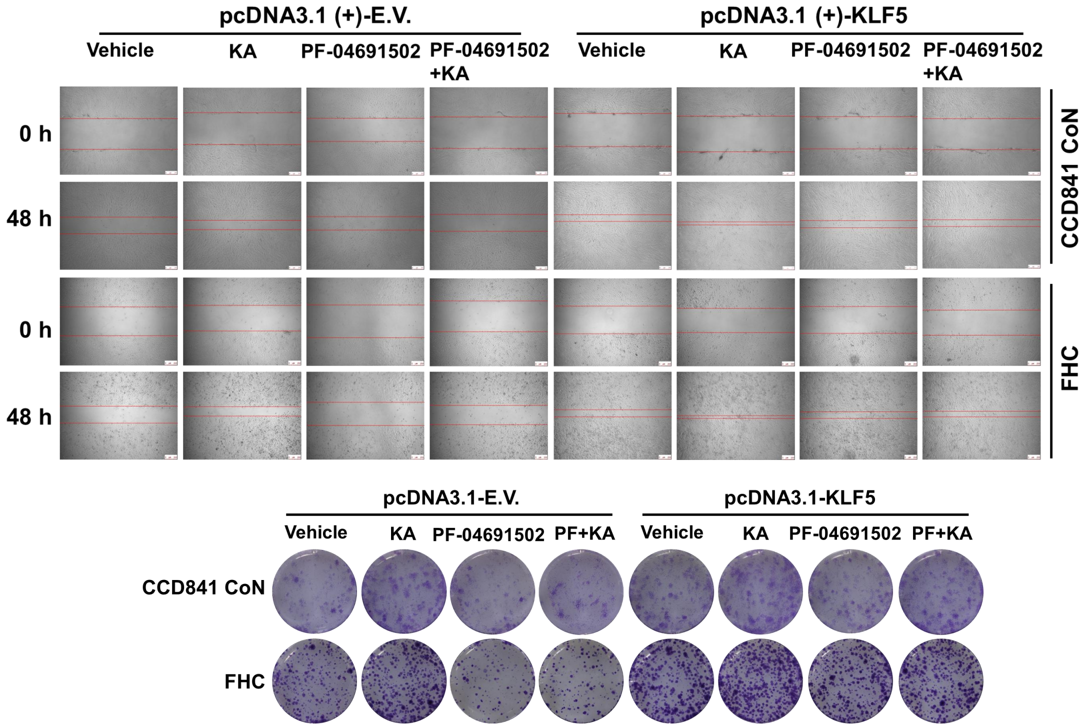

The effect of GPR35 mutation on IECs proliferation and migration

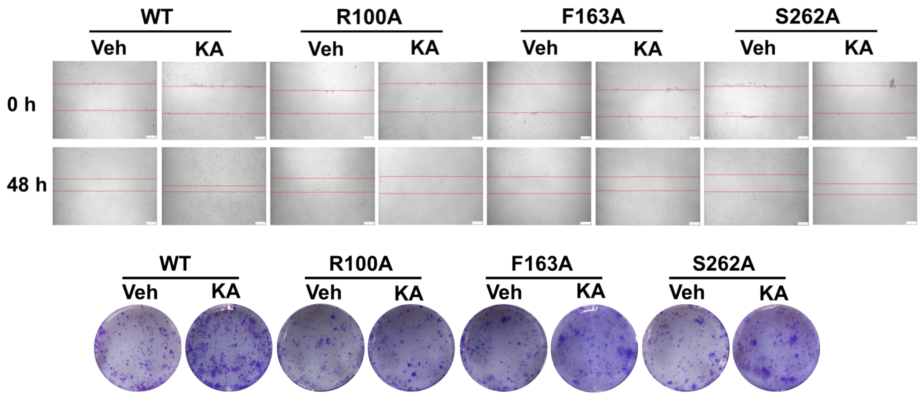

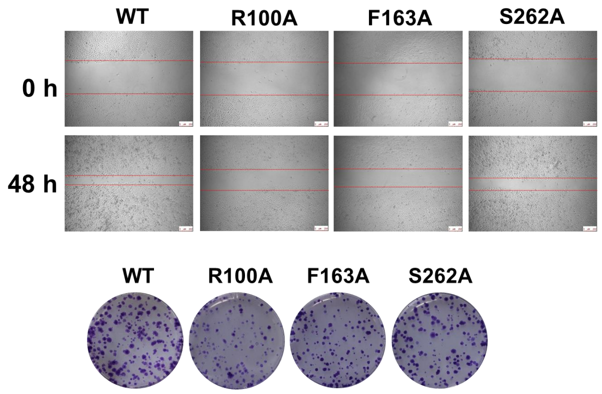

Supplement: Supplementary file 1 — Supplementary Information [file 41419_2025_8237_MOESM1_ESM.pdf]
