# Supplementary material for: Tryptophan metabolic gatekeeping in epithelial repair: GPR35-KLF5 circuitry decodes mucosal damage signals for repair programming
Source: Cell Death Dis. 2026 Jan 9;17(1):25. doi: 10.1038/s41419-025-08237-0 (PMC12789062; doi:10.1038/s41419-025-08237-0)
Supplement: Supplementary file 2 — Supplementary appendix 1 [file 41419_2025_8237_MOESM2_ESM.pdf]

Data S1. The data of transcription factor binding sites analysis

Transcription factors	Genes					
	EGF	TGFB1	TGFB3	TFF3	MMP1	MMP13
ALX3	0	0	0	0	0	2
ARNT::HIF1A	0	4	4	2	0	0
ASCL1	4	14	15	4	11	0
ATF4	2	0	0	2	3	1
ATF7	0	0	0	0	0	0
Ahr::Arnt	0	2	3	1	0	0
Alx1	1	0	0	0	0	2
Alx4	0	0	0	0	1	2
Ar	2	1	2	0	3	2
Arid3a	4	0	1	2	0	1
Arid3b	1	0	0	1	0	0
Arid5a	3	0	1	1	1	2
Arnt	0	3	1	2	0	0
Arntl	3	3	1	3	1	1
Arx	1	0	0	1	0	0
Ascl2	0	11	9	3	6	1
Atf1	1	1	0	0	0	1
Atf3	1	1	1	0	3	2
Atoh1	1	2	0	3	0	2
BACH2	2	4	0	7	2	3
BARHL2	2	0	1	1	0	3
BARX1	1	0	0	0	0	2
BATF3	1	1	0	1	0	2
BATF::JUN	2	1	0	5	7	2
BCL6B	2	2	1	2	2	4
BHLHE22	0	0	0	3	0	0
BHLHE23	2	0	0	3	0	1
BHLHE40	2	5	0	4	2	1
BHLHE41	2	4	0	6	4	1
BSX	1	0	0	0	0	2
Bach1::Mafk	4	2	6	3	2	1
Barhl1	1	0	1	1	0	1
Bcl6	5	1	5	4	2	4
Bhlha15	0	8	3	2	0	4
CDX1	2	0	0	1	2	2
CDX2	2	0	3	4	2	3
CEBPA	2	0	2	1	2	1
CEBPB	0	0	2	2	1	0
CEBPD	0	0	1	2	1	1
CEBPE	0	0	2	2	1	0
CEBPG	0	0	1	1	0	0
CENPB	0	4	0	1	0	1
CLOCK	1	3	5	6	0	0
CREB1	0	2	2	2	0	4
CREB3	1	2	1	1	0	1
CREB3L1	0	3	0	1	1	3
CTCF	2	12	6	4	5	2
CTCFL	4	30	12	7	8	4

CUX1	4	0	0	4	0	0
CUX2	4	0	0	4	0	0
Creb3l2	1	6	5	2	0	2
Creb5	0	0	0	0	0	0
Crem	1	1	0	0	0	1
Crx	3	1	0	0	3	2
DBP	0	0	0	0	0	0
DLX6	1	0	0	1	0	2
DMRT3	1	0	0	0	1	5
DUX4	2	0	1	1	3	5
DUXA	0	0	0	2	3	1
Ddit3::Cebpa	1	2	0	3	2	4
Dlx1	2	0	0	1	0	2
Dlx2	1	0	0	1	0	2
Dlx3	1	0	0	1	0	2
Dlx4	1	0	0	1	0	2
Dmbx1	1	2	1	1	2	4
Dux	1	0	1	1	4	0
E2F1	2	4	0	1	0	0
E2F2	0	1	0	0	0	2
E2F3	3	0	0	0	0	1
E2F4	1	11	2	1	0	0
E2F6	2	9	17	4	7	6
E2F7	2	7	2	5	2	2
E2F8	3	3	2	3	1	3
EBF1	1	12	5	3	3	5
EGR1	4	6	7	4	2	2
EGR2	1	9	7	1	1	0
EGR3	1	15	9	4	6	0
EGR4	1	12	9	5	3	1
EHF	1	2	3	3	2	2
ELF1	3	2	3	3	1	2
ELF3	4	1	2	0	1	2
ELF4	2	2	2	3	0	2
ELF5	4	2	4	5	1	4
ELK1	1	2	2	3	0	1
ELK3	1	2	3	3	0	1
ELK4	4	2	6	6	2	2
EMX1	0	0	0	0	1	2
EMX2	0	0	0	0	0	2
EN1	0	0	0	0	0	2
EN2	0	0	1	1	1	2
EOMES	1	0	2	4	2	1
ERF	2	2	1	4	2	0
ERG	3	2	3	4	1	0
ESR1	2	4	3	5	6	2
ESR2	0	5	3	3	7	2
ESRRB	1	1	3	0	5	5
ESX1	1	0	0	1	0	2
ETS1	3	3	4	3	1	1
ETV1	0	2	2	3	1	2

ETV2	5	3	5	6	1	2
ETV3	1	4	2	4	0	1
ETV4	3	2	2	4	1	2
ETV5	1	2	2	3	1	0
ETV6	2	2	6	3	2	5
EVX1	0	0	0	0	4	2
EVX2	0	0	0	0	4	2
EWSR1-FLI1	7	10	16	3	1	6
Esrra	1	1	3	0	3	2
Esrrg	1	2	2	0	2	3
FEV	3	2	2	3	1	1
FIGLA	1	15	8	5	4	0
FLI1	2	2	3	4	1	1
FOS	1	1	0	3	5	2
FOS::JUN	2	2	0	5	3	4
FOS::JUNB	2	2	0	5	6	4
FOS::JUND	1	1	0	3	3	2
FOS::JUN_var.2	0	0	0	2	0	0
FOSB::JUN	0	0	0	1	0	2
FOSB::JUNB	1	1	0	3	4	2
FOSB::JUNB_var.2	0	0	0	2	3	1
FOSL1	1	3	1	4	4	2
FOSL1::JUN	1	1	0	3	4	2
FOSL1::JUNB	2	2	0	4	4	2
FOSL1::JUND	1	1	0	3	4	2
FOSL1::JUND_var.2	1	0	0	0	2	3
FOSL1::JUN_var.2	0	0	0	2	2	2
FOSL2	2	2	0	5	5	2
FOSL2::JUN	1	1	0	3	3	2
FOSL2::JUNB	1	0	0	3	4	2
FOSL2::JUNB_var.2	0	0	0	0	0	0
FOSL2::JUND	1	1	0	3	3	2
FOSL2::JUND_var.2	0	0	0	1	0	1
FOSL2::JUN_var.2	0	0	0	1	2	1
FOXA1	6	1	3	3	4	4
FOXB1	2	1	3	1	5	3
FOXC1	4	1	4	9	3	6
FOXC2	4	1	5	5	2	6
FOXD1	0	1	4	1	2	6
FOXD2	4	2	2	1	1	6
FOXF2	3	1	4	1	1	8
FOXG1	1	1	3	0	2	6
FOXH1	3	1	0	0	0	4
FOXI1	2	1	3	1	2	6
FOXK1	1	2	5	3	3	5
FOXK2	0	0	4	1	3	5
FOXL1	5	2	2	1	1	6
FOXO3	1	2	4	2	2	4
FOXO4	3	2	2	1	1	5
FOXO6	3	1	2	1	2	5
FOXP1	3	3	4	1	1	6

FOXP2	3	0	5	7	2	6
FOXP3	5	1	2	1	2	6
Foxa2	6	1	4	3	5	5
Foxd3	4	0	3	4	2	3
Foxj2	4	1	1	5	2	6
Foxj3	2	0	3	2	2	4
Foxo1	2	3	1	6	4	5
Foxq1	4	1	2	2	2	6
GATA1::TAL1	3	1	2	1	3	1
GATA2	6	1	1	0	1	1
GATA3	4	1	0	0	2	1
GATA5	4	1	0	0	2	1
GATA6	5	1	3	2	0	1
GBX1	1	0	0	1	0	2
GBX2	1	0	0	1	0	2
GCM1	2	6	2	0	3	1
GCM2	2	9	2	2	1	0
GLI2	1	10	0	4	0	2
GLIS1	1	12	3	1	0	1
GLIS2	2	18	6	6	2	1
GLIS3	2	16	4	7	6	1
GMEB2	2	0	1	0	0	0
GRHL1	0	0	2	0	2	1
GRHL2	2	0	3	0	3	2
GSC2	0	1	0	0	3	3
GSC	0	1	0	0	3	4
GSX1	0	0	0	1	0	2
GSX2	0	0	0	1	1	1
Gabpa	4	3	6	4	1	4
Gata1	4	1	0	0	1	0
Gata4	8	2	6	2	5	2
Gfi1	4	0	3	3	2	0
Gfi1b	5	0	3	4	5	4
Gmeb1	3	0	1	0	0	0
HES5	2	5	5	5	0	0
HES7	0	7	1	1	0	1
HESX1	1	0	0	1	0	2
HEY1	0	9	1	1	1	1
HEY2	0	4	4	3	3	0
HIC2	3	11	4	1	3	3
HIF1A	2	2	1	1	0	1
HINFP	0	18	6	3	2	1
HLF	0	0	0	3	0	0
HLTF	1	0	2	0	0	1
HMBOX1	1	0	0	2	1	2
HNF1A	4	0	0	3	2	1
HNF1B	4	0	0	2	2	1
HNF4G	4	2	6	1	5	2
HOXA10	1	0	1	2	1	3
HOXA13	4	0	0	3	1	1
HOXA2	0	0	0	0	2	2

HOXA5	2	0	0	1	1	4
HOXB13	3	0	1	3	1	3
HOXB2	0	0	0	0	1	2
HOXB3	0	0	0	0	1	1
HOXC10	2	0	0	1	1	2
HOXC11	1	0	0	1	1	1
HOXC12	1	0	0	1	1	2
HOXC13	0	0	1	4	2	1
HOXD11	1	0	0	2	2	2
HOXD12	1	0	0	0	1	2
HOXD13	1	0	0	3	1	2
HSF1	5	0	1	1	0	4
HSF2	7	0	1	0	1	3
HSF4	4	0	1	1	0	2
Hand1::Tcf3	3	4	3	6	5	3
Hes1	0	5	2	4	0	0
Hes2	2	3	3	3	1	0
Hic1	1	6	6	2	1	4
Hmx1	0	0	0	1	2	0
Hmx2	1	1	0	5	1	0
Hmx3	1	0	0	5	1	0
Hnf4a	2	3	4	4	3	3
Hoxa11	2	0	0	2	1	3
Hoxa9	3	1	0	2	2	3
Hoxb5	0	0	0	1	0	0
Hoxc9	2	0	0	2	2	2
Hoxd3	0	0	0	1	0	1
Hoxd8	2	0	0	2	0	3
Hoxd9	2	0	2	2	2	1
ID4	2	14	8	3	6	2
INSM1	1	13	5	7	5	5
IRF1	9	1	6	7	1	7
IRF2	4	1	3	2	0	4
IRF3	2	3	5	4	1	3
IRF4	1	1	2	4	0	2
IRF5	0	0	1	3	0	2
IRF7	2	0	6	4	1	4
IRF8	4	2	2	1	1	3
IRF9	3	0	3	5	0	1
ISL2	0	1	0	1	1	3
ISX	1	0	0	1	0	2
Id2	2	5	0	0	1	0
JDP2	1	1	0	3	3	2
JDP2_var.2	0	0	0	1	0	1
JUN	1	1	1	1	3	1
JUN::JUNB	1	2	0	2	2	2
JUN::JUNB_var.2	0	0	0	2	0	1
JUNB	2	4	0	5	6	2
JUNB_var.2	0	0	0	0	0	0
JUND	1	3	0	4	3	2
JUND_var.2	1	1	0	1	3	3

JUN_var.2	2	1	0	3	6	3
KLF13	4	2	5	0	2	0
KLF14	1	11	7	5	2	1
KLF16	2	21	8	5	4	2
KLF4	2	7	4	4	5	4
KLF5	5	19	11	8	5	4
KLF9	2	12	6	4	4	3
Klf12	0	9	6	3	1	2
Klf1	3	13	8	7	5	3
LBX1	1	0	0	1	0	2
LBX2	1	0	0	1	0	2
LEF1	2	0	4	1	3	0
LHX2	0	0	0	0	0	2
LHX6	0	0	0	0	2	1
LHX9	1	0	1	1	0	2
LIN54	5	0	0	4	3	6
LMX1A	0	0	0	0	0	2
LMX1B	0	0	0	0	0	2
Lhx3	1	0	1	0	1	0
Lhx4	0	0	0	0	1	2
Lhx8	0	0	0	0	2	2
MAF::NFE2	4	0	0	3	2	3
MAFF	2	0	1	2	1	4
MAFG	0	0	1	1	1	3
MAFG::NFE2L1	2	0	1	1	2	3
MAFK	1	0	2	3	1	4
MAX	1	5	3	6	0	0
MAX::MYC	0	2	2	7	1	0
MEF2A	3	0	1	1	2	2
MEF2B	4	0	0	0	3	1
MEF2C	3	0	3	1	2	2
MEF2D	2	0	3	1	1	2
MEIS1	2	4	0	4	1	1
MEIS2	3	4	0	5	1	2
MEIS3	2	2	1	6	2	1
MEOX1	0	0	0	0	1	1
MEOX2	0	0	0	0	1	2
MGA	1	3	2	3	4	1
MITF	2	2	0	12	4	2
MIXL1	1	0	0	1	0	2
MLX	1	3	0	3	2	2
MLXIPL	1	6	1	4	5	1
MNT	1	6	2	6	1	0
MNX1	1	0	0	1	0	1
MSC	1	5	5	4	2	0
MSX1	1	0	0	1	0	2
MSX2	1	0	0	1	0	2
MTF1	0	3	2	2	2	0
MXI1	2	9	5	7	3	0
MYB	0	2	1	0	0	2
MYBL1	1	0	0	0	2	0

MYBL2	1	0	0	1	0	0
MYC	2	9	5	6	3	0
MYCN	2	18	6	8	0	0
MYF6	2	7	4	1	2	2
MZF1	0	10	6	3	3	1
MZF1_var.2	1	5	3	3	2	1
Mafb	2	1	0	1	0	6
Mecom	4	1	3	3	2	1
Mlxip	2	5	3	3	2	0
Msx3	1	0	0	1	0	2
Myod1	4	15	8	5	6	3
Myog	2	11	8	3	8	4
NEUROD1	2	15	7	3	6	3
NEUROD2	1	1	1	6	0	1
NEUROG2	0	2	0	7	1	2
NFAT5	10	1	4	1	0	4
NFATC1	8	0	3	2	1	6
NFATC2	8	0	4	1	2	4
NFATC3	8	0	3	2	0	5
NFE2	1	3	0	3	2	1
NFIA	2	2	5	2	1	1
NFIC	3	3	4	5	5	1
NFIC::TLX1	3	2	5	7	3	4
NFIL3	0	0	0	2	0	2
NFIX	3	3	6	3	2	2
NFKB1	3	5	1	1	4	1
NFKB2	2	4	1	0	3	1
NFYA	2	0	2	0	1	2
NFYB	2	0	4	0	1	1
NHLH1	0	9	3	4	6	1
NKX2-3	3	2	2	5	5	3
NKX2-8	3	2	2	3	2	3
NKX3-2	2	0	1	4	3	3
NKX6-1	0	0	0	1	0	1
NKX6-2	0	0	0	1	0	1
NOTO	1	0	0	0	0	1
NR1A4::RXRA	1	2	1	1	3	1
NR1H2::RXRA	3	1	1	3	3	3
NR1H4	4	0	2	1	2	3
NR2C2	1	10	10	6	7	3
NR2F1	0	6	1	1	1	2
NR2F2	1	2	2	1	4	3
NR3C1	3	1	0	0	1	2
NR3C2	2	1	1	0	2	1
NR4A1	1	3	1	1	4	5
NR4A2	2	3	4	6	4	0
NR4A2::RXRA	2	3	3	1	3	2
NRF1	0	12	4	2	2	1
NRL	0	2	0	1	0	6
Neurog1	2	2	1	3	1	6
Nfe2l2	2	2	0	5	4	2

Nkx2-5	2	0	0	3	0	2
Nkx2-5_var.2	4	2	5	5	5	1
Nkx3-1	4	0	2	4	3	3
Nobox	2	0	0	1	0	2
Npas2	3	3	3	2	2	1
Nr1h3::Rxra	3	0	1	2	3	3
Nr2e1	2	0	4	2	4	4
Nr2e3	0	0	0	0	1	4
Nr2f6	2	2	2	2	4	2
Nr2f6_var.2	2	2	2	2	4	1
Nr5a2	2	1	4	1	3	1
OLIG1	0	0	0	2	0	1
OLIG2	2	0	0	3	0	2
OLIG3	2	0	0	2	0	3
ONECUT1	4	0	1	4	0	1
ONECUT2	4	0	2	4	0	0
ONECUT3	4	0	2	6	0	1
OTX1	0	2	0	1	3	3
OTX2	1	2	0	1	4	4
PAX1	1	4	0	0	2	3
PAX3	4	0	0	5	0	0
PAX4	0	0	0	0	0	2
PAX5	1	5	4	3	3	2
PAX7	4	0	0	5	0	0
PAX9	0	4	1	2	2	1
PBX1	4	1	1	0	1	5
PBX2	4	2	1	2	1	0
PBX3	3	4	4	3	1	1
PDX1	0	0	0	0	1	2
PHOX2A	1	0	0	3	0	0
PITX3	0	2	0	1	2	3
PKNOX1	2	3	3	1	0	2
PKNOX2	3	0	3	5	0	3
PLAG1	5	21	6	9	9	2
POU1F1	2	1	0	2	0	2
POU2F1	2	0	0	0	0	2
POU2F2	0	0	2	2	0	4
POU3F1	0	0	0	1	0	1
POU3F2	0	1	0	1	0	2
POU3F3	1	1	0	1	1	1
POU3F4	2	0	1	1	0	1
POU4F1	4	0	0	1	0	4
POU4F2	4	0	0	2	1	5
POU4F3	4	0	1	1	1	5
POU5F1	2	0	2	2	1	2
POU5F1B	3	0	1	2	0	1
POU6F1	0	0	0	2	0	2
POU6F2	2	0	0	0	1	1
PPARA::RXRA	1	1	0	2	5	2
PPARG	0	1	1	1	7	3
PRDM1	4	1	5	1	1	1

PROP1	2	0	0	3	1	0
PROX1	0	1	0	1	2	3
PRRX1	1	0	0	1	0	2
Pax2	2	1	1	1	3	2
Pax6	2	0	0	0	2	2
Phox2b	1	0	0	3	0	0
Pitx1	1	2	0	2	2	3
Pou2f3	1	0	1	1	1	1
Pou5f1::Sox2	6	0	2	3	3	2
Pparg::Rxra	5	7	4	7	8	3
Prrx2	1	0	0	1	0	2
RARA	1	3	2	5	3	1
RARA::RXRA	3	8	8	0	5	2
RARA::RXRG	3	7	3	1	1	5
RARA_var.2	4	6	4	2	2	4
RAX2	1	0	0	1	0	2
RAX	1	0	0	1	0	2
RBPJ	3	1	1	2	3	5
REL	7	0	3	1	2	1
RELA	7	3	2	2	1	1
RELB	7	2	2	1	3	3
REST	6	8	4	6	4	4
RFX2	2	1	0	3	1	5
RFX3	2	0	1	4	0	4
RFX4	2	0	1	1	2	4
RFX5	4	1	2	1	2	0
RHOXF1	0	3	0	4	4	3
RORA	0	2	3	0	2	2
RORA_var.2	1	1	0	4	1	2
RORB	0	2	0	1	1	1
RORC	0	2	1	3	0	0
RREB1	1	7	4	2	1	2
RUNX1	1	4	3	5	3	5
RUNX2	1	4	0	1	1	2
RUNX3	1	3	1	2	1	3
RXRA::VDR	0	1	1	0	1	2
RXRB	2	5	1	2	2	2
RXRG	2	3	1	2	3	3
Rarb	2	4	5	3	4	2
Rarb_var.2	3	4	4	1	2	0
Rarg	2	4	1	5	4	2
Rarg_var.2	2	8	1	3	0	1
Rfx1	2	2	2	7	2	4
Rhox11	1	0	1	0	1	1
Rxra	3	2	3	2	4	2
SCRT1	3	3	2	1	5	1
SCRT2	1	1	4	2	5	2
SHOX	1	0	0	1	0	2
SIX1	1	0	1	1	3	2
SIX2	3	0	2	1	1	3
SMAD2::SMAD3::SMAD4	5	8	9	7	2	4

SMAD3	1	0	1	0	2	1
SNAI2	2	5	7	2	4	0
SOX10	4	0	1	8	3	0
SOX13	1	0	1	2	3	1
SOX15	1	0	2	8	2	0
SOX21	2	0	1	1	3	2
SOX4	5	1	0	0	2	2
SOX8	4	0	1	1	3	1
SOX9	2	0	1	2	1	0
SP1	5	20	14	9	7	6
SP2	4	21	14	8	6	6
SP3	3	17	9	6	4	2
SP4	4	10	5	5	3	0
SP8	2	11	7	3	2	3
SPDEF	2	2	2	4	2	1
SPI1	4	1	1	1	3	2
SPIB	7	1	4	5	2	4
SPIC	4	1	6	2	3	2
SREBF1	2	3	1	6	3	0
SREBF2	3	0	2	8	3	1
SREBF2_var.2	0	5	0	5	3	0
SRF	1	1	0	0	0	1
SRY	2	0	2	5	2	1
STAT1	2	1	1	1	2	1
STAT1::STAT2	8	1	6	8	0	8
STAT3	8	0	1	1	4	5
Shox2	1	0	0	1	0	2
Six3	0	0	2	1	2	3
Smad4	1	2	2	1	1	2
Sox11	0	0	1	2	0	3
Sox17	0	0	1	1	0	1
Sox1	2	1	1	3	1	3
Sox2	4	0	3	2	3	1
Sox3	4	1	6	8	3	2
Sox5	1	0	0	1	0	0
Sox6	2	0	5	8	3	0
Spz1	1	4	6	1	1	3
Srebf1_var.2	2	5	0	3	3	0
Stat4	5	0	5	0	3	4
Stat5a::Stat5b	2	0	4	5	4	4
Stat6	1	0	3	0	3	3
T	4	0	2	3	1	2
TAL1::TCF3	3	5	3	4	0	5
TBP	2	0	1	1	3	4
TBR1	2	1	2	2	3	1
TBX15	2	4	5	4	4	1
TBX19	5	0	1	1	0	0
TBX1	1	2	2	2	1	1
TBX20	0	3	3	6	2	1
TBX21	1	4	2	4	4	1
TBX2	2	2	2	3	2	1

TBX4	2	5	3	4	4	1
TBX5	2	5	6	5	3	1
TCF3	2	15	4	3	6	3
TCF4	0	13	7	4	6	2
TCF7L1	3	0	3	1	2	0
TCF7L2	4	1	4	0	3	1
TEAD1	4	0	1	1	0	6
TEAD2	7	0	1	4	2	5
TEAD3	4	0	1	3	1	4
TEAD4	4	0	0	1	1	5
TEF	0	0	0	0	0	0
TFAP2A	3	11	4	4	4	2
TFAP2A_var.2	5	18	5	7	2	2
TFAP2A_var.3	3	9	4	9	2	1
TFAP2B	6	16	5	6	3	3
TFAP2B_var.2	3	8	5	3	4	2
TFAP2B_var.3	2	11	3	9	2	1
TFAP2C	5	18	6	5	2	3
TFAP2C_var.2	3	9	3	4	4	2
TFAP2C_var.3	2	11	3	8	2	1
TFAP4	0	11	3	4	2	3
TFCP2	0	1	4	2	2	1
TFDP1	2	9	5	4	3	3
TFE3	2	4	1	4	2	0
TFEB	3	5	0	5	3	1
TFEC	1	6	0	7	5	1
TGIF1	1	2	1	1	1	3
TGIF2	2	3	2	2	0	2
THAP1	3	5	6	4	5	2
TP53	2	0	4	1	1	0
TP63	1	2	4	0	0	2
TP73	2	1	4	3	2	2
TWIST1	1	7	2	4	1	3
Tcf12	3	11	5	1	5	2
Tcf21	2	10	6	3	5	4
Tcf7	6	2	2	1	1	0
Tcfl5	2	6	0	2	0	0
Twist2	2	4	4	4	0	2
UNCX	1	0	0	1	0	2
USF1	2	7	4	7	4	3
USF2	3	5	2	11	5	2
VAX1	1	0	0	1	1	1
VAX2	0	0	0	0	1	1
VDR	0	1	2	2	2	3
VENTX	1	0	0	1	1	0
VSX1	1	0	0	2	0	1
VSX2	1	0	0	2	0	1
XBP1	0	1	0	1	0	2
YY1	5	4	3	3	6	3
YY2	3	3	1	2	3	2
ZBED1	0	0	0	2	4	3

ZBTB18	2	7	1	3	4	5
ZBTB33	1	3	1	2	1	0
ZBTB7A	2	5	7	5	3	5
ZBTB7B	1	11	1	2	4	2
ZBTB7C	2	10	2	3	3	3
ZEB1	1	10	7	4	5	2
ZIC1	1	22	3	7	4	4
ZIC3	1	13	4	3	5	2
ZIC4	2	23	5	8	4	3
ZNF143	3	4	4	3	5	5
ZNF24	1	1	4	2	1	2
ZNF263	12	12	14	4	5	5
ZNF282	0	1	3	1	2	2
ZNF354C	6	4	3	3	4	2
ZNF384	2	0	4	5	4	2
ZNF410	2	0	0	1	2	2
ZNF740	2	16	7	4	4	0
ZSCAN4	1	0	2	4	2	2
Zfx	1	10	13	12	10	5
Znf423	2	7	3	4	2	5
mix-a	1	0	0	0	0	2
