# Supplementary material for: Tryptophan metabolic gatekeeping in epithelial repair: GPR35-KLF5 circuitry decodes mucosal damage signals for repair programming
Source: Cell Death Dis. 2026 Jan 9;17(1):25. doi: 10.1038/s41419-025-08237-0 (PMC12789062; doi:10.1038/s41419-025-08237-0)
Supplement: Supplementary file 3 — Supplementary appendix 2 [file 41419_2025_8237_MOESM3_ESM.pdf]

Data S2. Clinical data of the ten patient donors

No.	Gender	Age (years)	Diagnosis	Smoking history	Drinking history	Family history of colorectal cancer	Parenteral manifestations	Clinical type	Severity degree	Involved scope	Mayo score	Hemoglobin (g/L)	Albumin (g/L)	ESR	CRP (mg/L)
1	Male	33	UC	No	No	No	No	Chronic relapsing type	Severe activity period	E2	12	52	36.6	5	< 5
2	Female	50	UC	No	No	No	No	Chronic relapsing type	Moderately active phase	E2	9	125	40.6	30	4.2
3	Male	59	UC	No	Yes	No	No	Chronic relapsing type	Moderately active phase	E2	7	144	37.8	10	5.6
4	Female	35	UC	No	No	No	No	Chronic relapsing type	Moderately active phase	E3	8	114	41.6	26	7.2
5	Male	26	UC	Yes	No	No	No	Chronic relapsing type	Severe activity period	E3	11	142	36.9	21	19.7
6	Female	44	UC	No	No	No	No	Chronic relapsing type	Moderately active phase	E2	8	115	37.5	6	1.2
7	Female	43	UC	No	No	No	No	Chronic relapsing type	Moderately active phase	E2	6	134	39	6	0.15
8	Male	55	UC	No	No	No	No	Chronic relapsing type	Moderately active phase	E3	4	155	38.2	1	0.47
9	Male	34	UC	No	No	No	No	Chronic relapsing type	Moderately active phase	E3	5	133	38	28	3.48
10	Male	53	UC	No	No	No	No	Chronic relapsing type	Moderately active phase	E2	4	145	36.4	7	1.16
