# Supplementary material for: Tryptophan metabolic gatekeeping in epithelial repair: GPR35-KLF5 circuitry decodes mucosal damage signals for repair programming
Source: Cell Death Dis. 2026 Jan 9;17(1):25. doi: 10.1038/s41419-025-08237-0 (PMC12789062; doi:10.1038/s41419-025-08237-0)
Supplement: Supplementary file 4 — Supplementary appendix 3 [file 41419_2025_8237_MOESM4_ESM.pdf]

Data S3. Original data files_original western blots

Order number	Cell or tissue	Protein	Figures
Figure 1G	CCD841 CoN	EGF	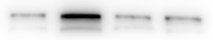
		TFF3	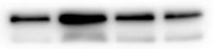

		FGF7	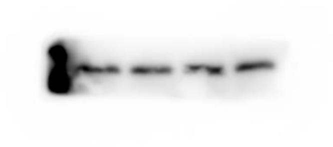
		FGF11	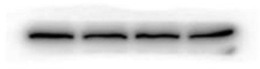

		TGF-β1	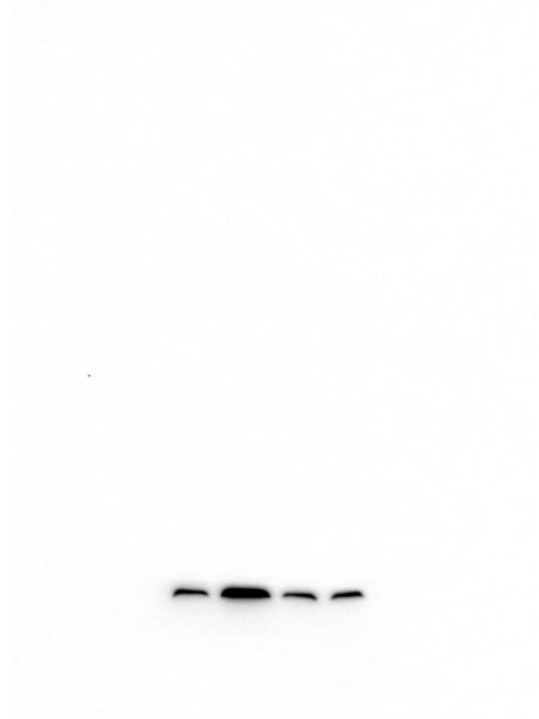
		TGF-β3	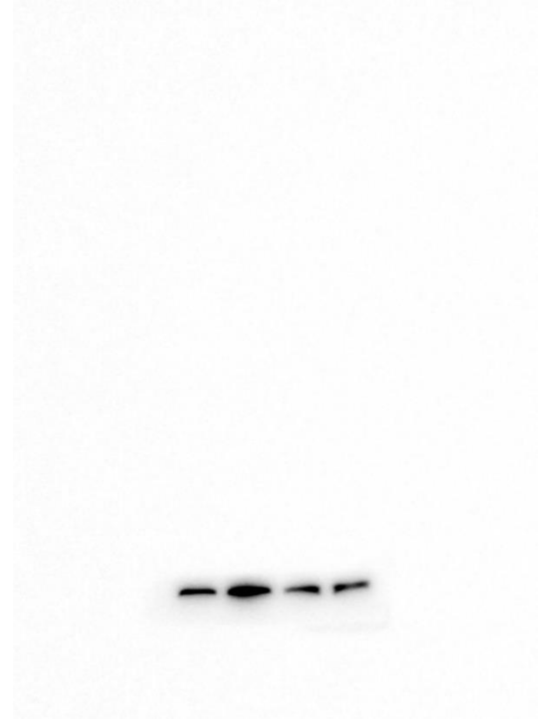

		MMP1	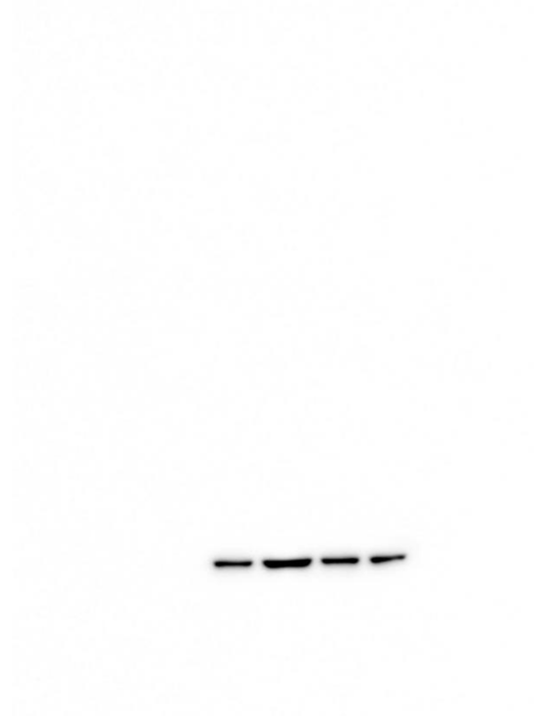
		MMP7	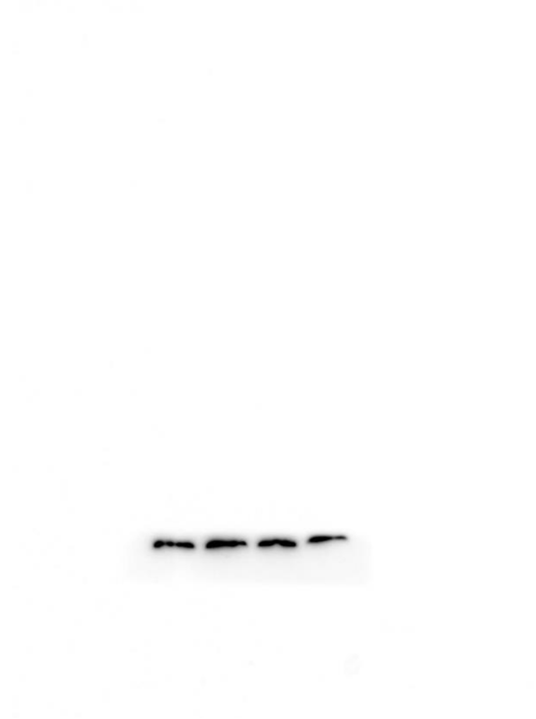

		MMP13	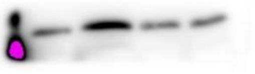
		β-Actin	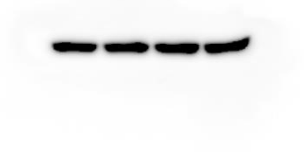

	FHC	EGF	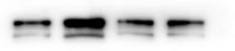
		TFF3	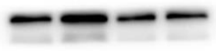

		FGF7	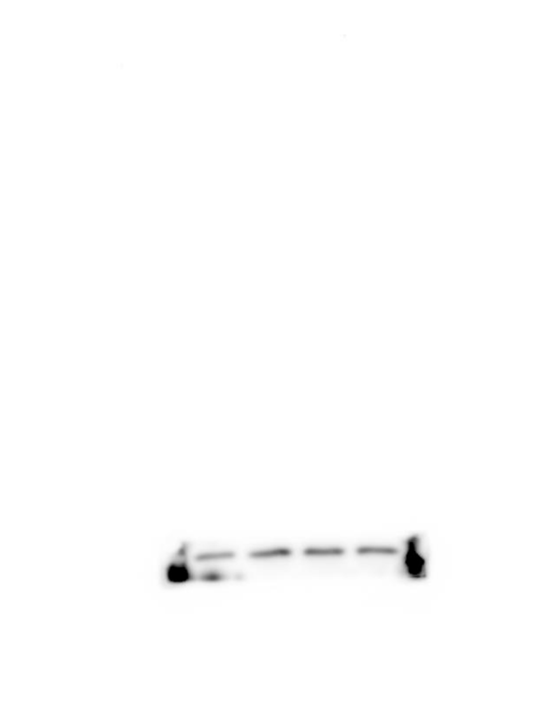
		FGF11	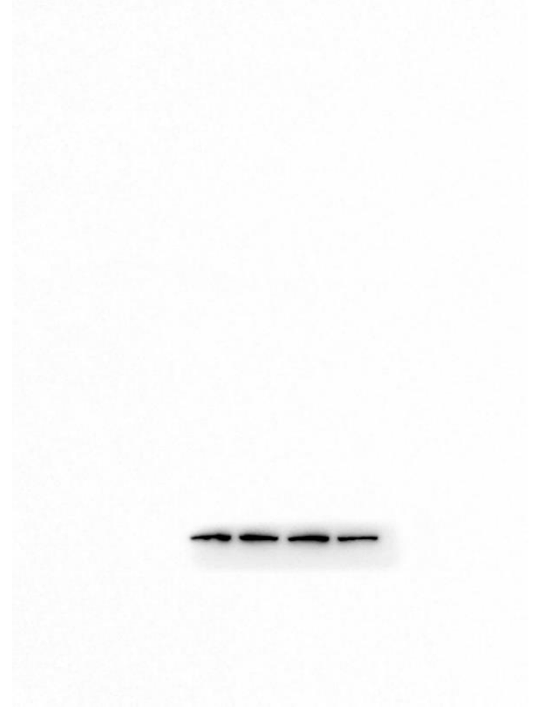

		TGF-β1	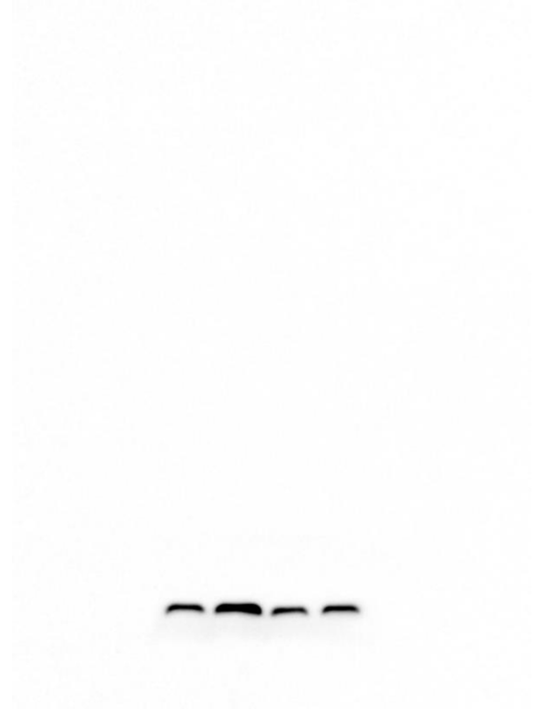
		TGF-β3	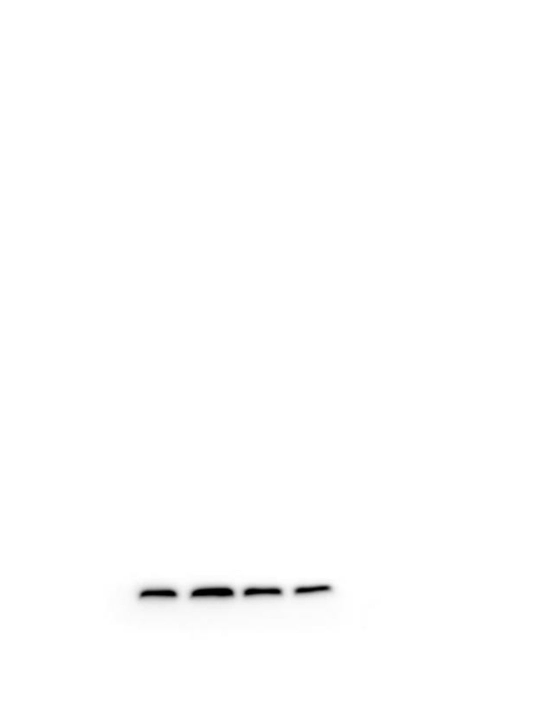

		MMP1	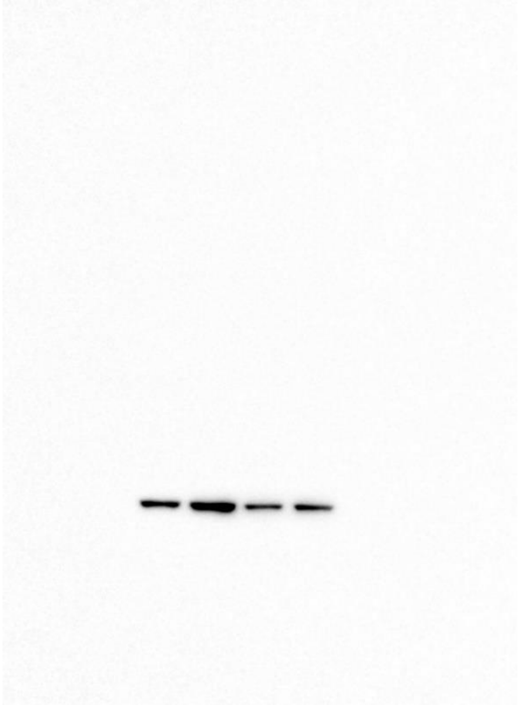
		MMP7	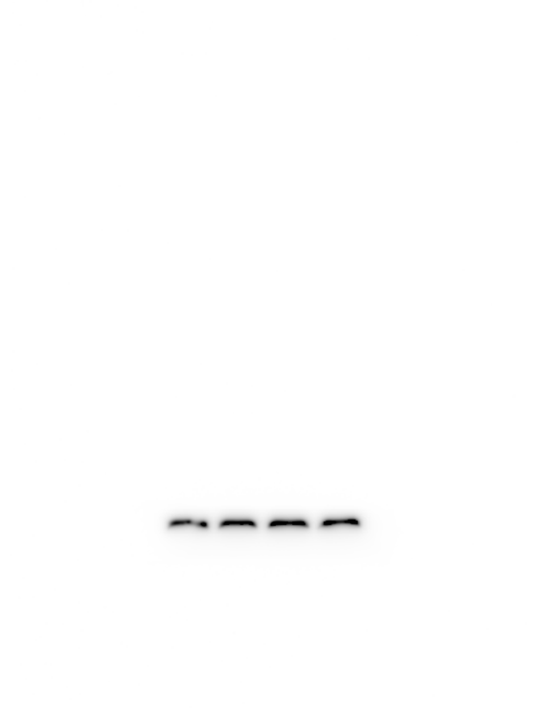

		MMP13	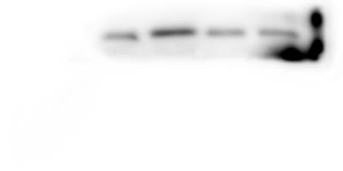
		β-Actin	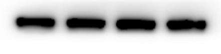

<table>
<tr><td rowspan="2">Figure 1M</td><td rowspan="2">CCD841 CoN</td><td>KLF5</td><td>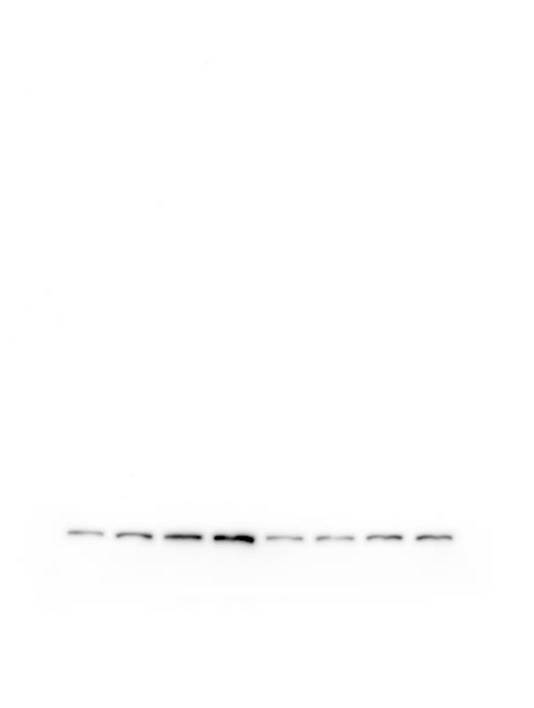</td></tr>
<tr><td>β-Actin</td><td>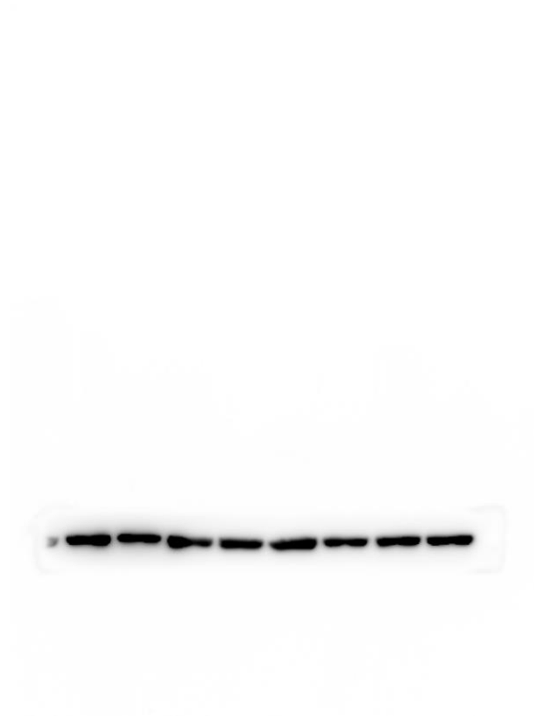</td></tr>
</table>

	FHC	KLF5	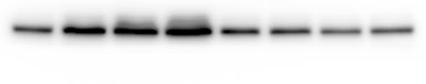
		β-Actin	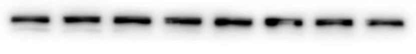

<table>
<tr><td rowspan="2">Figure 2A</td><td rowspan="2">CCD841 CoN</td><td>EGF</td><td>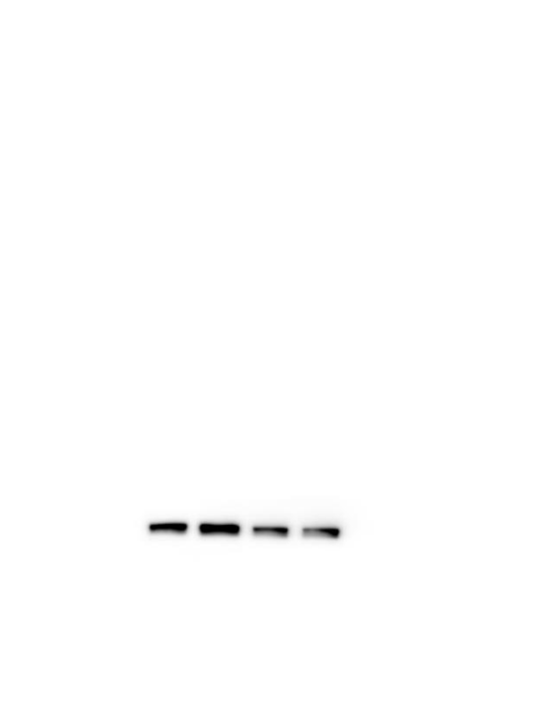</td></tr>
<tr><td>TFF3</td><td>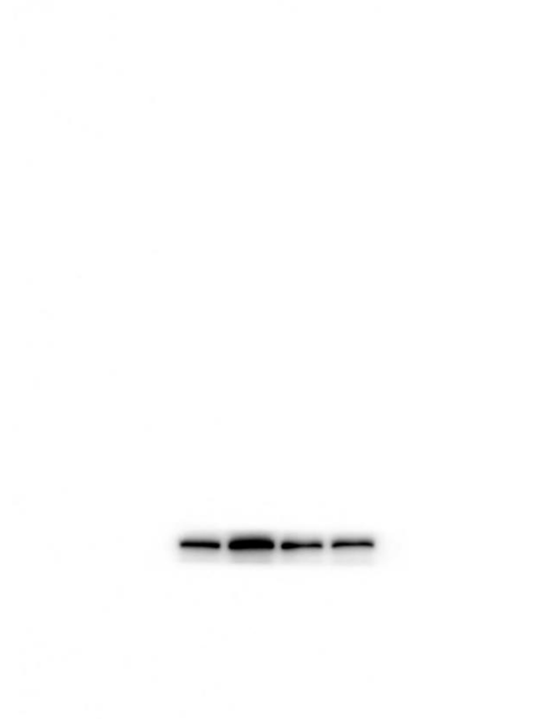</td></tr>
</table>

		TGF-β1	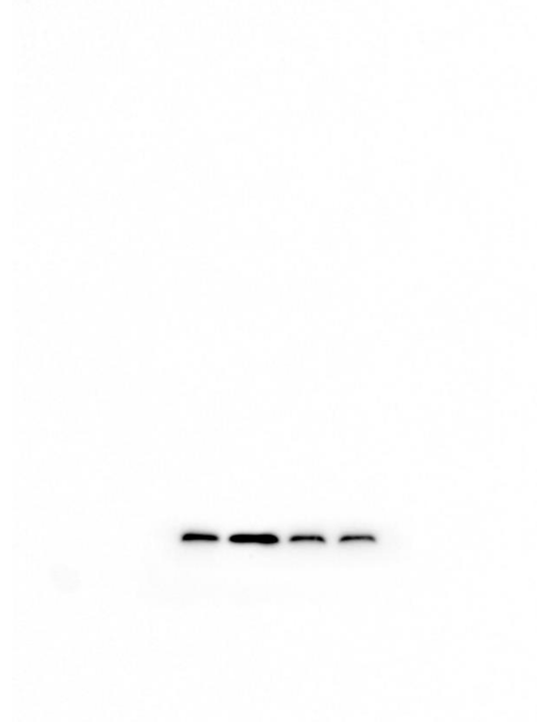
		TGF-β3	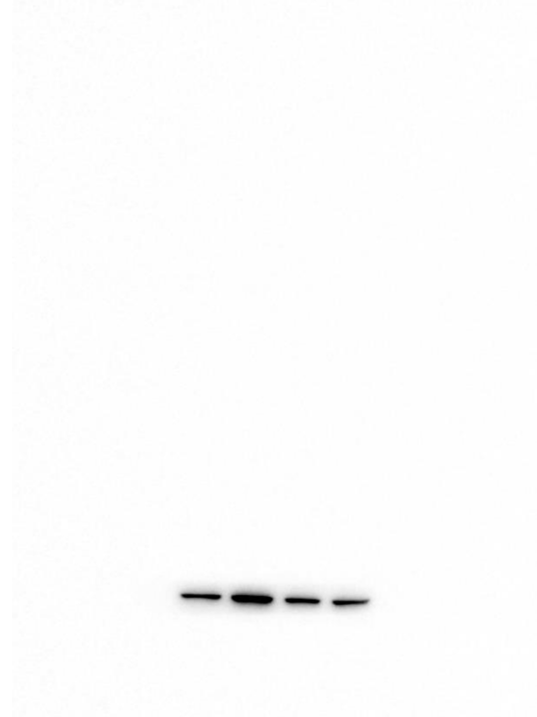

		MMP1	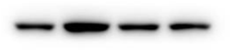
		MMP13	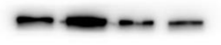

		β-Actin	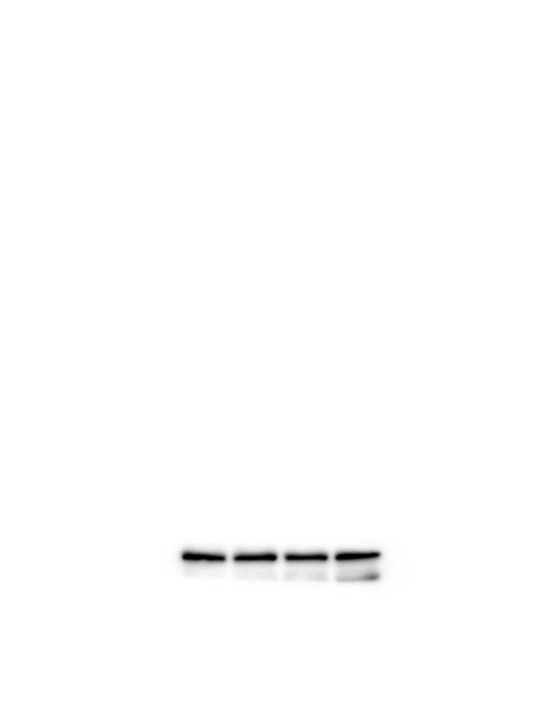
	FHC	EGF	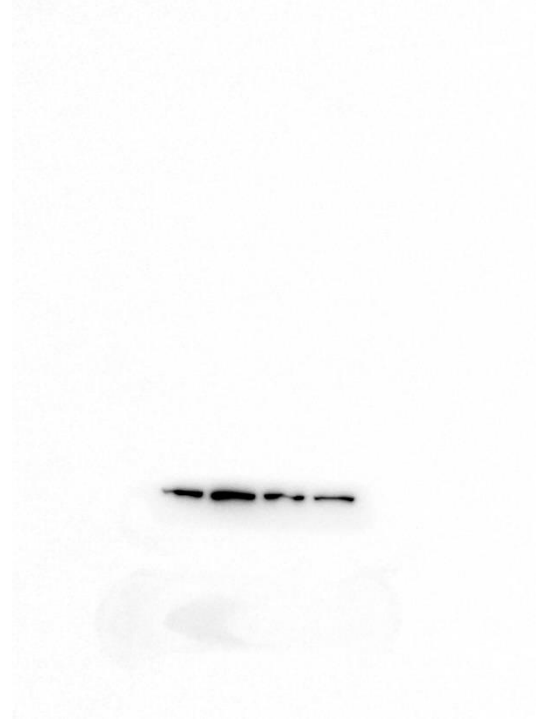

		TFF3	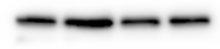
		TGF-β1	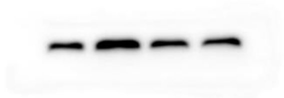

		TGF-β3	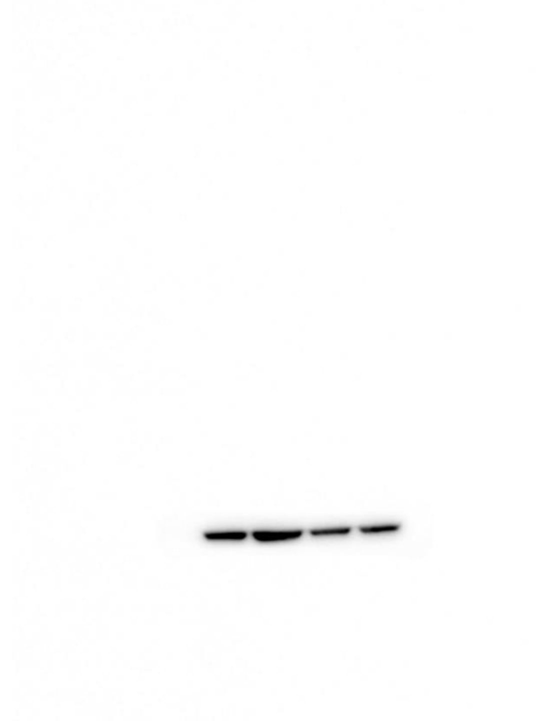
		MMP1	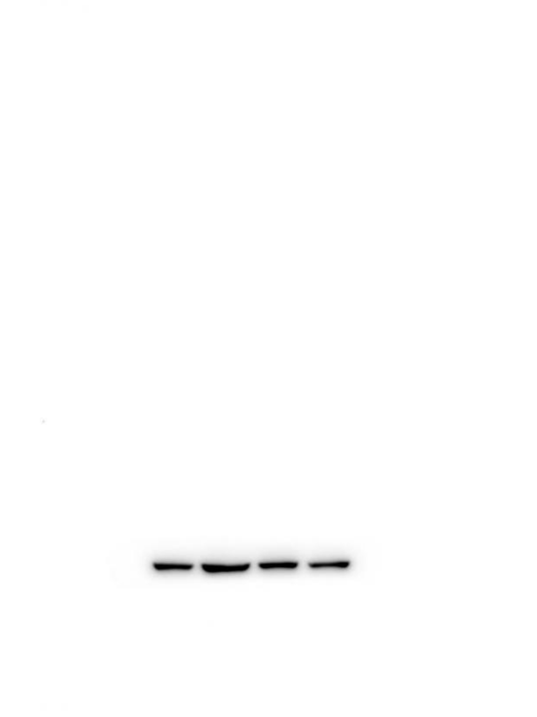

		MMP13	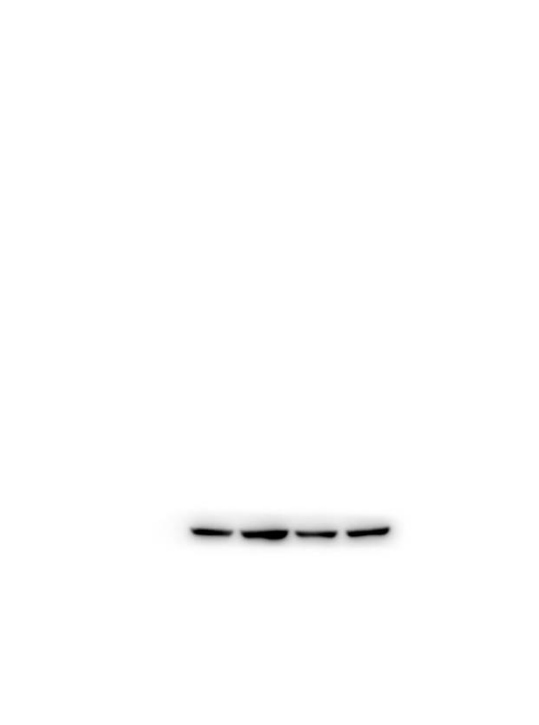
		β-Actin	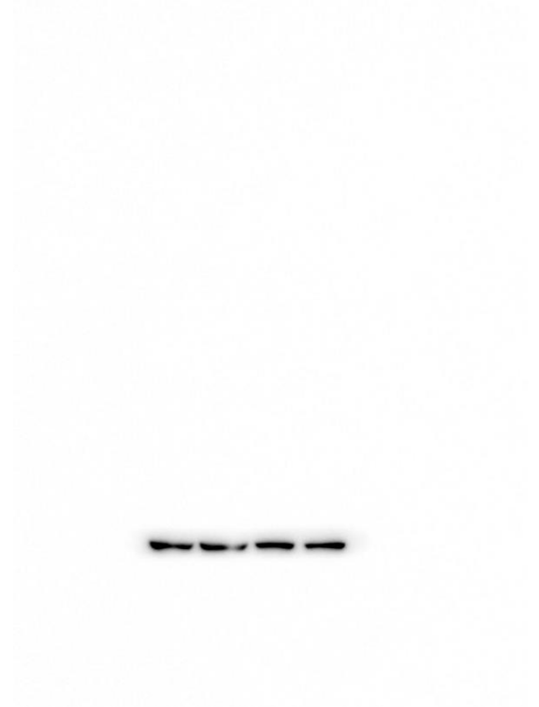

Figure 2D	CCD841 CoN	KLF5	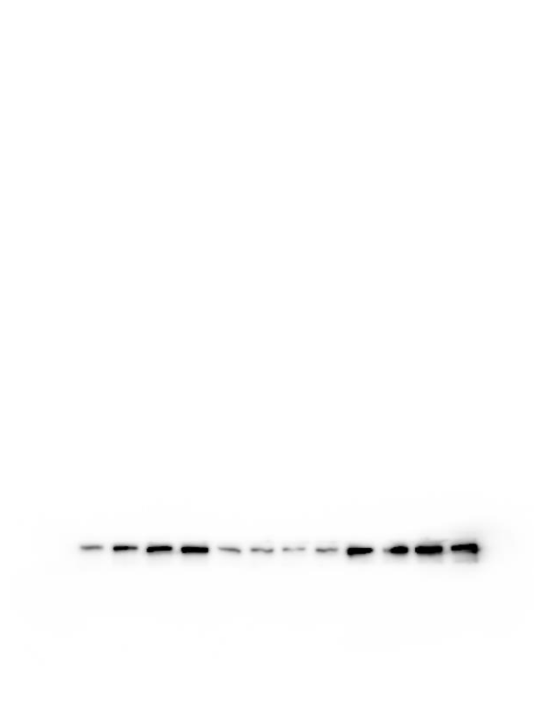
		β-Actin	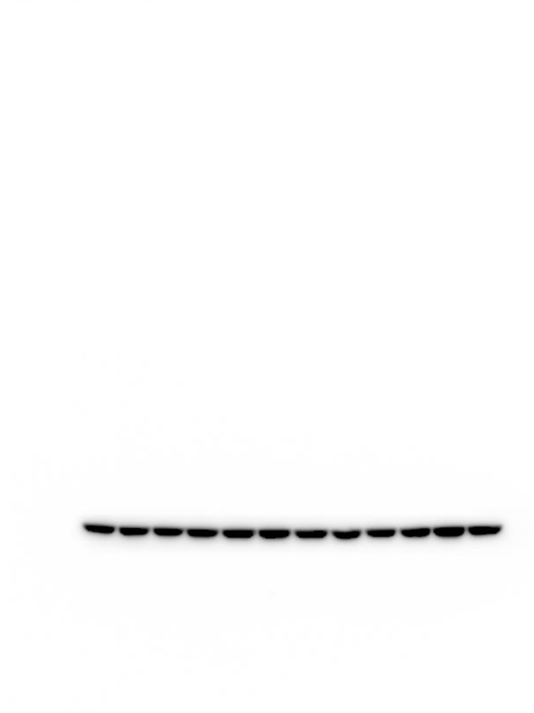

	FHC	KLF5	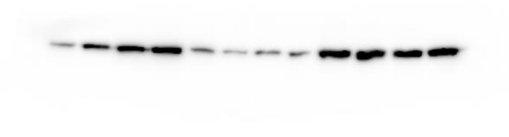
		β-Actin	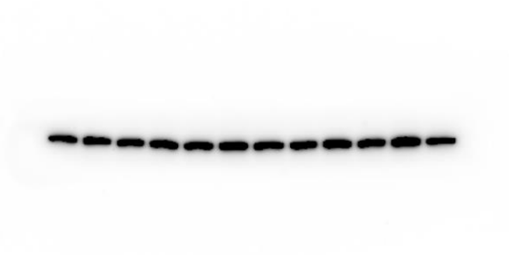

<table>
<tr><td rowspan="2">Figure 3B</td><td rowspan="2">CCD841 CoN</td><td>p-PI3K</td><td>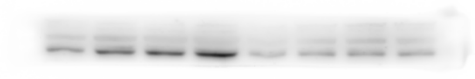</td></tr>
<tr><td>p-AKT</td><td>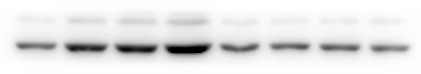</td></tr>
</table>

		p-mTOR	
		β-Actin	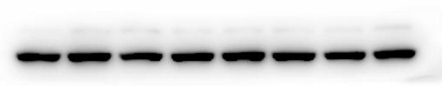

		p-MEK1/2	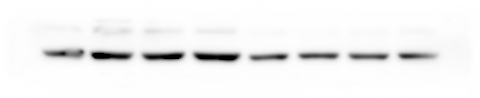
		p-ERK1/2	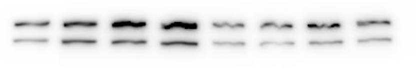

		β-Actin	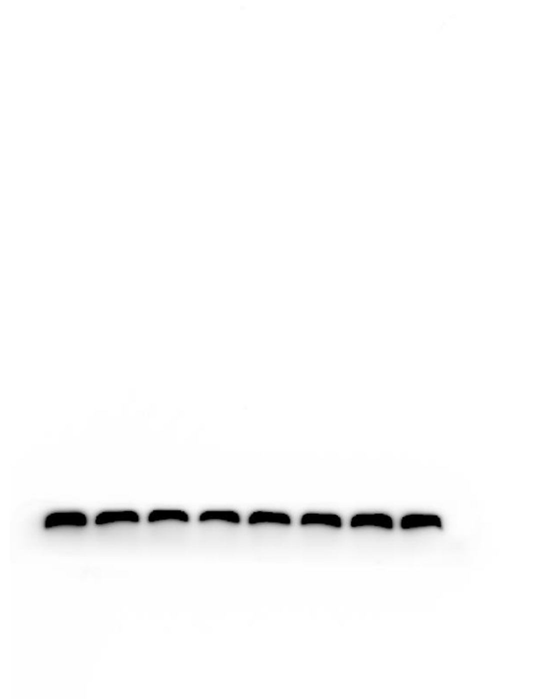
	FHC	p-PI3K	

		p-AKT	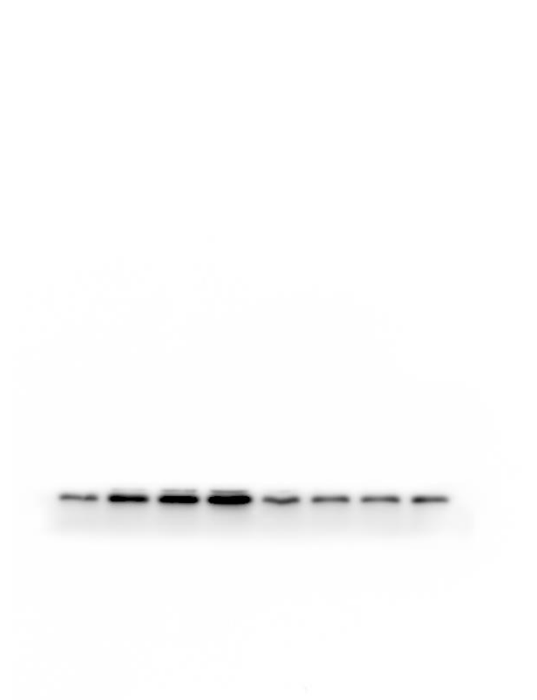
		p-mTOR	

		β-Actin	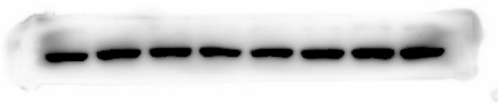
		p-MEK1/2	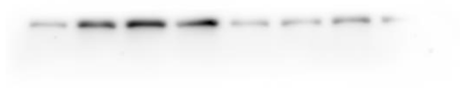

		p-ERK1/2	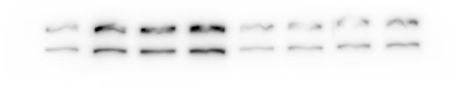
		β-Actin	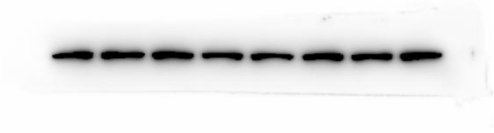

<table>
<tr><td rowspan="2">Figure 3C</td><td rowspan="2">CCD841 CoN</td><td>p-PI3K</td><td></td></tr>
<tr><td>p-AKT</td><td>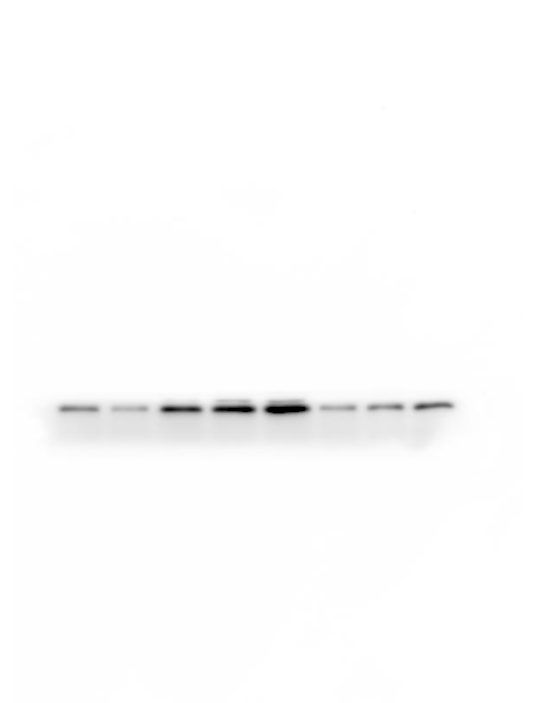</td></tr>
</table>

		p-mTOR	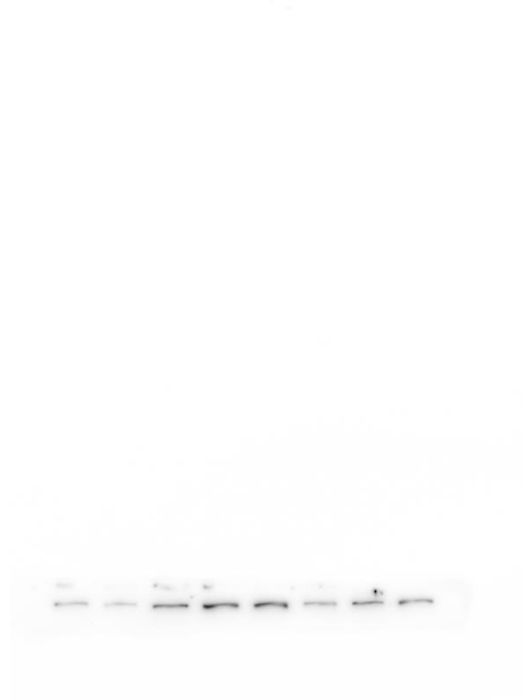
		KLF5	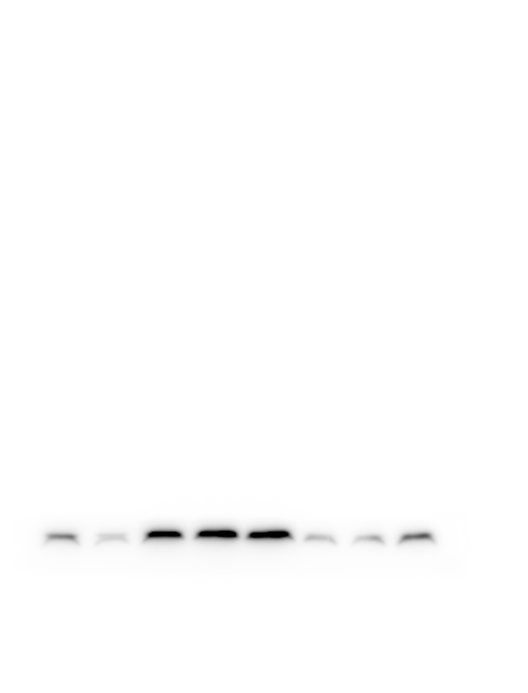

		β-Actin	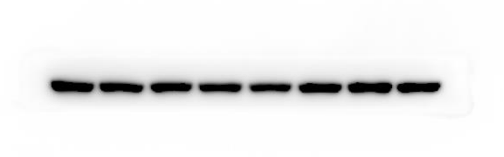
	FHC	p-PI3K	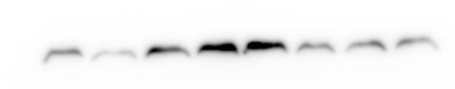

		p-AKT	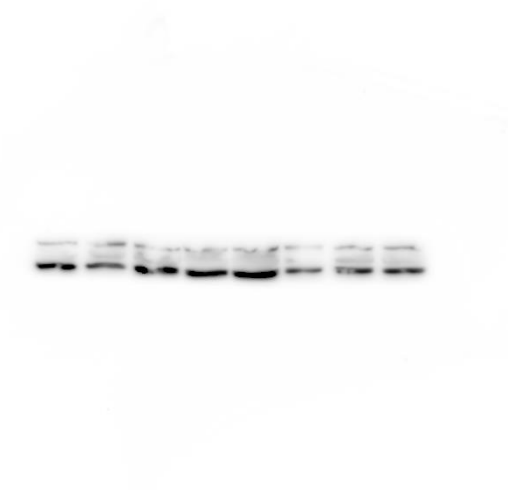
		p-mTOR	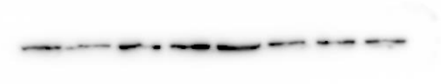

		KLF5	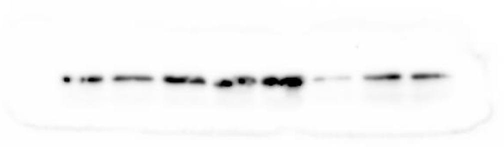
		β-Actin	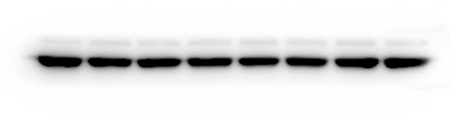

<table>
<tr><td rowspan="2">Figure 3E (Left)</td><td rowspan="2">CCD841 CoN</td><td>p-PI3K</td><td></td></tr>
<tr><td>p-AKT</td><td>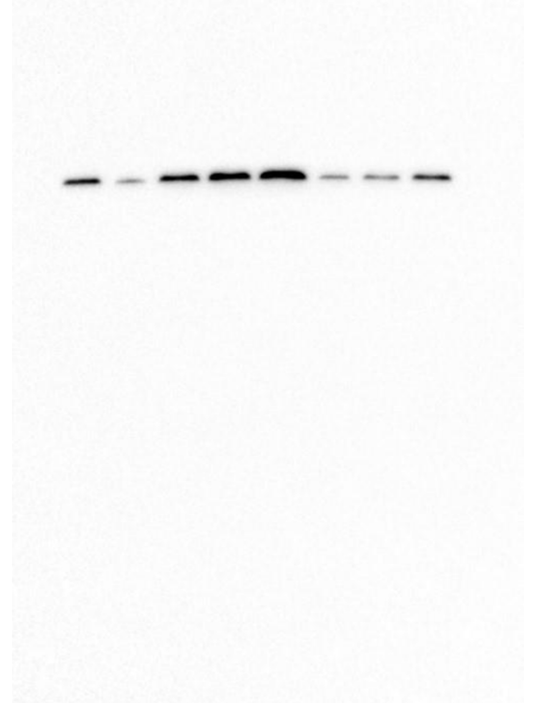</td></tr>
</table>

		p-mTOR	
		KLF5	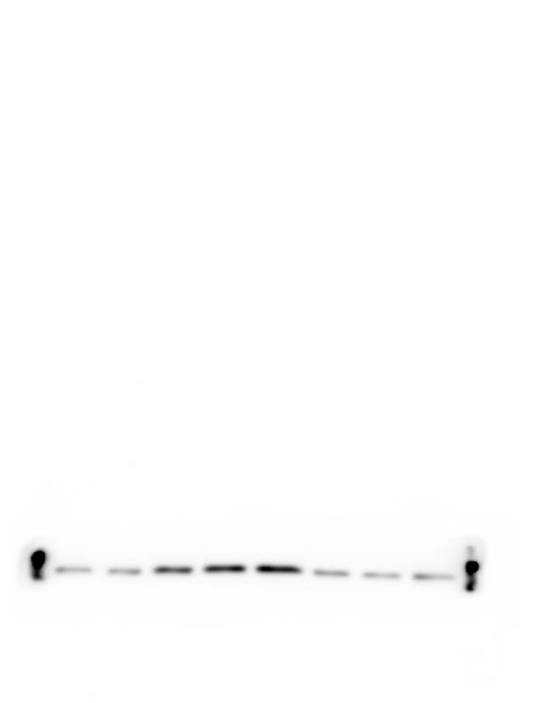

		β-Actin	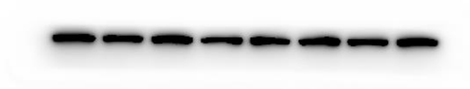
	FHC	p-PI3K	

		p-AKT	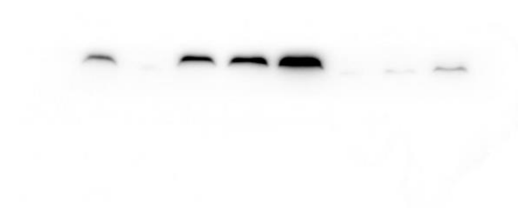
		p-mTOR	

		KLF5	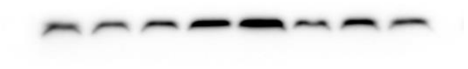
		β-Actin	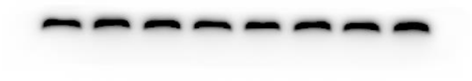

Figure 3E (Right)	CCD841 CoN	p-PI3K	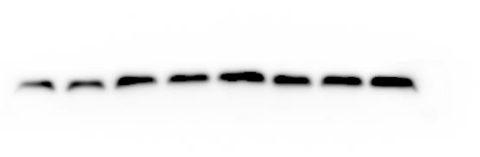
		p-AKT	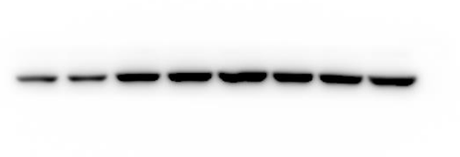

		p-mTOR	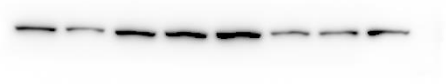
		KLF5	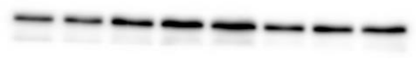

		β-Actin	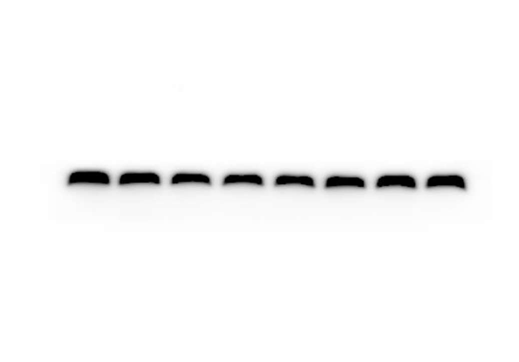
	FHC	p-PI3K	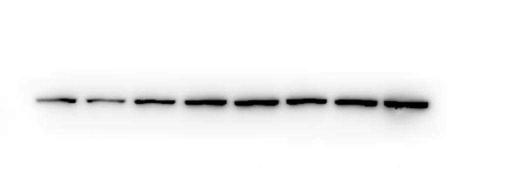

		p-AKT	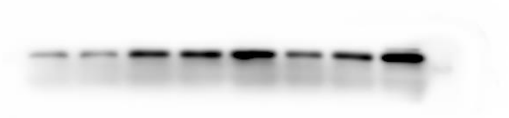
		p-mTOR	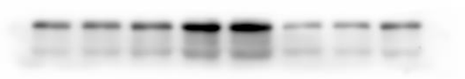

		KLF5	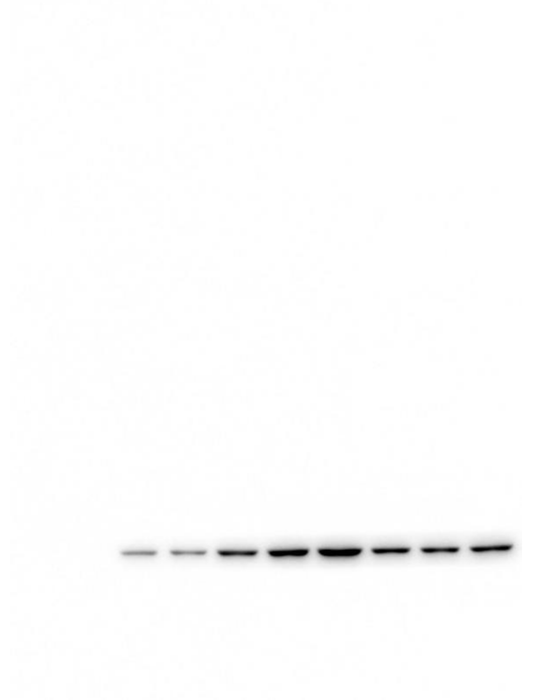
		β-Actin	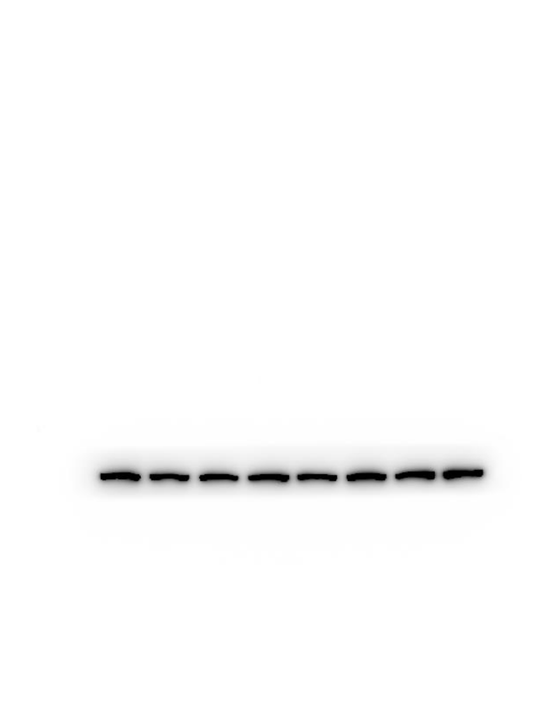

<table>
<tr><td rowspan="2">Figure 4A</td><td rowspan="2">CCD841 CoN</td><td>p-PI3K</td><td>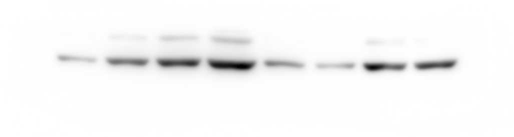</td></tr>
<tr><td>p-AKT</td><td>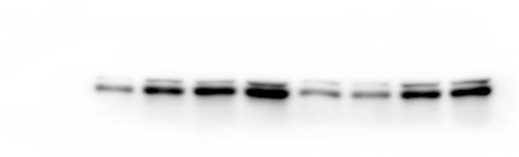</td></tr>
</table>

		p-mTOR	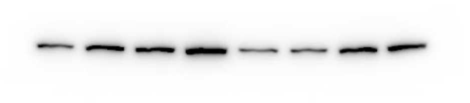
		KLF5	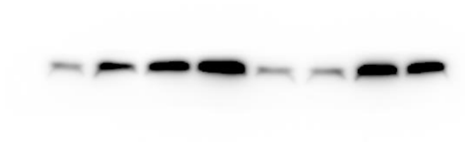

		β-Actin	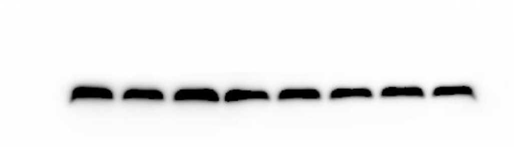
	FHC	p-PI3K	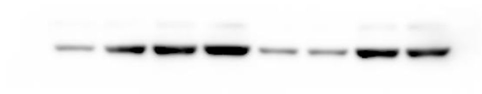

		p-AKT	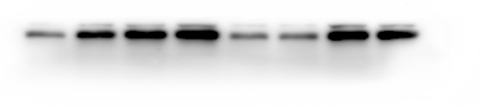
		p-mTOR	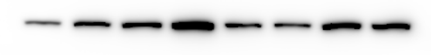

		KLF5	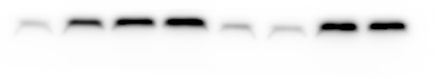
		β-Actin	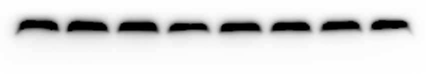

Figure 4G	CCD841 CoN	p-PI3K	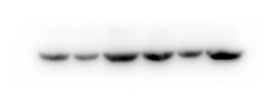
		p-AKT	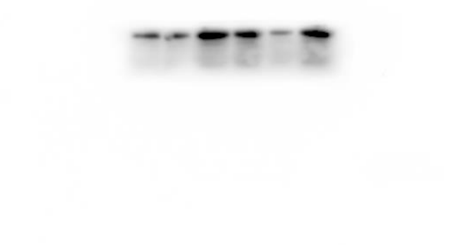

		p-mTOR	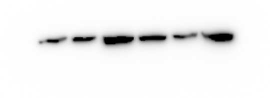
		β-Actin	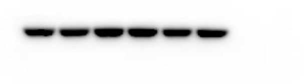

		KLF5	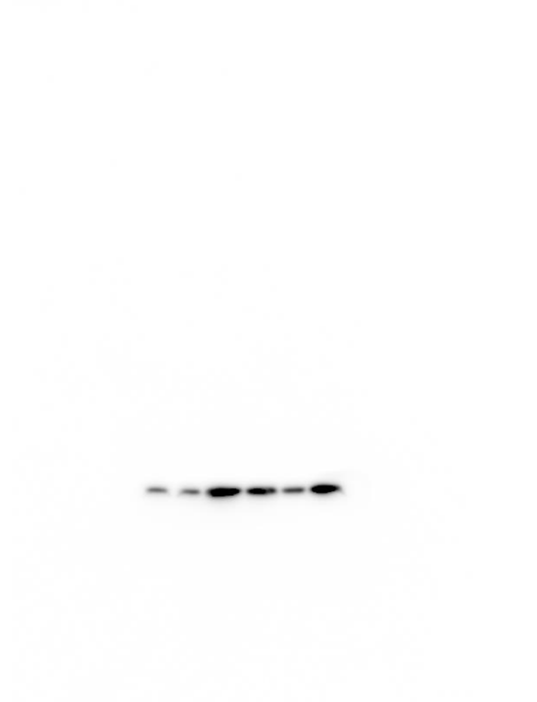
		β-Actin	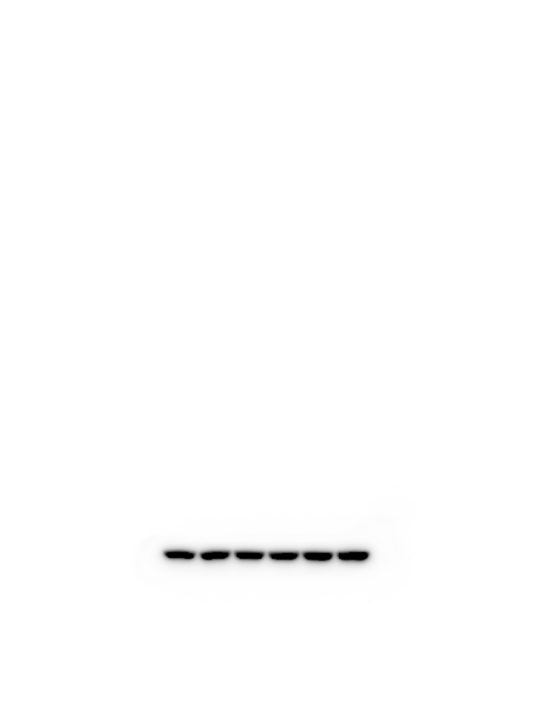

	FHC	p-PI3K	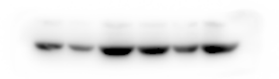
		p-AKT	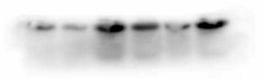

		p-mTOR	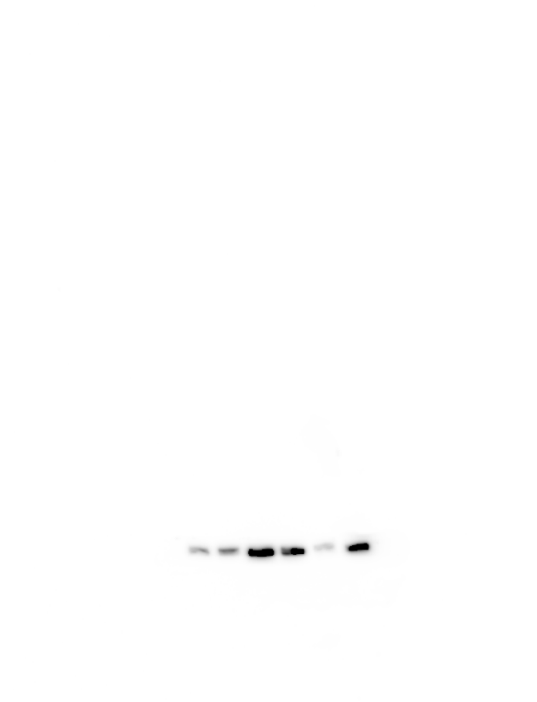
		β-Actin	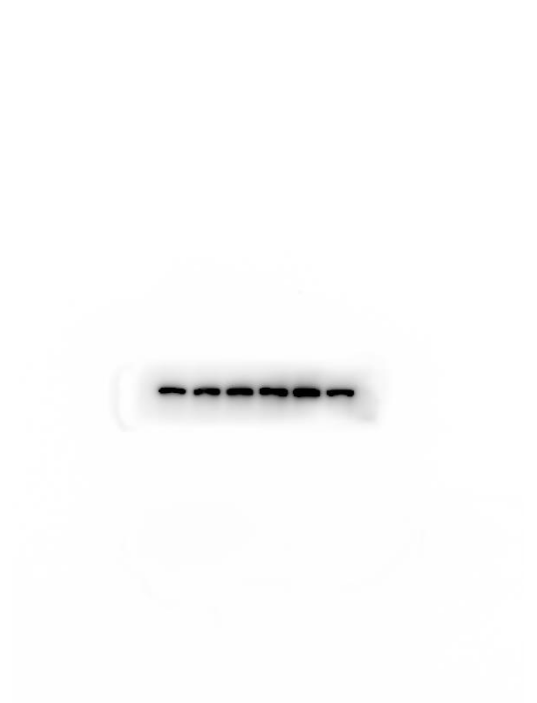

		KLF5	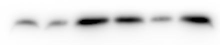
		β-Actin	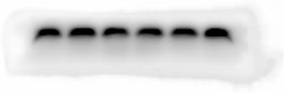

<table>
<tr><td rowspan="2">Figure 4K</td><td rowspan="2">CCD841 CoN</td><td>p-PI3K</td><td></td></tr>
<tr><td>p-AKT</td><td>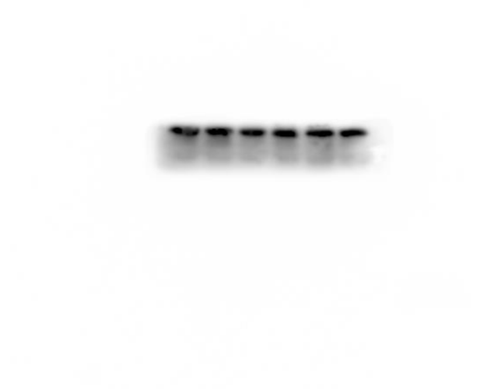</td></tr>
</table>

		p-mTOR	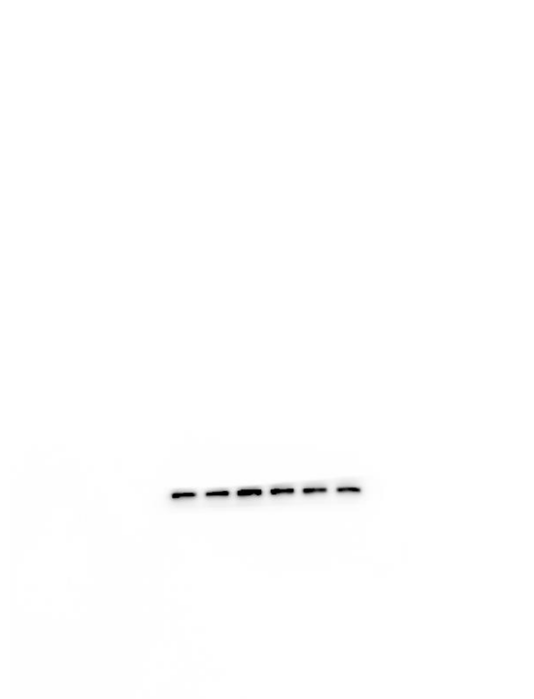
		KLF5	

		β-Actin	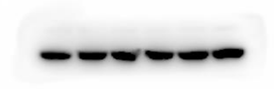
	FHC	p-PI3K	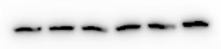

		p-AKT	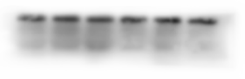
		p-mTOR	

		KLF5	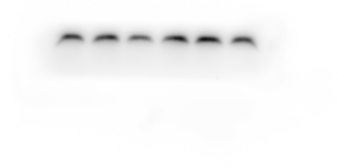
		β-Actin	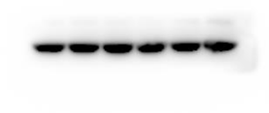

Figure 5D	CCD841 CoN	p-ERK1/2	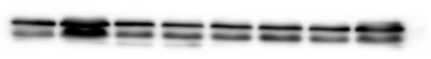
		ERK1/2	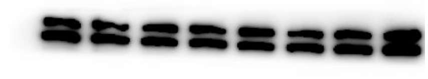

		GPR35	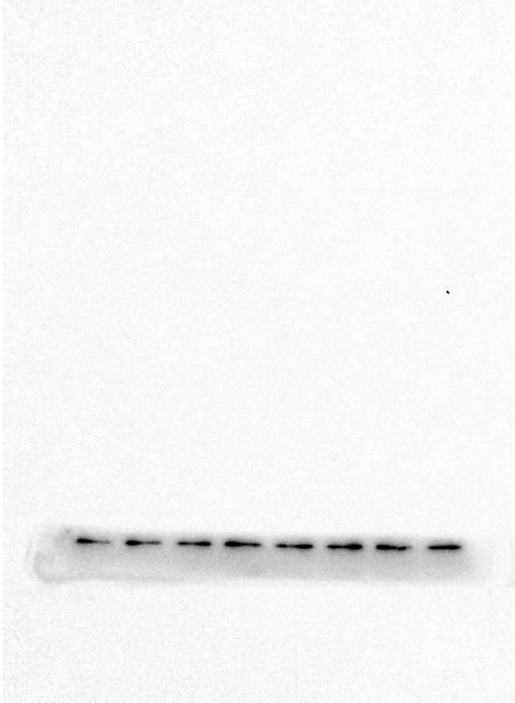
		β-Actin	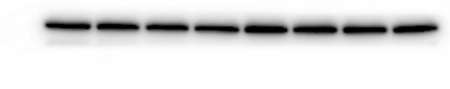

Figure 5E	CCD841 CoN	p-PI3K	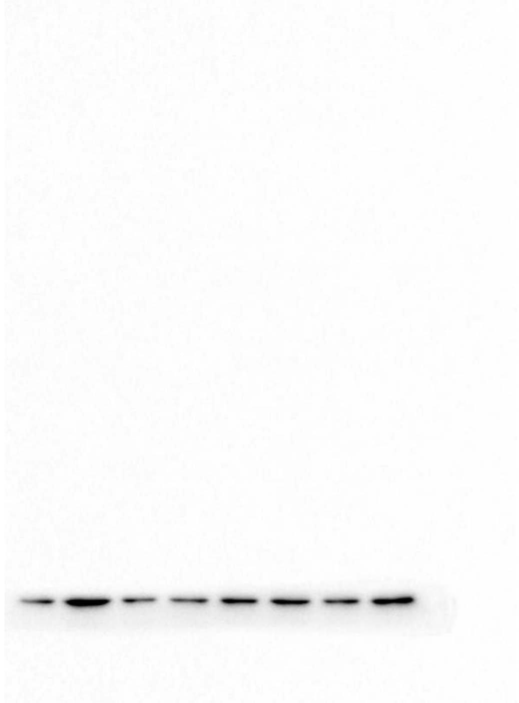
		p-AKT	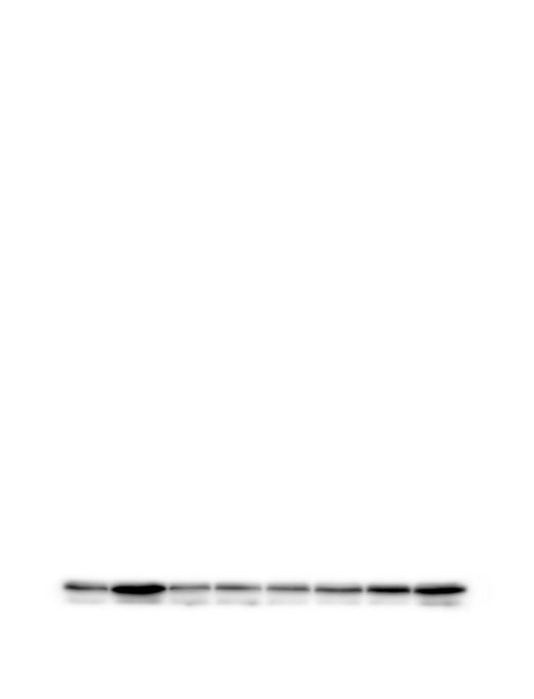

		p-mTOR	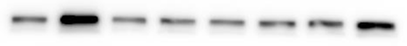
		KLF5	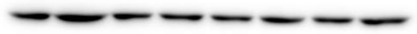

		β-Actin	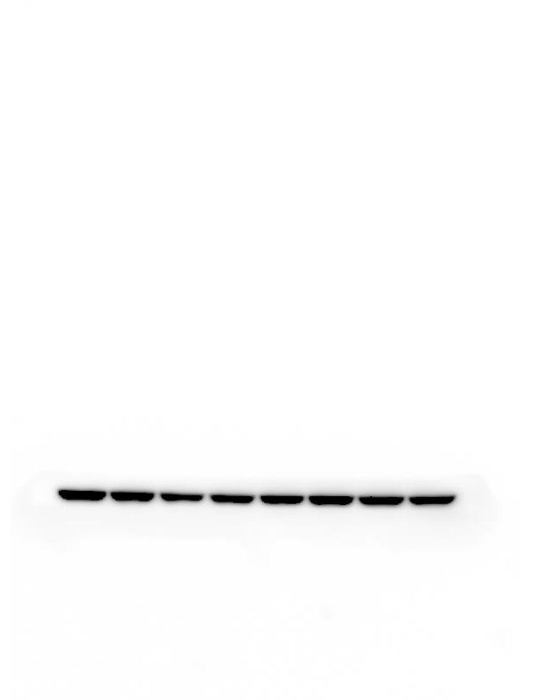
Figure 5I	CCD841 CoN R151A	p-PI3K	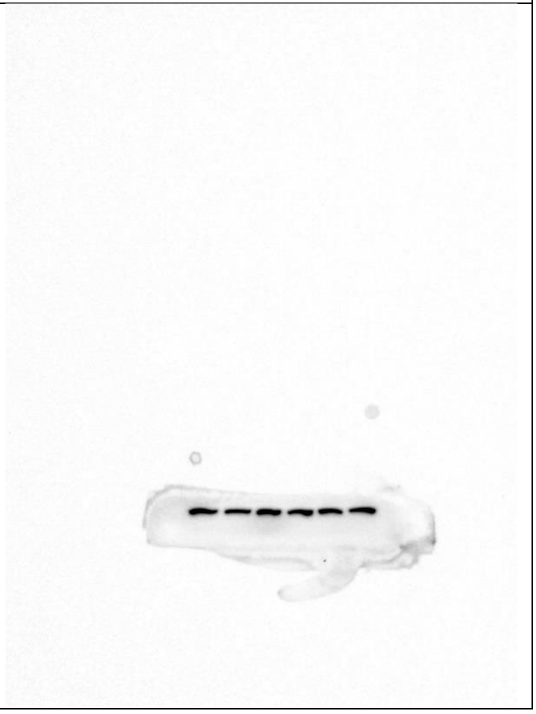

		p-AKT	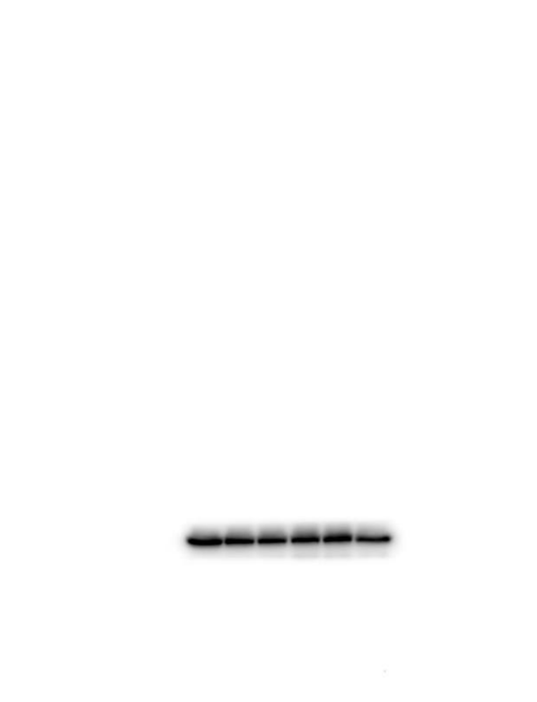
		p-mTOR	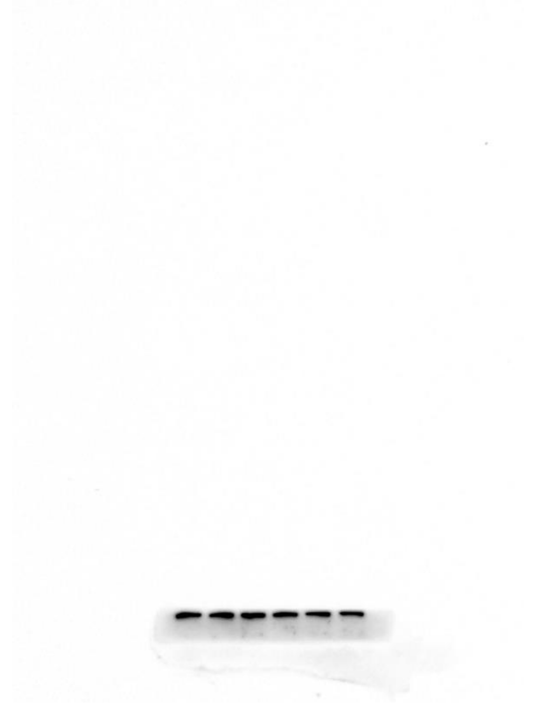

		KLF5	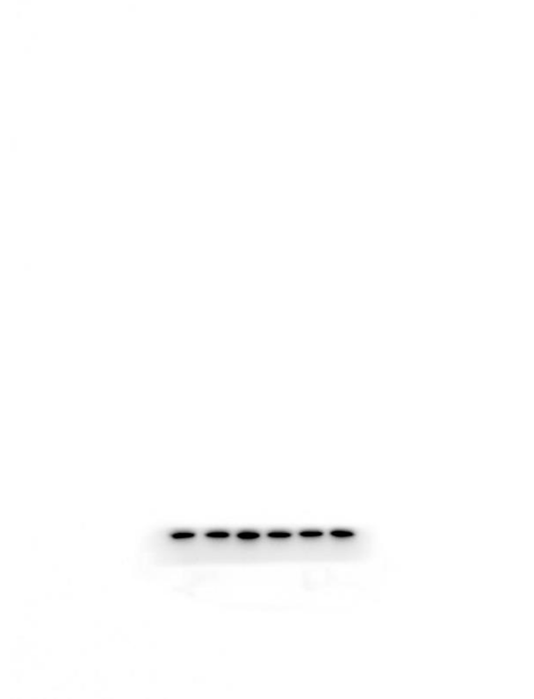
		β-Actin	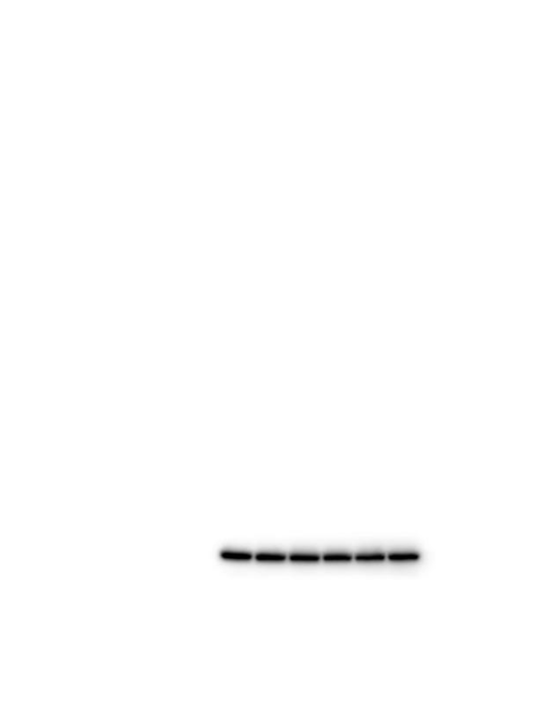

	FHC R151A	p-PI3K	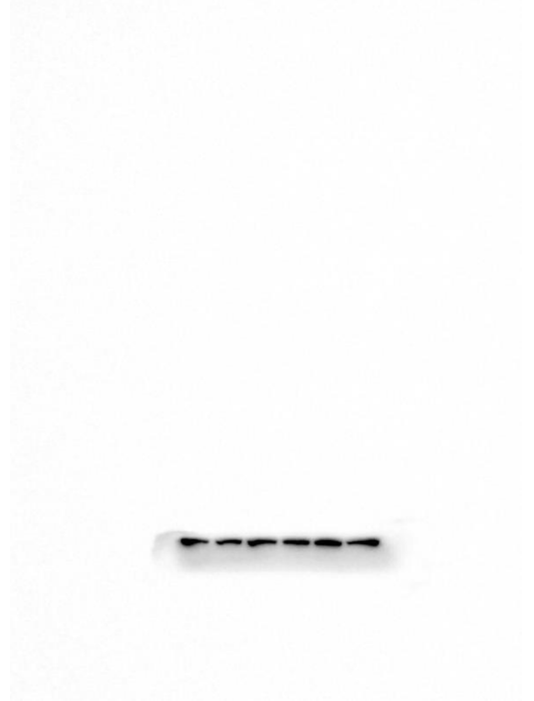
		p-AKT	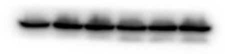

		p-mTOR	
		KLF5	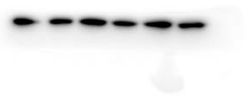

		β-Actin	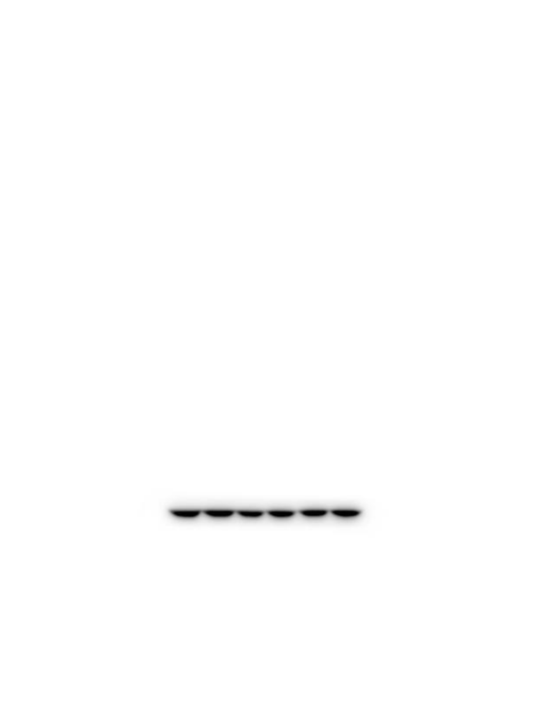
	CCD841 CoN H168A	p-PI3K	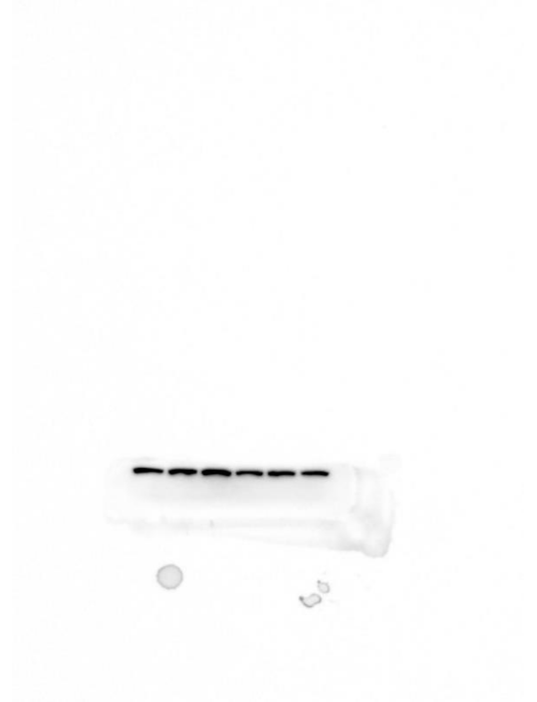

		p-AKT	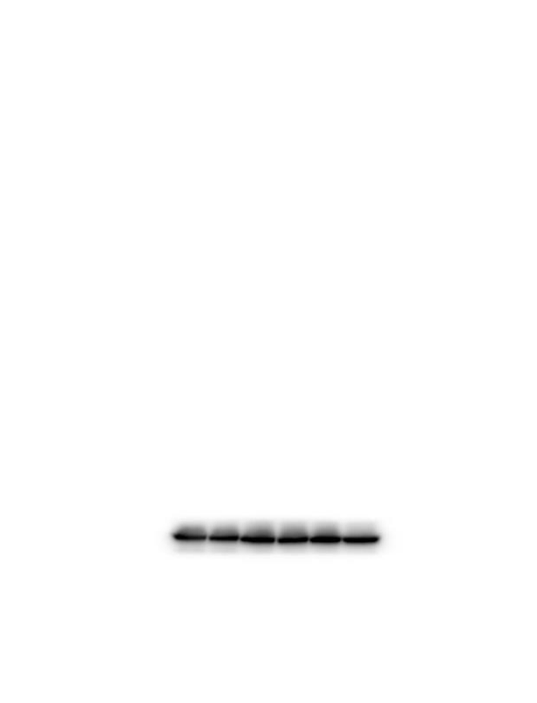
		p-mTOR	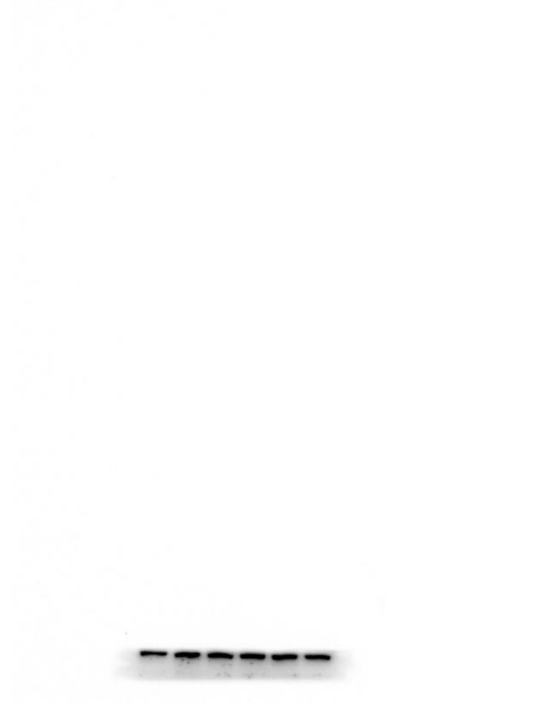

		KLF5	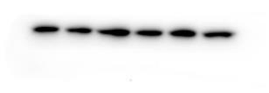
		β-Actin	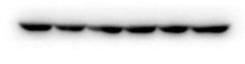

	FHC H168A	p-PI3K	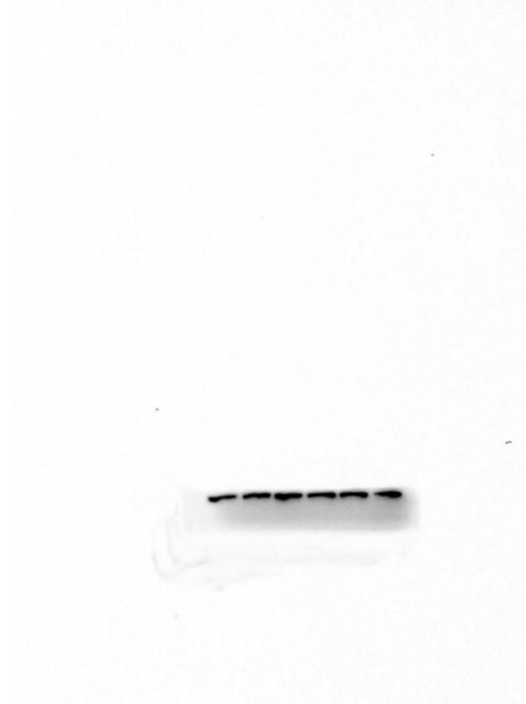
		p-AKT	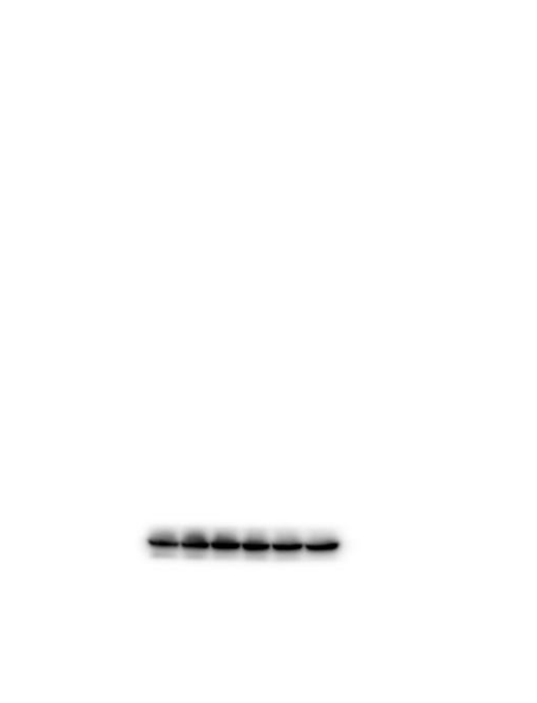

		p-mTOR	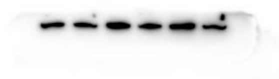
		KLF5	

		β-Actin	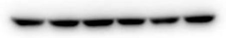
Figure 6H	Colon (Day 10)	p-ERK1/2	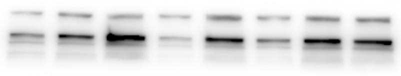

		ERK1/2	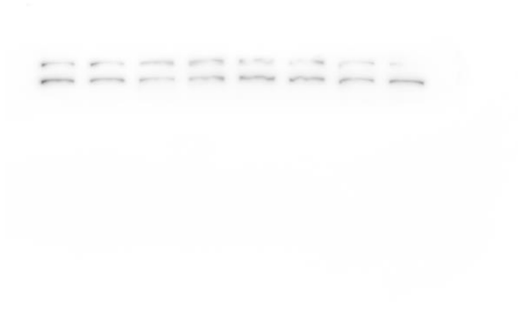
		β-Actin	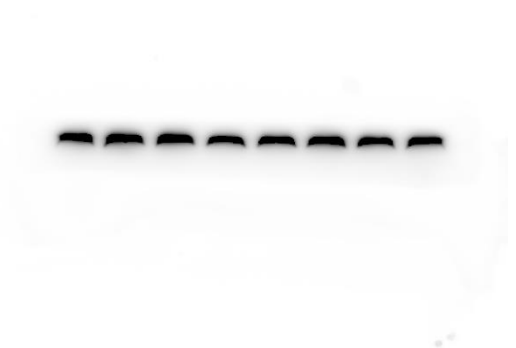

	Colon (Day 17)	p-ERK1/2	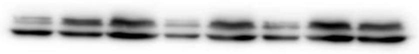
		ERK1/2	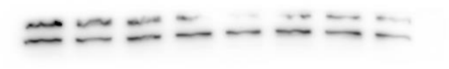

		β-Actin	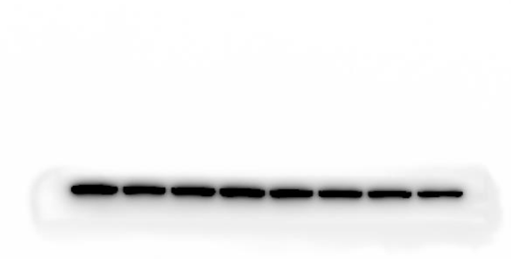
	Colon (Day 24)	p-ERK1/2	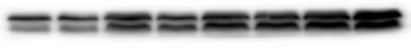

		ERK1/2	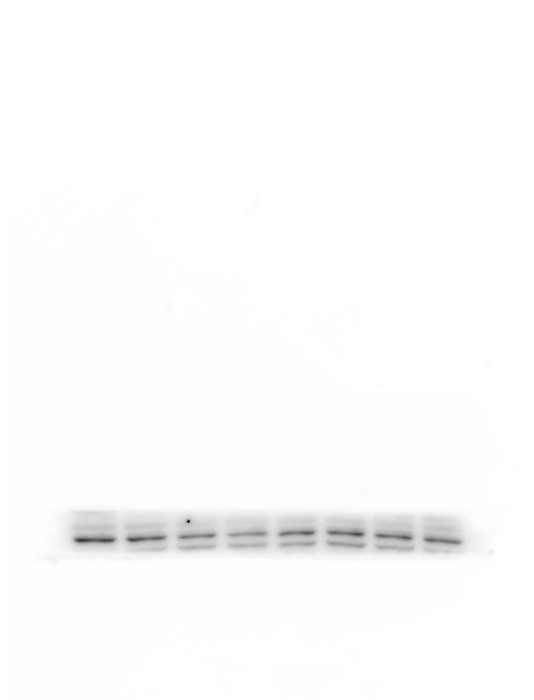
		β-Actin	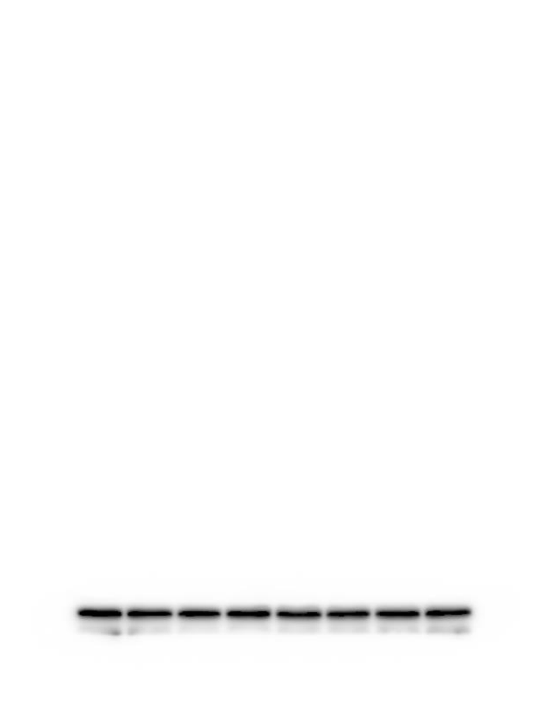

Figure 6I	Colon (Day 10)	p-PI3K	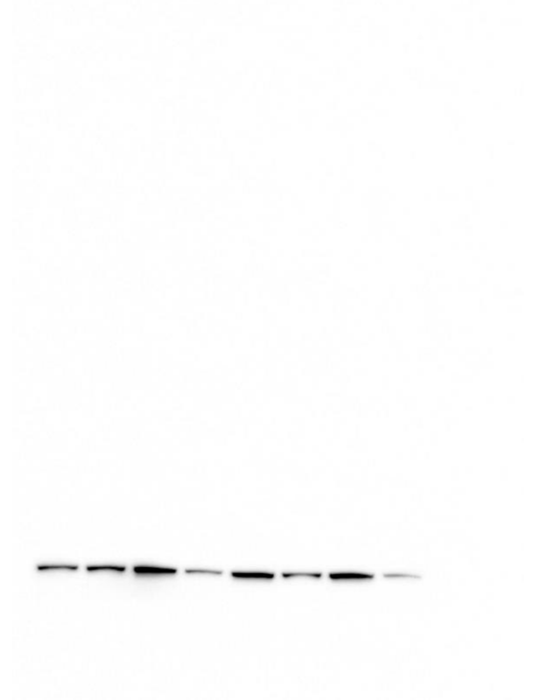
		p-AKT	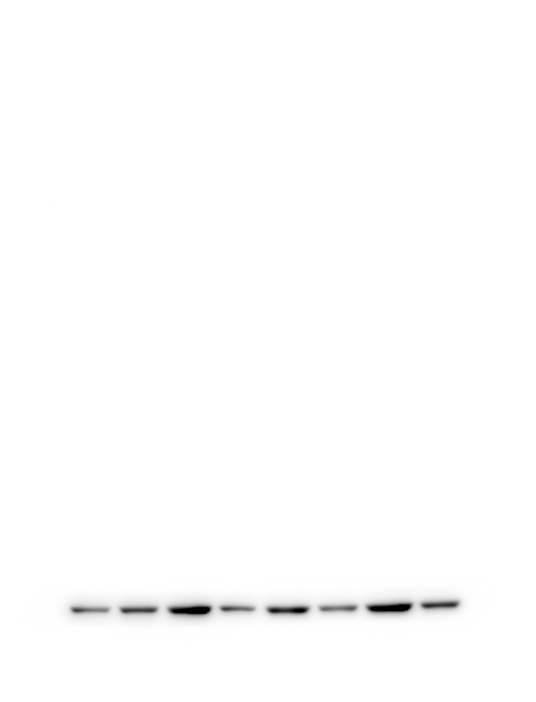

		p-mTOR	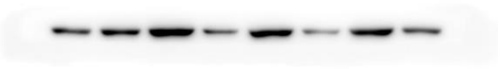
		KLF5	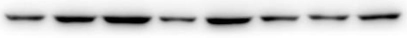

		β-Actin	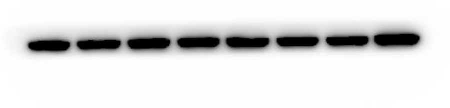
	Colon (Day 17)	p-PI3K	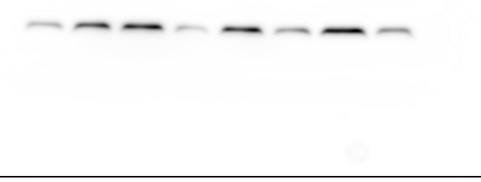

		p-AKT	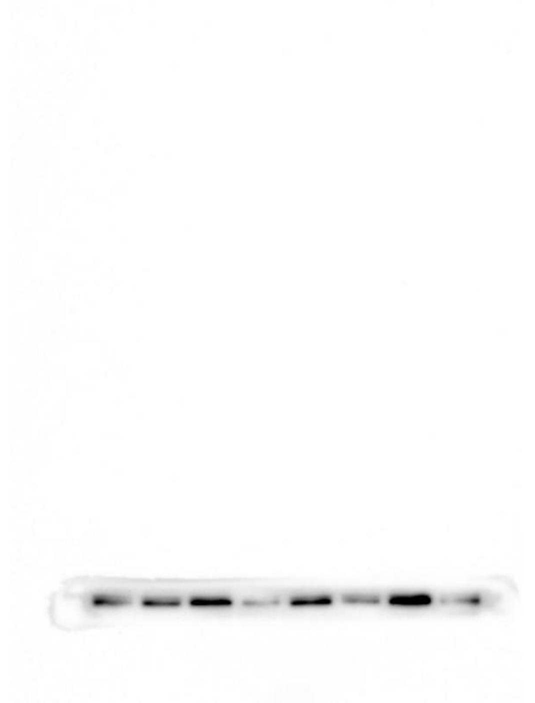
		p-mTOR	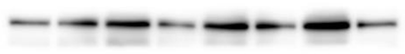

		KLF5	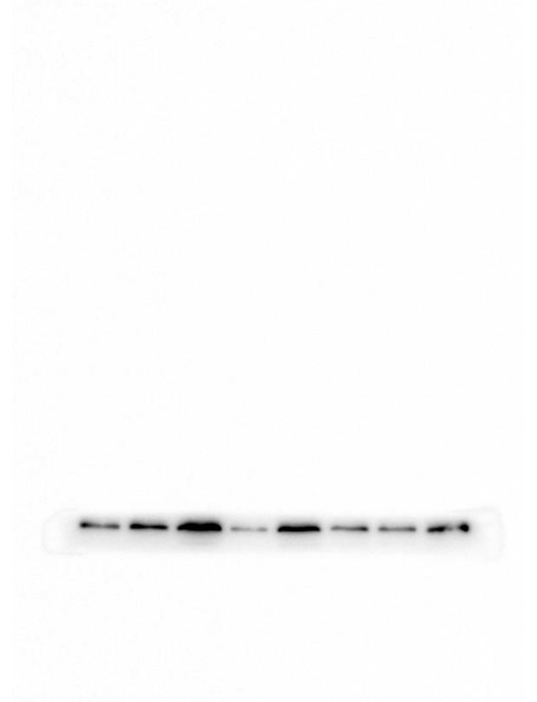
		β-Actin	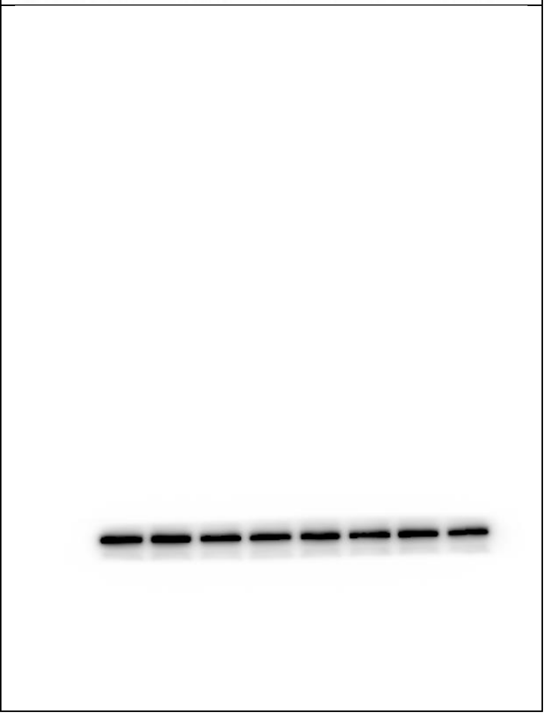

	Colon (Day 24)	p-PI3K	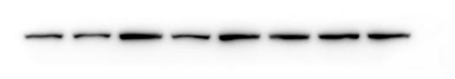
		p-AKT	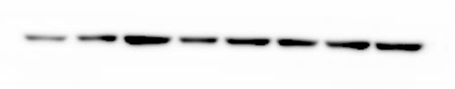

		p-mTOR	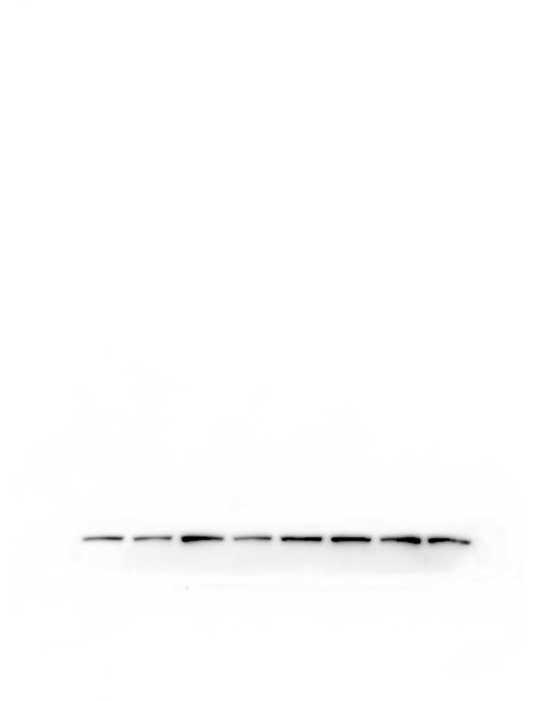
		KLF5	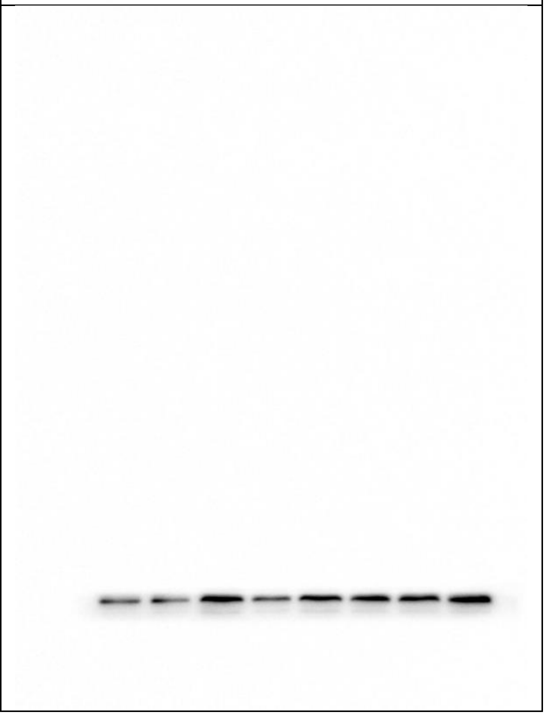

		β-Actin	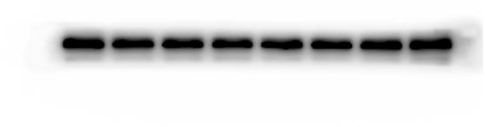
Figure 6J	Colon (Day 10)	EGF	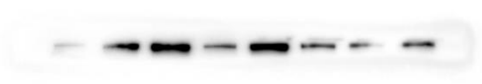

		TFF3	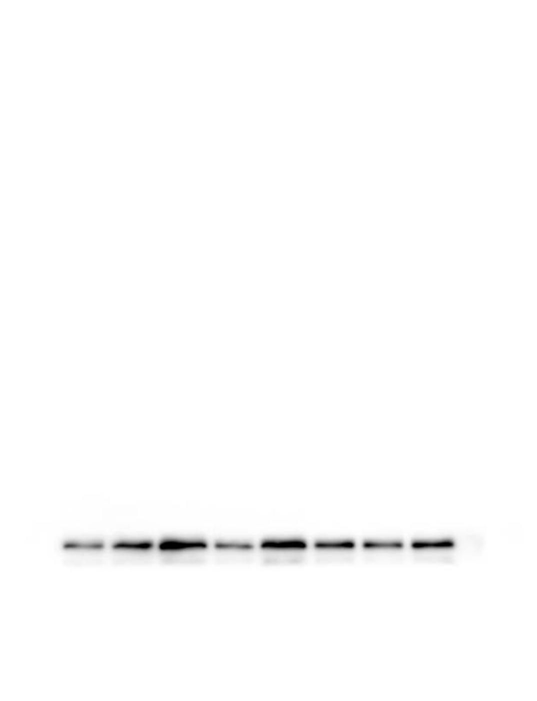
		TGF-β1	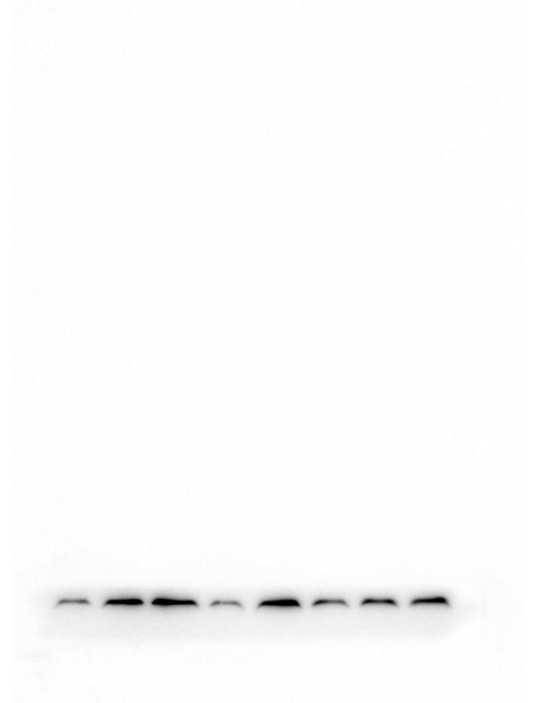

		TGF-β3	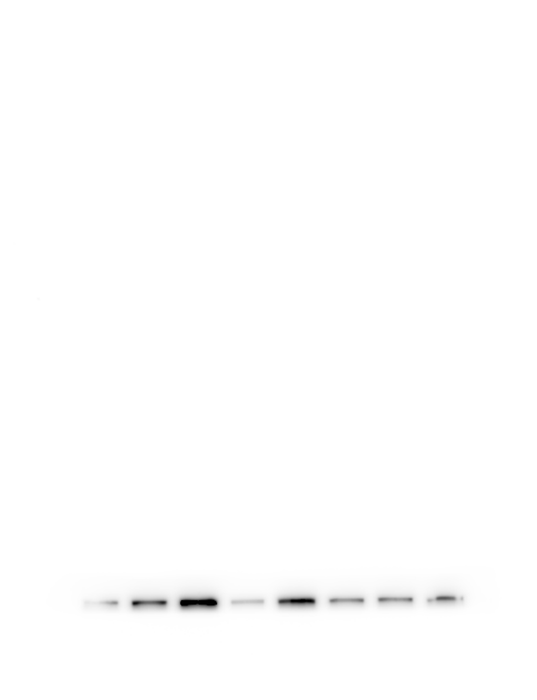
		MMP1	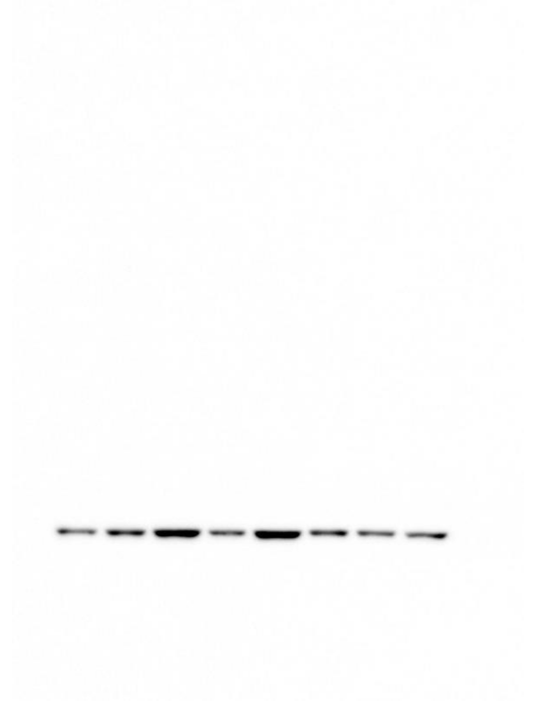

		MMP13	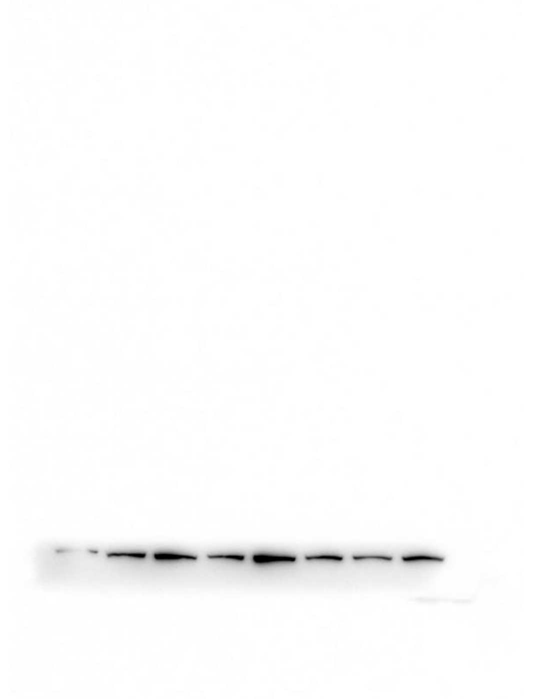
		β-Actin	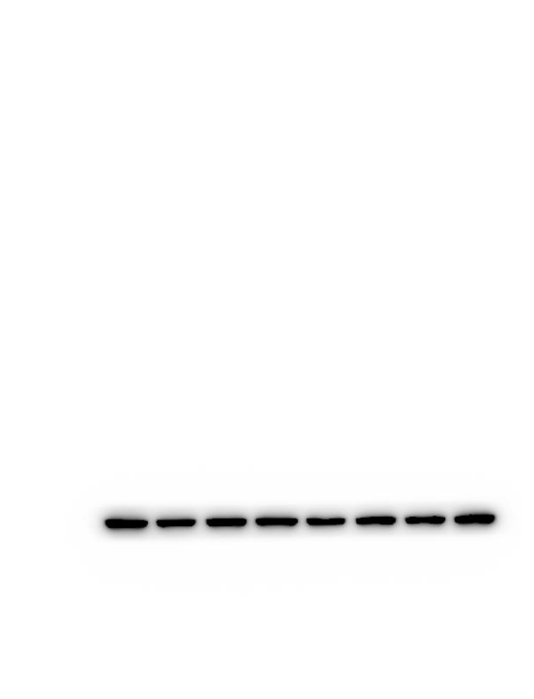

	Colon (Day 17)	EGF	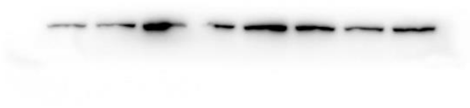
		TFF3	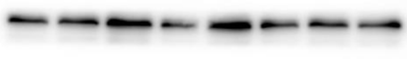

		TGF-β1	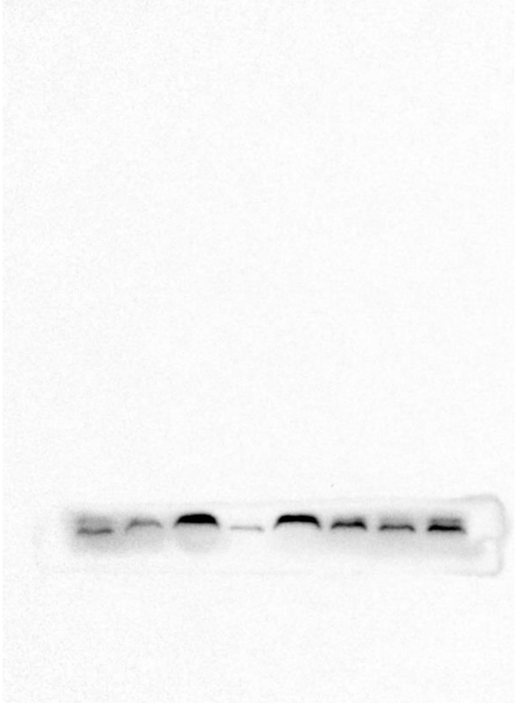
		TGF-β3	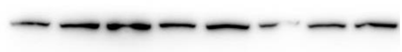

		MMP1	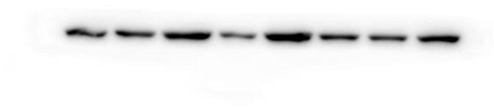
		MMP13	

		β-Actin	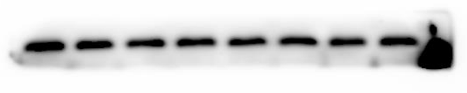
	Colon (Day 24)	EGF	

		TFF3	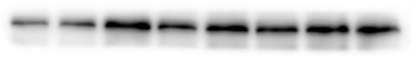
		TGF-β1	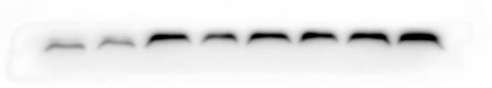

		TGF-β3	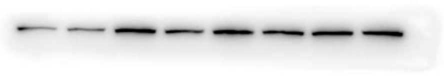
		MMP1	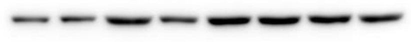

		MMP13	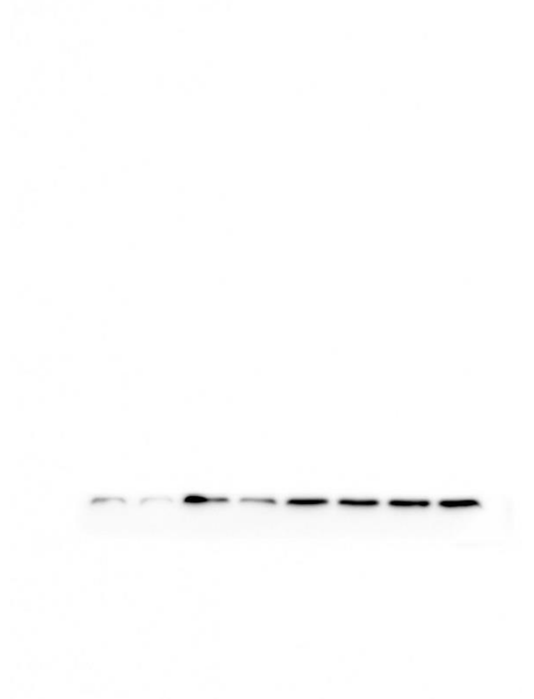
		β-Actin	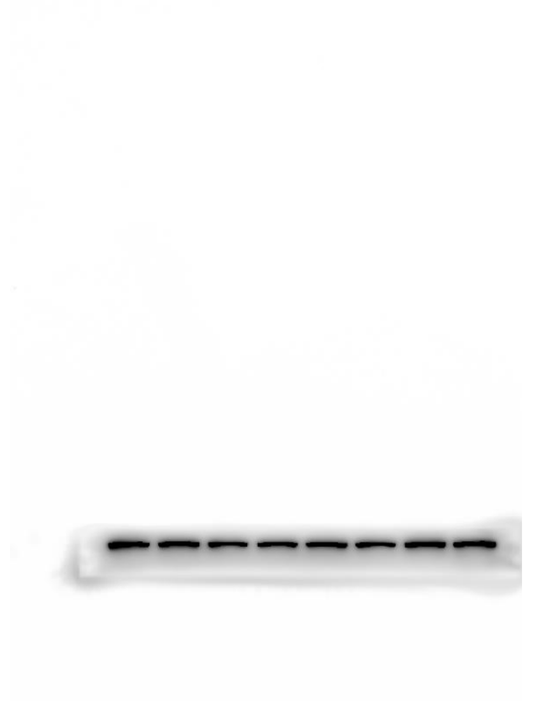

Figure S1	CCD841 CoN	GPR35	
		β-Actin	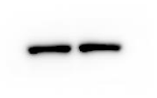

<table>
<tr><td rowspan="2"></td><td rowspan="2">FHC</td><td>GPR35</td><td></td></tr>
<tr><td>β-Actin</td><td>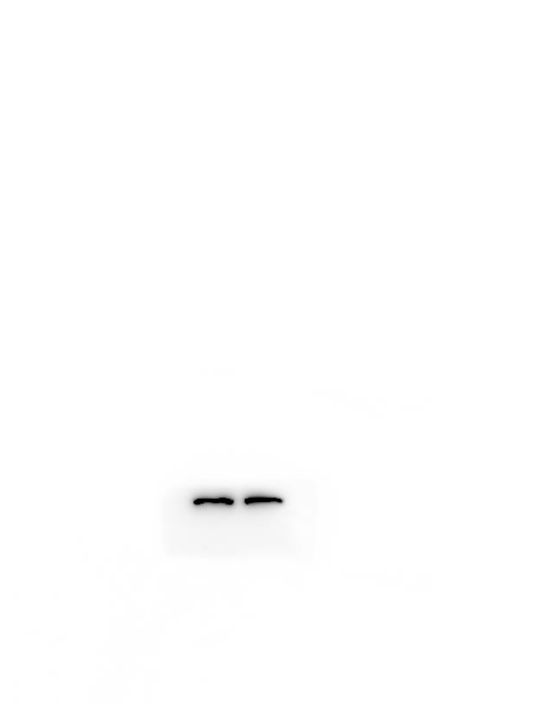</td></tr>
</table>

Figure S3		KLF5	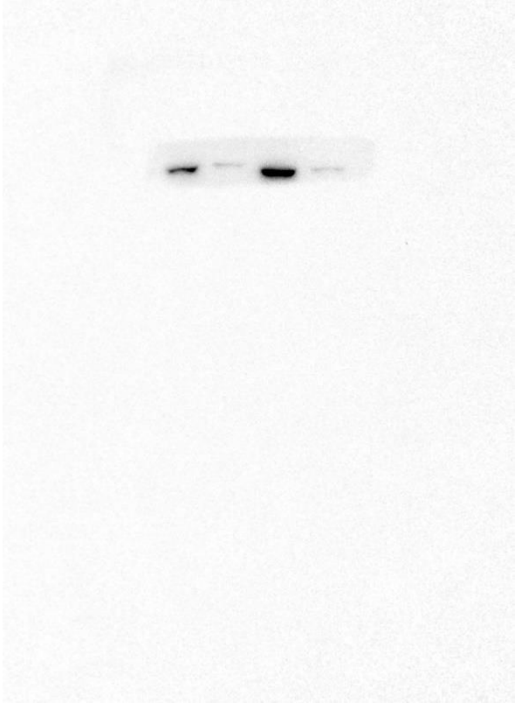
		β-Actin	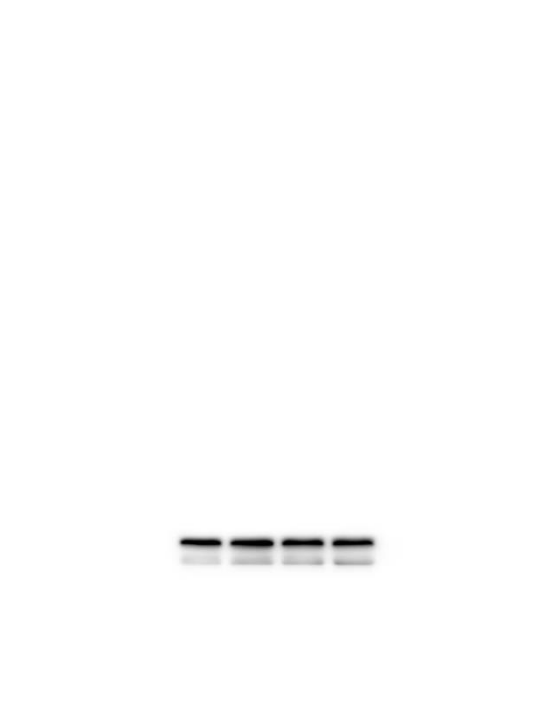

<table>
<tr><td rowspan="2">Figure S5B</td><td rowspan="2">CCD841 CoN</td><td>p-MEK1/2</td><td>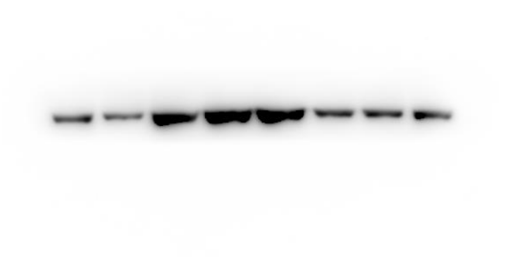</td></tr>
<tr><td>p-ERK1/2</td><td>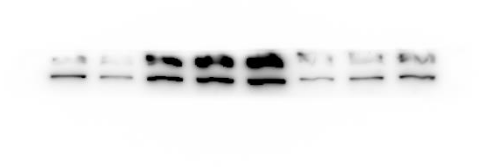</td></tr>
</table>

		KLF5	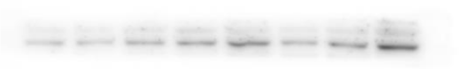
		β-Actin	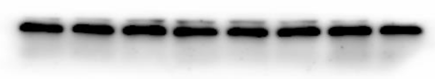

	FHC	p-MEK1/2	
		p-ERK1/2	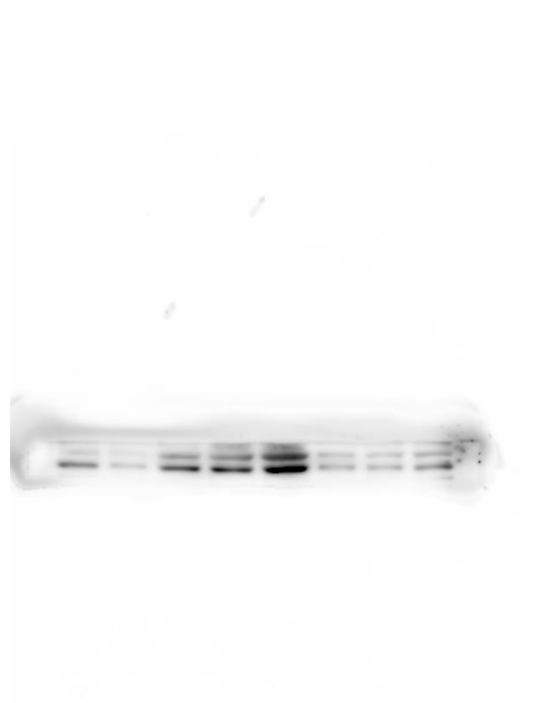

		KLF5	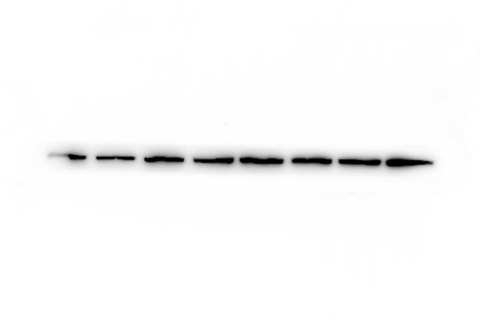
		β-Actin	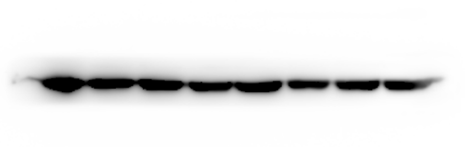

Figure S6A	CCD841 CoN	p-PI3K	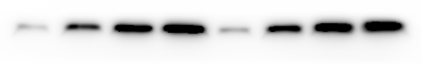
		p-AKT	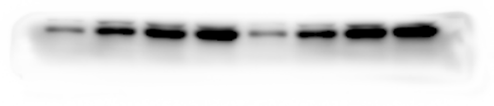

		p-mTOR	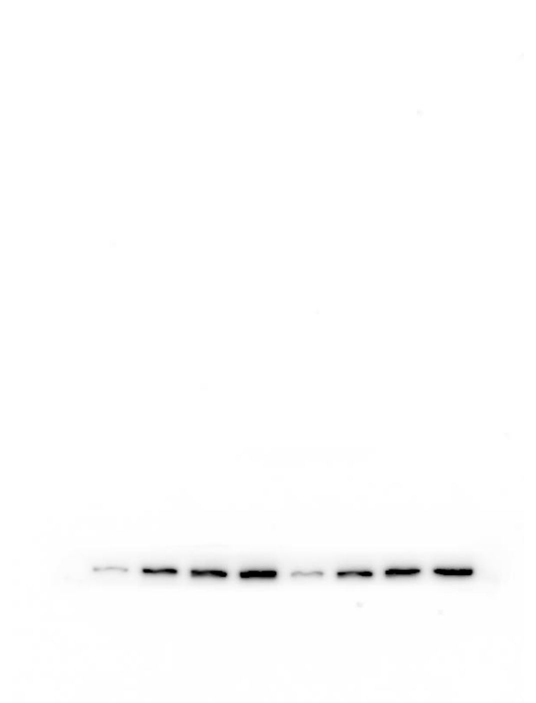
		KLF5	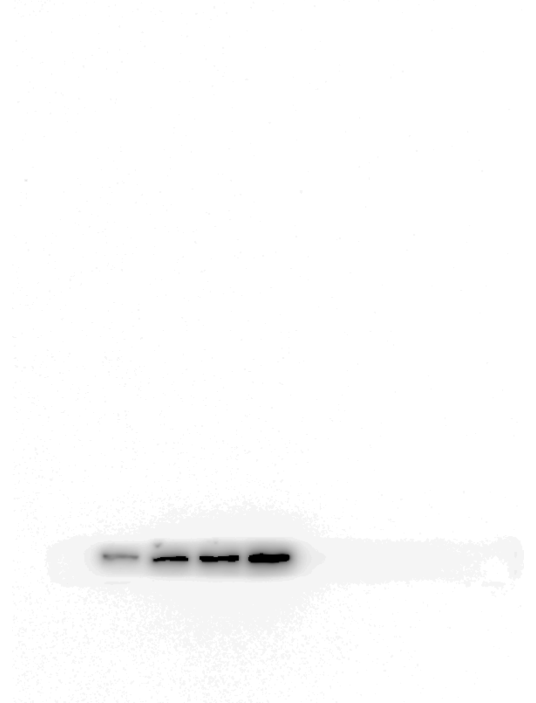

		β-Actin	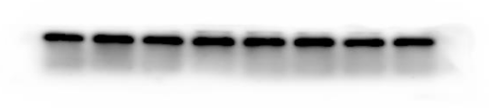
	FHC	p-PI3K	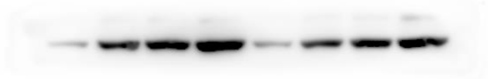

		p-AKT	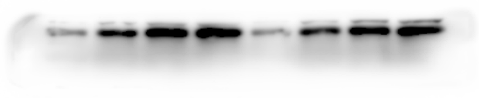
		p-mTOR	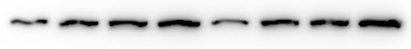

		KLF5	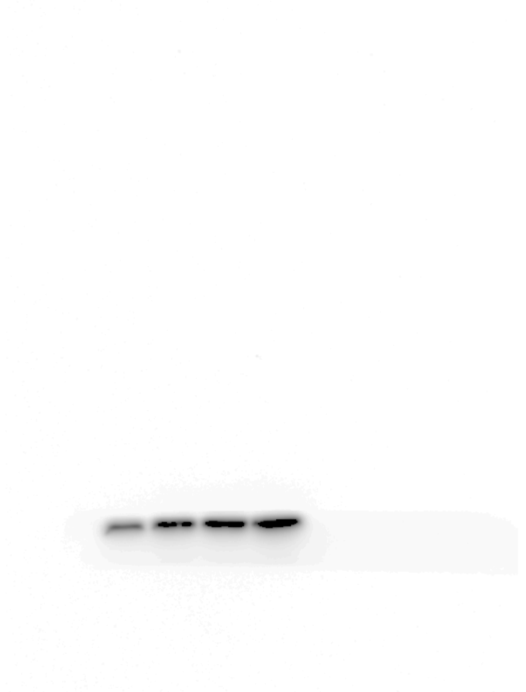
		β-Actin	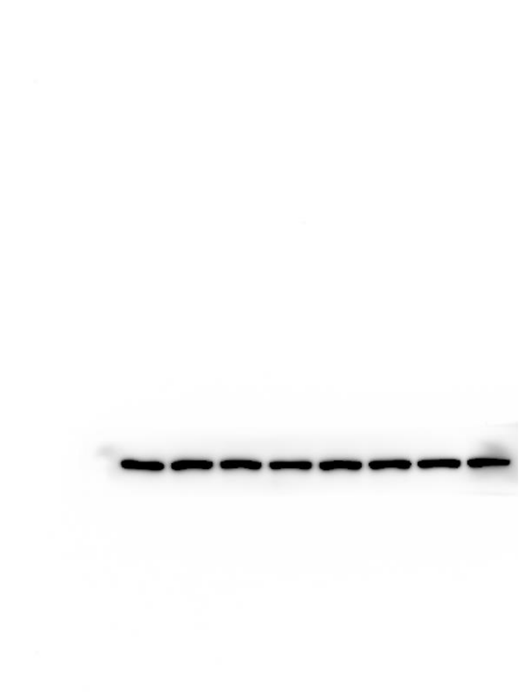

<table>
<tr><td rowspan="2">Figure S6B</td><td rowspan="2">CCD841 CoN</td><td>p-PI3K</td><td>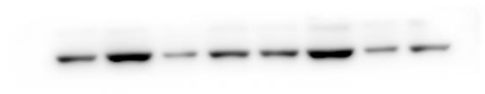</td></tr>
<tr><td>p-AKT</td><td>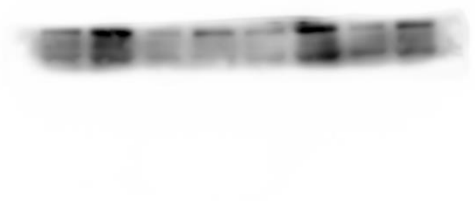</td></tr>
</table>

		p-mTOR	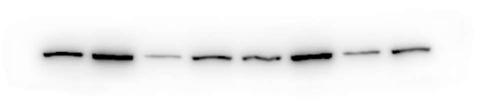
		KLF5	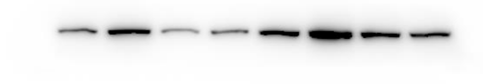

		β-Actin	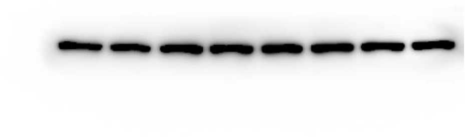
	FHC	p-PI3K	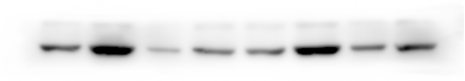

		p-AKT	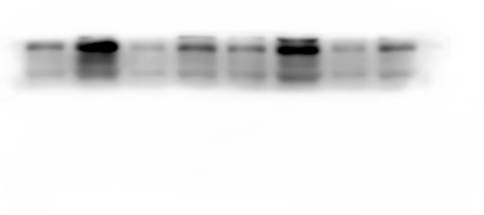
		p-mTOR	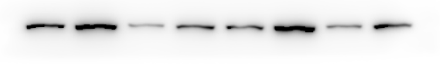

		KLF5	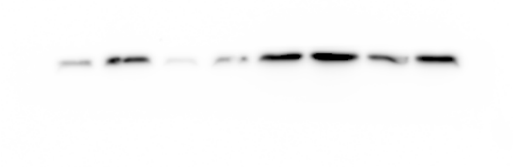
		β-Actin	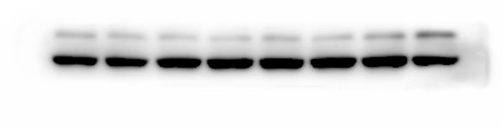

<table>
<tr><td rowspan="2">Figure S7A</td><td rowspan="2">FHC</td><td>p-ERK1/2</td><td>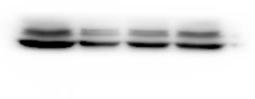</td></tr>
<tr><td>ERK1/2</td><td>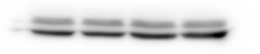</td></tr>
</table>

		GPR35	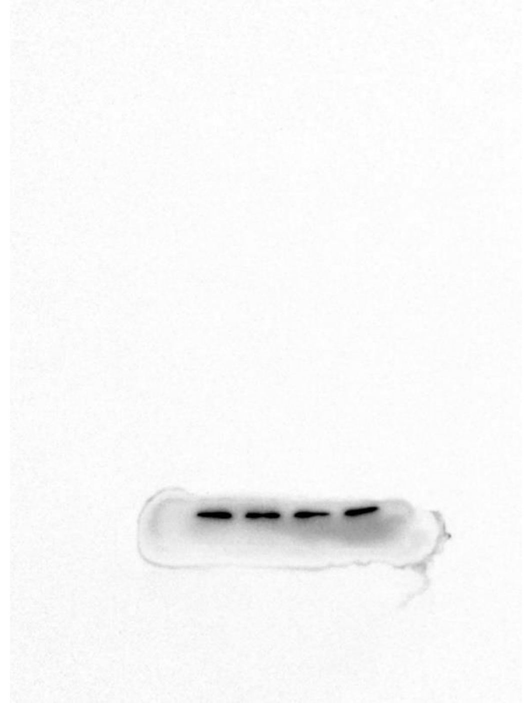
		β-Actin	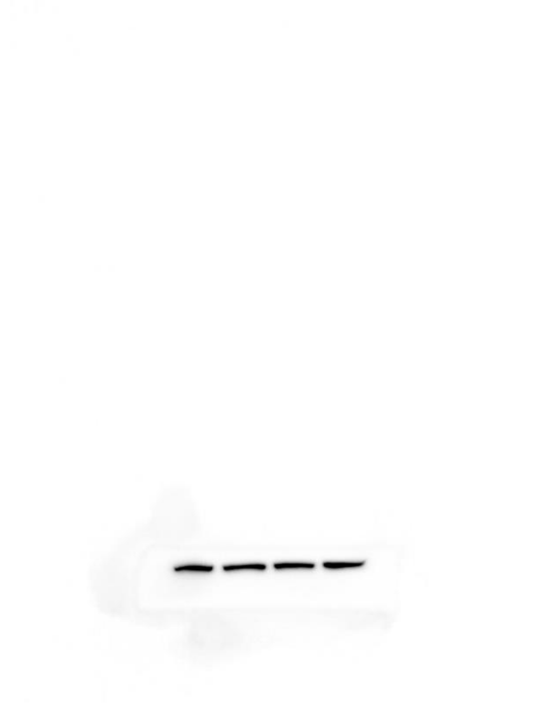

Figure S7B	FHC	p-PI3K	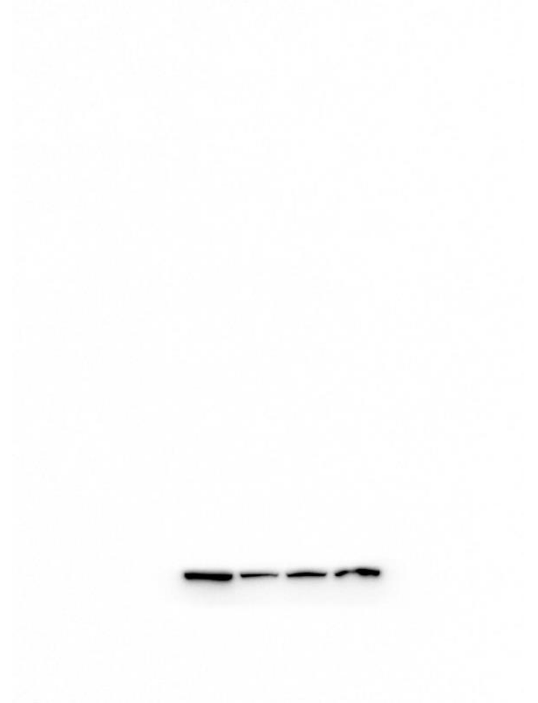
		p-AKT	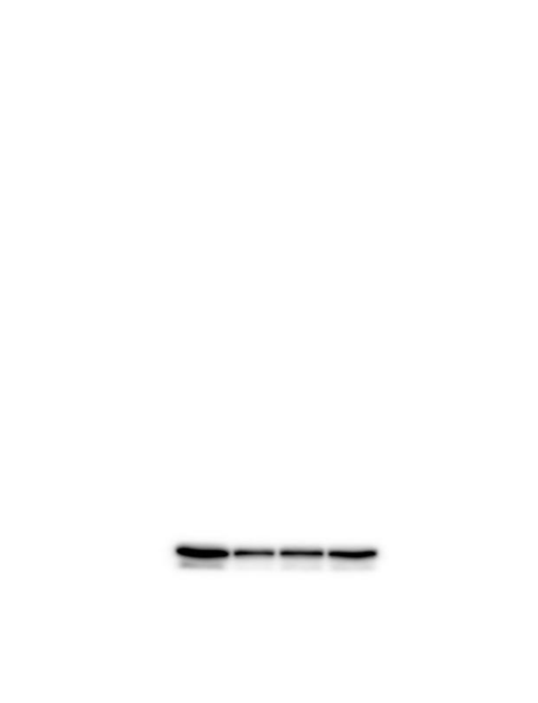

		p-mTOR	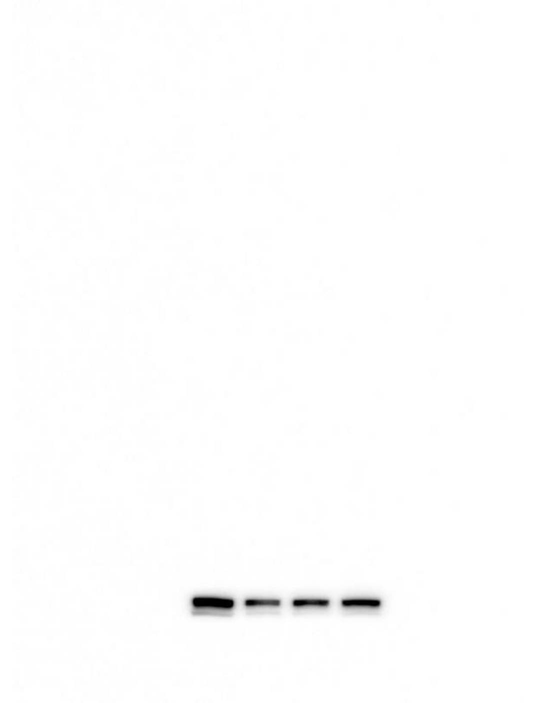
		KLF5	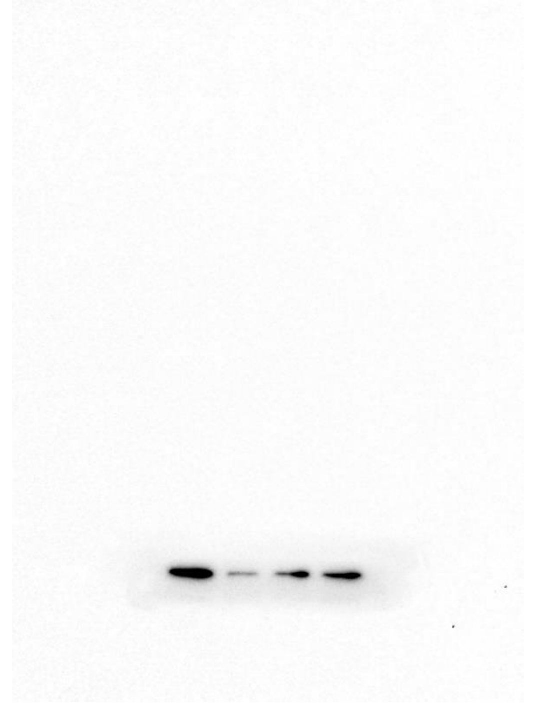

		β-Actin	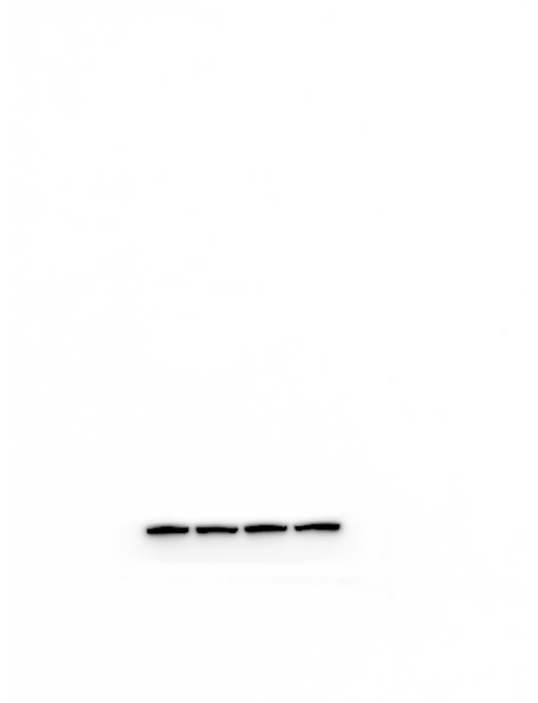
